# Association between systolic blood pressure and low-density lipoprotein cholesterol with coronary heart disease according to age

**DOI:** 10.1371/journal.pone.0295004

**Published:** 2023-12-20

**Authors:** Nelson Wang, Rima Mustafa, Verena Zuber, Anthony Rodgers, Abbas Dehghan

**Affiliations:** 1 The George Institute for Global Health, University of New South Wales, Sydney, Australia; 2 Royal Prince Alfred Hospital, Sydney, Australia; 3 Sydney Medical School, University of Sydney, Sydney, Australia; 4 Department of Epidemiology and Biostatistics, Imperial College London, London, United Kingdom; 5 UK Dementia Research Institute, Imperial College London, London, United Kingdom; 6 MRC Centre for Environment and Health, Imperial College London, London, United Kingdom; The University of Mississippi Medical Center, UNITED STATES

## Abstract

**Background:**

The impact of elevated systolic blood pressure (SBP) and low-density lipoprotein cholesterol (LDL-C) on the risk of coronary heart disease (CHD) at different stages of life is unclear. We aimed to investigate whether genetically mediated SBP/LDL-C is associated with the risk of CHD throughout life.

**Methods and findings:**

We conducted a three-sample Mendelian randomization analysis using data from the UK Biobank including 136,648 participants for LDL-C, 135,431 participants for SBP, and 24,052 cases for CHD to assess the effect of duration of exposure to the risk factors on risk of CHD. Analyses were stratified by age at enrolment. In univariable analyses, there was a consistent association between exposure to higher LDL-C and SBP with increased odds of incident CHD in individuals aged ≤55 years, ≤60 years, and ≤65 years (p-value for heterogeneity = 1.00 for LDL-C and 0.67 for SBP, respectively). In multivariable Mendelian randomization analyses, exposure to elevated LDL-C/SBP early in life (age ≤55 years) was associated with a higher risk of CHD independent of later life levels (age >55 years) (odds ratio 1.68, 95% CI 1.20–2.34 per 1 mmol/L LDL-C, and odds ratio 1.33, 95% CI 1.18–1.51 per 10 mmHg SBP).

**Conclusions:**

Genetically predicted SBP and LDL-C increase the risk of CHD independent of age. Elevated SBP and LDL-C in early to middle life is associated with increased CHD risk independent of later-life SBP and LDL-C levels. These findings support the importance of lifelong risk factor control in young individuals, whose risk of CHD accumulates throughout life.

## Introduction

Elevated low density lipoprotein cholesterol (LDL-C) and systolic blood pressure (SBP) remain the leading global contributors to coronary heart disease (CHD) [1]. Mendelian randomisation (MR) studies use genetic variants to investigate the causal relationship between a biomarker (such as LDL-C and SBP) on risk of disease (such as CHD). Prior Mendelian randomisation (MR) studies have shown very large reductions in coronary heart disease associated with lifelong exposures to lower SBP and LDL cholesterol (approximately 50% reduction in CHD per mmol/L lower LDL-C and 10 mmHg lower SBP) [2–4]. These results contrast to the smaller associations between these risk factors and CHD from randomized clinical trials of BP and cholesterol lowering therapies (approximately 20% reduction in CHD per 1 mmol/L lower LDL-C and 10 mmHg lower SBP) [5–7]. This discrepancy raises questions about the impact of duration of exposure on the relationship between these risk factors and CHD, and the potential implications for the management of these conditions.

Currently, therapeutic strategies for cholesterol and BP lowering are based on short-term clinical trials. If longer exposure to high cholesterol and blood pressure has a cumulative effect as reported by MR studies, this will have substantial implications for the management of CHD. There may be a role for earlier treatment in low-risk and younger patients if there are indeed additional benefits with cumulative risk factor control. A prior MR found consistent evidence of higher genetically predicted mean arterial pressure at age ≤55 years increasing coronary heart disease risk after adjusting for genetically predicted mean arterial pressure at age >55 years [8]. Whether this finding is consistent for both SBP and LDL-C, and whether a trend for risk across different age groups exists remains unknown. Furthermore, another recent MR analysis showed that elevated BP and LDL-C may not be associated with CHD at older ages, and concluded that BP and LDL-C lowering may not be warranted in subgroups of older patients [9]. However, this analysis was based on one-sample MR which is prone to overfitting and biased towards confounded observational estimates as the genetic associations for exposure and outcome are derived from the same sample [10].

To address these gaps in the knowledge, the current study aimed to generate genetic instruments for SBP and LDL-C at different age cut-offs and investigate whether genetically elevated SBP and LDL-C are associated with the risk of CHD throughout life. To answer the issue of whether the risk of SBP and LDL-C is dependent on the duration of exposure, we assessed the impact of SBP and LDL-C at a younger age on the risk of CHD independent of later age SBP and LDL-C. The findings of this study could have significant implications for the management of CHD and the use of early treatment in lower-risk and younger patients.

## Methods

### Study population

This study used individual-level data from participants in the UK Biobank, a large prospective cohort of ~500,000 participants in the United Kingdom [11]. We included 450,782 participants enrolled between the years 2006 and 2010 and followed up through the year 2020. We included participants of European ancestries and exclude related participants based on a kinship coefficient > 0.088, which is the proposed lower-limit kinship-coefficient threshold for a second-degree relative [12]. BP measurements were performed twice after the participant had been at rest for at least 5 minutes in the seated position with a digital sphygmomanometer (Omron 705 IT; OMRON Healthcare Europe B.V., Hoofddorp, the Netherlands) and the average of the two measurements was used for the analyses. LDL-C was measured directly in mmol/L (analytical range: 0.26–10.3) using Enzymatic Selective Protection analysis methodology using the Beckman Coulter AU5800 platform (Beckman Coulter (UK), Ltd) [13]. When participants reported the use of cholesterol-lowering medication during the assessment, LDL-C levels were divided by 0.7; when participants reported the use of blood pressure lowering medication, 15 mmHg was added to the measured systolic blood pressure [14]. We divided the population into four groups according to age at enrolment: ≤55 years, 55–60 years, 61–65 years and ≥65 years, which divided the cohort into approximate quarters. To ensure that the genetic associations for exposure and outcome in the univariable MR analysis were not overlapping, we randomly divided the participants in each age group into two subgroups for the exposure (SBP or LDL-C) and outcome (CHD) analysis. This ensured that each participant contributed only to one subgroup and that the genetic associations for exposure and outcome were derived from non-overlapping participants.

### Study outcome

The primary outcome of the study was CHD defined as a composite of coronary death, nonfatal myocardial infarction, or coronary revascularization. The exact diagnostic codes can be found in the S1 Appendix. We first combined both incident and prevalent cases of CHD to maximize power, under the implicit assumption that all events occur incident to a genetic exposure. We also looked at incident CHD only, such that each age group had at least that many years of exposure to different levels of LDL-C and SBP (i.e., at least 55 years of exposure in the age 55–60 years group).

### Statistical analysis

To assess the effect of genetically determined SBP and LDL-C levels on the risk of CHD in different age groups, we performed a three-sample univariable MR and multivariable MR analysis. Genetic instruments for SBP and LDL-C were obtained from summary statistics from the largest Genome-Wide Association Studies (GWAS) conducted by the International Consortium of Blood Pressure (ICBP) [15] and the Global Lipids Genetics Consortium (GLGC) [16], respectively. The selection of instruments from these external datasets that did not include the UK Biobank could reduce potential bias from winner’s curse. Instruments were defined as single nucleotide polymorphisms (SNPs) associated with SBP or LDL-C at P<5x10^-8^ and uncorrelated (r^2^<0.001). Analyses were stratified based on the age of enrolment to assess the effect of duration of risk factor exposure on the risk of CHD, given that genetically determined SBP and LDL-C equate to a lifelong exposure.

We obtained age-group-specific estimates for the exposures (SBP and LDL-C separately) using a linear mixed-model implemented in BOLT-LMM after dividing participants into age groups (age ≤55 years, 55–60 years, 61–65 years and ≥65 years) and ensuring no overlap of participants between the exposure and outcome [17]. The genetic estimates for CHD as the outcome was obtained using logistic regression within an independent cohort of participants in the same age group. Both linear and logistic regressions were conducted with adjustment for age, sex, and the first five genetic principal components.

We estimated the association between 1 mmol/L higher LDL-C and 10 mmHg higher SBP on the risk of CHD to allow for easier comparison with traditional pharmacological therapies for BP and cholesterol-lowering. The exposure allele for each variant was defined as the allele associated with higher SBP and LDL-C. Multiplicative random-effects inverse-variance–weighted MR was used as the main analysis to assess the effect of genetically predicted SBP and LDL-C on CHD. MR Egger and weighted median methods were used to explore the robustness of the findings to potential pleiotropic variants [18]. Agreement among different MR methods supports a robust estimation of causal effects. In the main analysis, we used the same set of instruments from ICBP and GLGC for all age groups and obtained age-specific estimates for those instruments. We then excluded any weak instruments (F-statistics<10). Finally, MRPRESSO was used to obtain corrected estimates in the presence of outliers among instruments [19]. Conditional F-statistics were calculated to assess the strength of instruments in the multivariable MR analysis [20].

Univariable MR analyses were conducted to estimate the association between LDL-C and SBP across the four age groups. First, we assessed the association between the exposure and total CHD (incident and prevalent cases) within the same age group, i.e. genetically predicted LDL-C and SBP at age ≤55 on prevalent and incident CHD risk in the same cohort. Given different ages of prevalent CHD may result in different durations of risk factor exposure, we further assessed the association of genetically predicted LDL-C and SBP on the risk of incident CHD after the age of enrolment, i.e., the association between genetically predicted LDL-C and SBP at age ≤55 years on CHD risk after age 55 years, LDL-C and SBP at age ≤60 years on CHD risk after age 60 years, and LDL-C and SBP at age ≤65 years on CHD risk after age 65 years.

To estimate the effect of genetically predicted SBP and LDL-C at age ≤55 years on total CHD risk independent of genetically predicted SBP and LDL-C at age ≥55 years, multivariable MR was performed. Age 55 years was chosen to divide the UK Biobank cohort into approximately half. For the multivariable MR, genetic instruments were selected as independent genetic variants associated with at least one of the exposures (risk factor at certain age groups) at P<1x10^-5^.

All analyses were performed using R (version 3.2.2; R Project for Statistical Computing). A 2-tailed P value less than .05 was considered statistically significant. The data used in these analyses are publicly available. The UK Biobank study was approved by the North West Multicentre Research Ethics Committee, and all its participants provided informed written consent that was witnessed by a staff member. All accessed data from the UK Biobank were deidentified. The UK Biobank phenotype data were accessed through application 52569, and the genetic data were from application 236.

## Results

A total of 450,782 participants in the UK Biobank were considered in our analysis. Our analysis included 136,648 participants for LDL-C and 135,431 participants for SBP. Table 1 shows the baseline characteristics according to the age of enrolment, with four groups: age ≤55 years, 55–60 years, 61–65 years and ≥65 years. Mean SBP increased with increasing age, and the use of antihypertensives followed a similar trend. LDL-C was highest in those aged 56–60 years, however, the use of lipid-lowering therapy increased with older age. For CHD, there was a total of 24,052 cases (incident and prevalent), with most cases occurring over the age of 60 years.

**Table 1:** Patient characteristics according to age at enrolment.

	Systolic blood pressure				Low-density lipoprotein cholesterol			
	age ≤ 55	age 56–60	age 61–65	age > 65	age ≤ 55	age 56–60	age 61–65	age > 65
Number	56,710	27,035	32,603	19,083	57,549	27,279	32,611	19,209
Age, y (mean ± SD)	48 ± 4	58 ± 1	63 ± 1	67 ± 1	48 ± 4	58 ± 1	63 ± 1	67 ± 1
Males, No. (%)	24,769 (44)	11,613 (43)	14,428 (44)	8,892 (47)	25,056 (43.5)	11,729 (43.0)	14,395 (44.1)	8,953 (46.6)
Body mass index, kg/m^2^ (mean ± SD)	27 ± 5	27 ± 5	27 ± 5	27 ± 4	27 ± 5	27 ± 5	27 ± 5	27 ± 4
Smoker, No. (%)								
Current	7,155 (13)	2,616 (10)	2,629 (8)	1,320 (7)	7,227 (13)	2,644 (10)	2,637 (8)	1,339 (7)
Former	15,819 (28)	9,897 (37)	13,397 (41)	8,148 (43)	16,039 (28)	9,928 (36)	13,460 (41)	8,166 (43)
Never	33,602 (59)	14,421 (53)	16,447 (50)	9,505 (50)	34,141 (59)	14,607 (54)	16,386 (50)	9,596 (50)
LDL-C, mmol/L (mean ± SD)	3.5 ± 0.8	3.7 ± 0.9	3.6 ± 0.9	3.6 ± 0.9	3.5 ± 0.8	3.7 ± 0.9	3.6 ± 0.9	3.6 ± 0.9
SBP, mmHg (mean ± SD)	133 ± 17	141± 19	145 ± 19	149 ± 20	133 ± 17	141 ± 19	145 ± 19	149 ± 20
DBP, mmHg (mean ± SD)	82 ± 11	83 ± 11	83 ± 10	82 + 10	82 ± 11	83 ± 11	83 ± 10	82 ± 10
Current lipid-lowering treatment, No. (%)	3,244 (6)	3,895 (14)	7,159 (22)	5,533 (29)	3,279 (6)	3,921 (14)	7,187 (22)	5,552 (29)
Current BP lowering treatment, No. (%)	4,737 (8)	5,186 (19)	8,727 (27)	6,410 (34)	4,848 (8)	5,259 (19)	8,788 (27)	6,457 (34)
CHD cases (prevalent and incident)	3,826	4,423	8,189	7,614	3,826	4,423	8,189	7,614

BP, blood pressure; CHD, coronary heart disease; DBP, diastolic blood pressure; LDL-C, low density lipoprotein cholesterol; SBP, systolic blood pressure.

All instruments and genetic association estimates used in the MR analyses are provided in S1 Table in S1 Appendix. The correlation of genetic association estimates across age groups were relatively high (range: 0.90–0.96 for LDL-C and 0.77–0.83 for SBP).

### Genetically predicted SBP and LDL-C and total CHD

**Fig 1:**
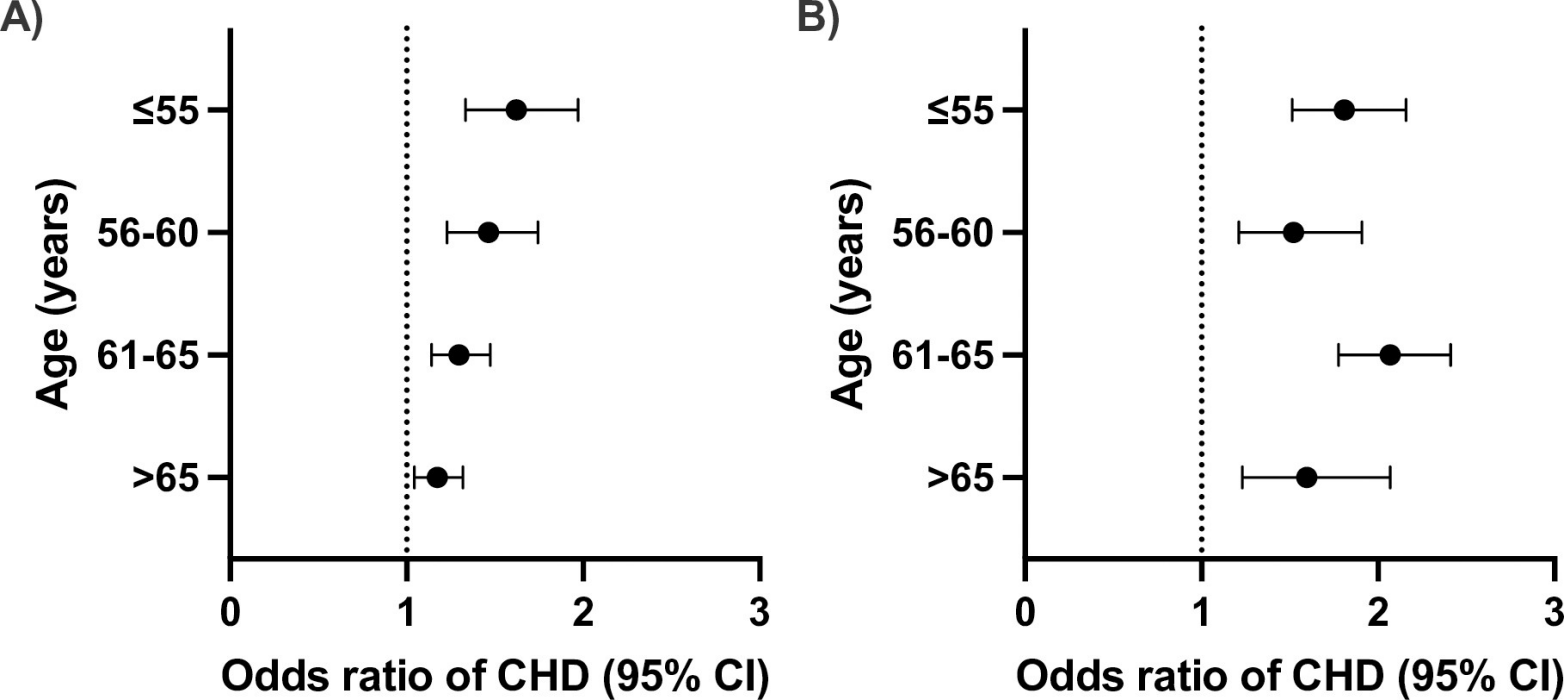
Effect of systolic blood pressure (SBP) and low-density lipoprotein cholesterol (LDL-C) on total coronary heart disease (CHD) at different ages. Odds ratio of total CHD for genetically predicted A) SBP and B) LDL-C in univariable Mendelian randomization analyses. The circle and error bars represent the odds ratio and their 95% confidence intervals. All effect estimates are given per 10 mmHg increase in SBP and 1 mmol/L increase in LDL-C.

The results from the univariable MR showed a significant association between genetically predicted LDL-C and risk of CHD across all four age groups with no evidence of significant heterogeneity between groups (p-value for heterogeneity = 0.107) (Fig 1). Each 1 mmol/L higher LDL-C was associated with 81% higher odds of CHD at age ≤55 years, 52% higher odds at age 55–60 years, 106% higher odds at age 61–65 years and 60% higher odds at age ≥65 years. We found an age-dependent association for SBP, such that the effect of genetically predicted SBP on CHD decreased with older age (p-value for heterogeneity = 0.023). For each 10 mmHg increase in genetically mediated SBP, the odds of CHD increased by 62% for individuals aged ≤55 years, 46% for those aged 55–60 years, 30% for those aged 61–65 years and 17% for those aged ≥65 years (Fig 1). Similar results were obtained when performing MR Egger and weighted median methods, which each make different assumptions about the potential inclusion of pleiotropic variants that affect CHD risk through pathways unrelated to SBP and LDL-C (S1 Fig in S1 Appendix). Further sensitivity analysis that excluded outliers by MRPRESSO also showed consistent results (S1 Fig in S1 Appendix).

### Genetically predicted SBP and LDL-C and incident CHD

**Fig 2:**
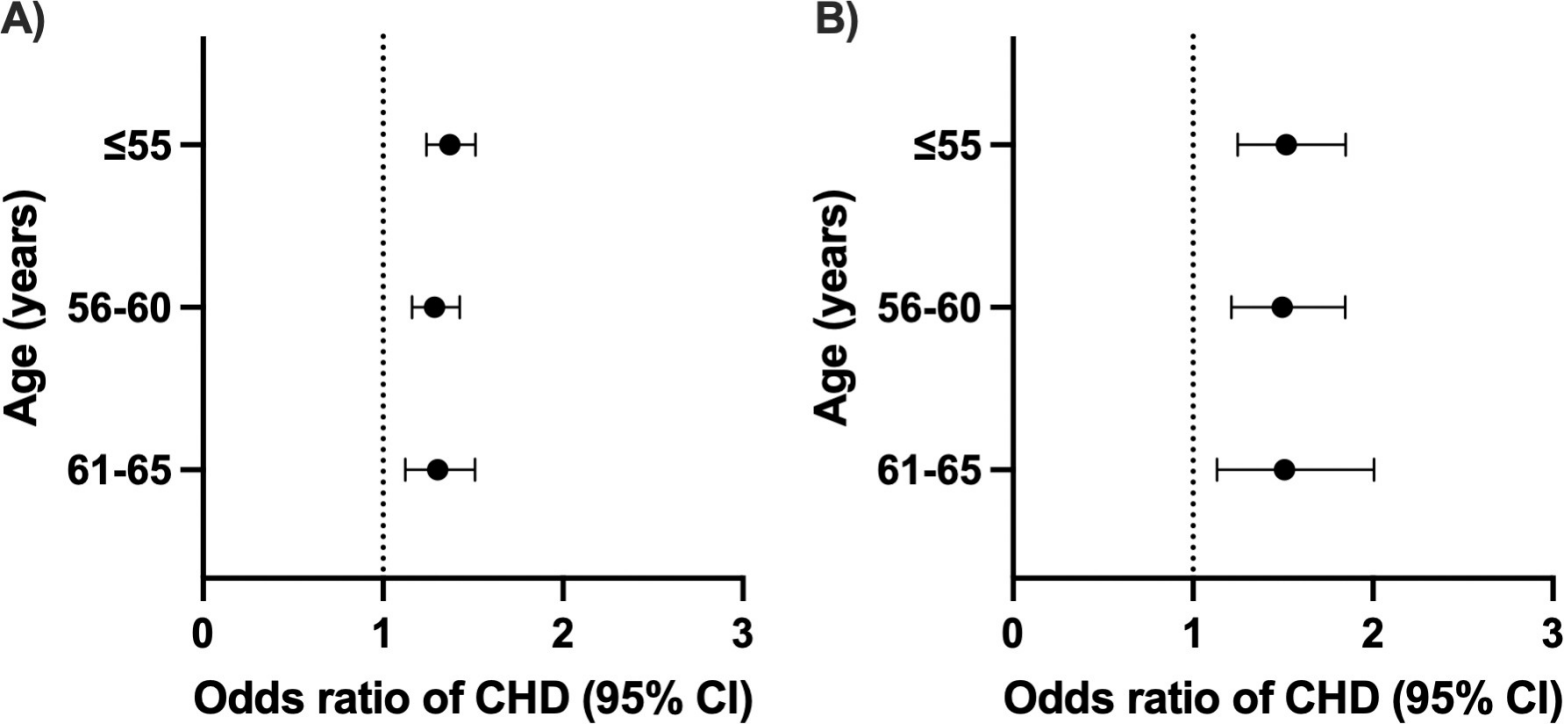
Effect of systolic blood pressure (SBP) and low-density lipoprotein cholesterol (LDL-C) on incident coronary heart disease (CHD) at different ages. Odds ratio of total CHD for genetically predicted A) SBP and B) LDL-C in univariable Mendelian randomization analyses. The circle and error bars represent the odds ratio and their 95% confidence intervals. All effect estimates are given per 10 mmHg increase in SBP and 1 mmol/L increase in LDL-C.

Lifetime exposure to genetically predicted SBP and LDL-C was strongly associated with the risk of developing incident CHD in those patients aged ≤55 years, ≤60 years and ≤65 years. Lifelong exposure to 1 mmol/L lower LDL-C was associated with a 52% increase in the odds of incident CHD later in life in those aged ≤55 years (OR 1.52, 95% CI 1.25–1.85), 50% in those aged ≤60 years (OR 1.50, 95% CI 1.21–1.85) and 51% in those aged ≤65 years (OR 1.51, 1.13–2.00) without significant heterogeneity between groups (p-value for heterogeneity = 0.995) (Fig 2). For SBP, each 10 mmHg higher SBP was associated with a similar increase in odds of CHD at age ≤55 years (OR 1.30, 95% CI 1.12–1.51), age ≤60 (OR 1.29, 1.16–1.43) and age ≤65 (OR 1.37, 95% CI 1.24–1.51) (p-value for heterogeneity = 0.667) (Fig 2). Similar results were obtained when performing MR egger and weighted median methods (S2 Fig in S1 Appendix). Further sensitivity analysis that excluded outliers by MRPRESSO also showed consistent results (S2 Fig in S1 Appendix).

### Genetically predicted SBP and LDL-C at early to middle age on CHD independent of later life levels

**Fig 3:**
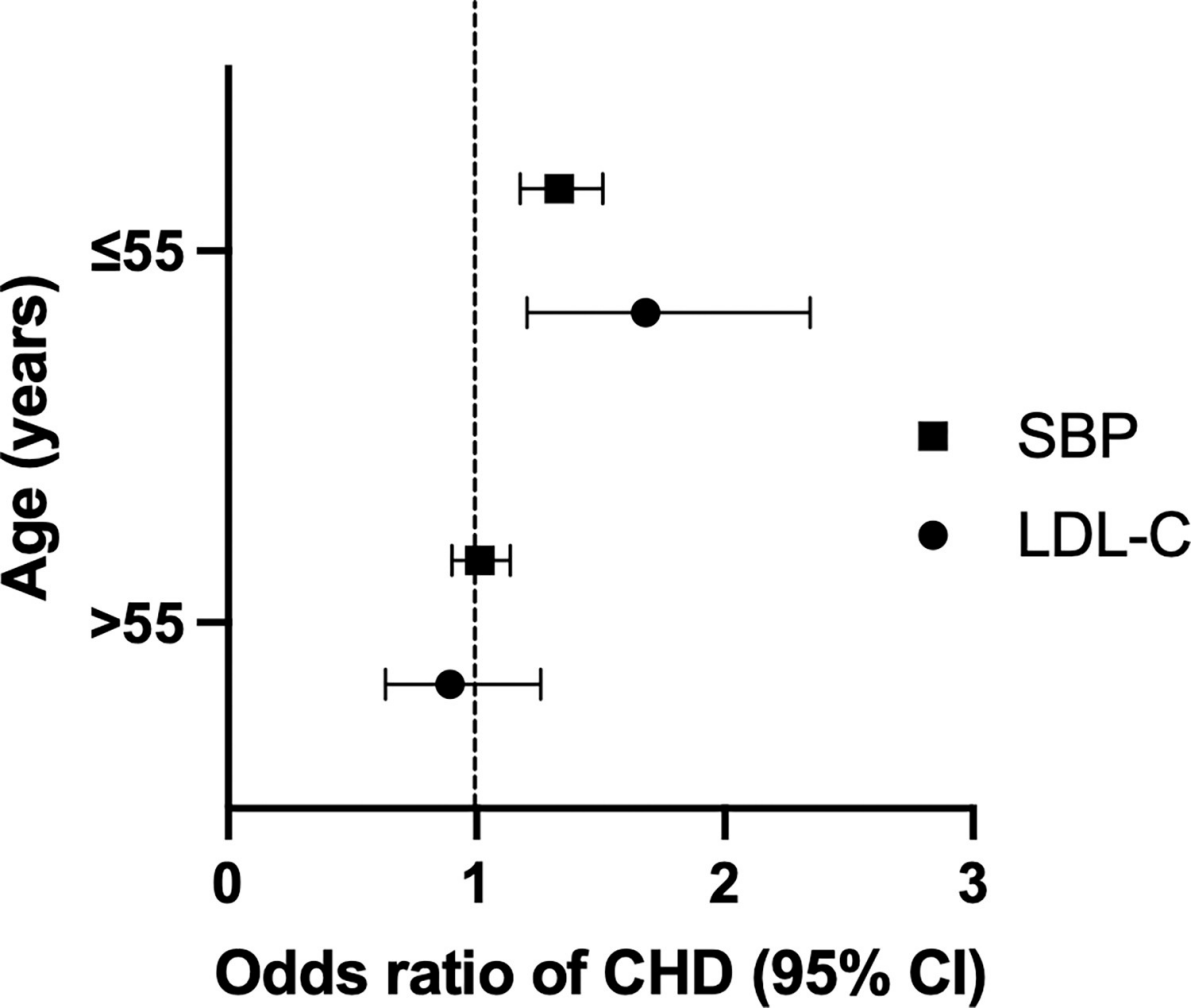
Multivariable Mendelian randomization analyses for the effect of systolic blood pressure (SBP) and low-density lipoprotein cholesterol (LDL-C) on total coronary heart disease (CHD). Odds ratio of total CHD for genetically predicted SBP and LDL-C in multivariable Mendelian randomization analyses. Square represents the estimate for SBP, whilst circle represents the estimate for LDL-C, with error bars representing the 95% confidence intervals. All effect estimates are given per 10 mmHg increase in SBP and 1 mmol/L increase in LDL-C.

Exposure to elevated SBP and LDL-C early in life appears to increase the risk of CHD independent of later-life SBP and LDL-C. Elevated SBP at age ≤55 years, was associated with increased odds of CHD after adjusting for genetically mediated SBP at age >55 years (OR 1.33, 95% CI 1.18–1.51) (Fig 3, S5 Table in S1 Appendix). Similarly elevated LDL-C at age ≤55 years was associated with increased CHD, independent of genetically mediated LDL-C at age >55 years (OR 1.68, 95% CI 1.20–2.34) (Fig 3, S5 Table in S1 Appendix). Genetically mediated SBP and LDL-C levels at later life (age >55 years) did not have a significant impact on the risk of CHD after adjusting for their effect at a younger age. However, this finding should be interpreted with caution as the conditional F-statistics in our multivariable MR analysis appeared low (S5 Table in S1 Appendix).

## Discussion

Based on our MR analyses, we found that genetically predicted SBP and LDL-C increase the risk of incident CHD throughout life, and there appeared to be no attenuation for the effect of SBP and LDL-C on the risk of CHD when one considers incident cases. The present study also found that both SBP and LDL-C in early to middle life (age ≤55 years) are associated with increased risk of CHD later in life, independent of later life SBP and LDL-C levels. These findings highlight the critical role of cumulative exposure to these risk factors in an individual’s risk of CHD, indicating that past exposure to elevated SBP and LDL-C has long-lasting implications on future CHD risk.

We found that the effects of SBP on total CHD cases (incident and prevalent) diminished with older age. This may be explained by the diminishing effects of genetics later in life [21]. For LDL-C, the effects of LDL-C on total CHD were stable with age, which may be due to a mixture of diminishing genetics and the cumulative effect of exposure to high LDL-C. When we limited our analyses to incident cases, which ensure a lifelong exposure to the differing LDL-C and SBP levels at each age group, we found no evidence of heterogeneity between the association of LDL-C and SBP and risk of incident CHD. These results contrast with a previous MR analysis that showed elevated BP and LDL-C may not be associated with CHD at older ages and concluded that BP and LDL-C lowering may not be warranted in the subgroups of older patients [9]. In this study, the authors used the same weights across all age groups and stratified the participants by age at diagnosis. This analysis was based on one-sample MR which is more prone to overfitting as the genetic associations for exposure and outcome are derived from the same sample, compared to three-sample MR conducted in this study [10].

Our findings suggest that old age alone should not be a reason to withhold otherwise appropriate LDL-C and BP-lowering treatments, because the effect of genetically mediated LDL-C and SBP on the incident risk of CHD is consistent throughout life. Our findings are consistent with randomized controlled trials that suggest the benefits of antihypertensives, and statins are consistent, even in the elderly [22, 23]. Despite this, the use of statins and BP-lowering treatment in the community declines with older age [24, 25]. Some recent guidelines continue to recommend less aggressive BP control in older patients, despite these patients being at higher absolute cardiovascular risk [26]. Of course, the decision to commence or continue BP and cholesterol-lowering therapies should ultimately be based on harm-to-benefit trade-offs.

Previously published randomized clinical trials of BP and cholesterol-lowering therapies, have suggested that a 10 mmHg decrease in SBP is associated with an approximately 20% reduction in CHD, and a 1 mmol/L LDL-C decrease is associated with a 22% reduction in CHD [5–7]. These trials had a treatment duration of 5 to 10 years of follow-up. We have previously shown that increasing durations of LDL-C lowering in randomized trials are associated with increased risk reductions in CHD for each mmol/L lowered, findings that are potentially consistent with the very large reductions in CHD associated with lifelong exposure to lower LDL-C in MR studies [27]. The present study adds to this concept by showing that exposure to elevated LDL-C and SBP earlier in life (i.e. age ≤55 years) has long-lasting effects on the risk of CHD, independent of later life LDL-C and SBP levels. We also found that genetically mediated LDL-C and SBP at age >55 years did not affect the risk of CHD at age >55 years after accounting for LDL-C and SBP at age ≤55 years. This supports the hypothesis that one’s risk of CHD is dependent on the cumulative burden of exposure to a risk factor (i.e. mmHg-years for SBP and mmol/L-years for LDL-C). This suggests that risk algorithms that consider an individual’s present-day risk factor level only may not accurately reflect one’s cumulative cardiovascular risk [28, 29]. These findings are consistent with a prior MR analysis that showed midlife mean arterial pressure is a predictor of CHD independent of later-life BP [8].

These findings also reinforce the importance of controlling elevated SBP and LDL-C early in life because young individuals with elevated risk factors have decades of future exposure to the elevated risk factor compared to an older patient. Indeed, the use of pharmacological BP and cholesterol lowering in young individuals may raise concerns about long-term safety, the economical and psychological burden of treatment in the young and the lack of randomized trial data in this population. Nevertheless, our study provides important insights into this area, particularly given the conduct of a randomized trial in a young population that spans several decades will be impractical.

Our study has several limitations to discuss. The use of antihypertensive and lipid-lowering medications varied between the different age groups. This has been accounted for by adjusting measured SBP and LDL-C based on the participant’s treatment status. It was not possible to exclude the possibility that some of our analyses might have been influenced by weak instrument bias. However, this limitation would bias the results towards the smaller or null effect of SBP and LDL-C on CHD in univariable analyses. In multivariable MR, the weak instruments could bias the magnitude of estimates and could occur in either direction [30]. We demonstrated the existence of strong genetic correlation for exposures across age groups. This suggests that our exposures are associated with the same genetic liability, i.e., the genetic instruments were not able to distinguish exposure in early versus later life, which could have contributed to the null effect observed in older age in multivariable MR.

In conclusion, genetically predicted SBP and LDL-C increase the risk of CHD independent of age. Exposure to elevated SBP and LDL-C in early to middle life is associated with increased CHD risk independent of later-life SBP and LDL-C levels. These findings support the importance of lifelong risk factor monitoring, and treatment should not be withheld in the elderly based on age alone. Greater emphasis is needed on the treatment of young individuals with elevated SBP and LDL-C, whose risk of CHD accumulates throughout life.

## Supporting information

S1 Appendix Supplemental materials containing Tables S1-S5 and Figs S1 and S2. (DOCX)
