# Supplementary material for: Association between systolic blood pressure and low-density lipoprotein cholesterol with coronary heart disease according to age
Source: PLoS One. 2023 Dec 20;18(12):e0295004. doi: 10.1371/journal.pone.0295004 (PMC10732424; doi:10.1371/journal.pone.0295004)
Supplement: S1 Appendix — (DOCX) [file pone.0295004.s001.docx]

**Appendix:**

**Title: Association between systolic blood pressure and low density lipoprotein cholesterol with coronary heart disease according to age**

**Contents:**

Definition of coronary heart disease

S1 Table. Genetic instruments used for SBP for analyses of total CHD

S2 Table. Genetic instruments used for LDL-C for analyses of total CHD

S3 Table. Genetic instruments used for SBP for analyses of incident CHD

S4 Table. Genetic instruments used for LDL-C for analyses of incident CHD

S5 Table. Multivariable MR with conditional F-statistics between SBP and LDL-C with risk of CHD

Supplemental Figure 1. Effect of genetically predicted SBP and LDL-C at different ages on risk of total CHD in univariable mendelian randomization analyses. Sensitivity analyses for MR Egger, weighted median methods and excluding outliers by MRPRESSO. All effect estimates are given per 10 mmHg increase in SBP and 1 mmol/L increase in LDL-C

Supplemental Figure 2. Effect of genetically predicted SBP and LDL-C at different ages on risk of incident CHD in univariable mendelian randomization analyses. Sensitivity analyses for MR Egger, weighted median methods and excluding outliers by MRPRESSO. All effect estimates are given per 10 mmHg increase in SBP and 1 mmol/L increase in LDL-C. There were no outliers detected by MR presso for SBP

Definition of coronary heart disease: composite of first occurrence of non-fatal myocardial infarction, coronary revascularization or coronary death.

Diagnostic codes: ICD9: 410.X, 411.0.X, 412.X, 429.79; ICD10: I21.X, I22.X, I23.X, I24.1, I25.2; self-report 20002: 1075; self-report 20004: 1070, 1095, 1523; Procedures (OPCS): K50.1, K40.X, K41.X, K42.X, K43.X, K44.X; Death 40001, 40002; ICD10 I21.X, I22.X, I23.X, I24.X, I25.1, I25.2, I25.3, I25.5, I25.6, I25.6, I25.8, I25.9

**Supplemental Table 1.** Genetic instruments used for SBP for analyses of total CHD

| SNP | CHR | BP | ALLELE1 | ALLELE0 | A1FREQ | BETA | SE | P | phenotype |
| --- | --- | --- | --- | --- | --- | --- | --- | --- | --- |
| rs1623474 | 10 | 18471794 | C | T | 0.669691 | -0.28072 | 0.104556 | 0.0073 | Age ≤55 |
| rs12258967 | 10 | 18727959 | C | G | 0.700794 | 0.730215 | 0.107298 | 1.00E-11 | Age ≤55 |
| rs57946343 | 10 | 63499951 | T | C | 0.848709 | 0.627585 | 0.136873 | 4.50E-06 | Age ≤55 |
| rs10128354 | 10 | 75470045 | T | C | 0.412354 | 0.156039 | 0.099316 | 0.12 | Age ≤55 |
| rs10882398 | 10 | 95892788 | T | A | 0.564517 | -0.52183 | 0.099195 | 1.40E-07 | Age ≤55 |
| rs1006545 | 10 | 1.03E+08 | G | T | 0.111871 | -0.59941 | 0.15551 | 0.00012 | Age ≤55 |
| rs11191551 | 10 | 1.05E+08 | A | G | 0.922905 | 1.03825 | 0.183479 | 1.50E-08 | Age ≤55 |
| rs1801253 | 10 | 1.16E+08 | G | C | 0.265507 | -0.50277 | 0.111424 | 6.40E-06 | Age ≤55 |
| rs11592107 | 10 | 1.23E+08 | G | A | 0.689801 | -0.48891 | 0.106199 | 4.20E-06 | Age ≤55 |
| rs592373 | 11 | 1890990 | G | A | 0.371118 | -0.5277 | 0.101541 | 2.00E-07 | Age ≤55 |
| rs2218305 | 11 | 10247896 | G | C | 0.505986 | -0.18565 | 0.098225 | 0.059 | Age ≤55 |
| rs1966698 | 11 | 16306267 | C | T | 0.206833 | 0.4294 | 0.121096 | 0.00039 | Age ≤55 |
| rs416258 | 11 | 16911636 | G | C | 0.695198 | -0.28635 | 0.10631 | 0.0071 | Age ≤55 |
| rs7107356 | 11 | 47676170 | A | G | 0.491667 | -0.32825 | 0.098062 | 0.00082 | Age ≤55 |
| rs10750766 | 11 | 65473798 | C | A | 0.28985 | -0.28516 | 0.108026 | 0.0083 | Age ≤55 |
| rs139437879 | 11 | 67904333 | G | A | 0.984524 | 0.027015 | 0.404875 | 0.95 | Age ≤55 |
| rs58941166 | 11 | 72083441 | G | A | 0.933818 | -0.21406 | 0.19728 | 0.28 | Age ≤55 |
| rs604723 | 11 | 1.01E+08 | T | C | 0.273457 | -0.84583 | 0.110395 | 1.80E-14 | Age ≤55 |
| rs2072927 | 1 | 1684800 | G | A | 0.499677 | 0.40927 | 0.097896 | 2.90E-05 | Age ≤55 |
| rs488834 | 1 | 10767902 | C | T | 0.232922 | 0.225894 | 0.115364 | 0.05 | Age ≤55 |
| rs6669371 | 1 | 11882142 | T | G | 0.836431 | 0.864351 | 0.132434 | 6.70E-11 | Age ≤55 |
| rs75461554 | 1 | 15810172 | C | T | 0.802543 | 0.158556 | 0.122896 | 0.2 | Age ≤55 |
| rs4908348 | 1 | 28706949 | T | G | 0.693389 | 0.307335 | 0.106447 | 0.0039 | Age ≤55 |
| rs1408945 | 1 | 42364877 | G | T | 0.571901 | 0.292467 | 0.098851 | 0.0031 | Age ≤55 |
| rs12132894 | 1 | 89138474 | A | T | 0.585443 | 0.076856 | 0.099878 | 0.44 | Age ≤55 |
| rs10776752 | 1 | 1.13E+08 | G | T | 0.926731 | -0.65878 | 0.188042 | 0.00046 | Age ≤55 |
| rs10914117 | 1 | 1.81E+08 | T | C | 0.59298 | 0.140409 | 0.099594 | 0.16 | Age ≤55 |
| rs68085857 | 1 | 2.18E+08 | C | T | 0.76692 | -0.09292 | 0.11537 | 0.42 | Age ≤55 |
| rs1745417 | 1 | 2.28E+08 | C | T | 0.487814 | -0.35155 | 0.098019 | 0.00034 | Age ≤55 |
| rs699 | 1 | 2.31E+08 | A | G | 0.59553 | -0.31239 | 0.099777 | 0.0017 | Age ≤55 |
| rs3213751 | 12 | 432666 | G | C | 0.74092 | -0.27914 | 0.111598 | 0.012 | Age ≤55 |
| rs11055034 | 12 | 12890626 | C | A | 0.717939 | 0.231099 | 0.109075 | 0.034 | Age ≤55 |
| rs1010064 | 12 | 20000315 | A | C | 0.813921 | 0.235355 | 0.125825 | 0.061 | Age ≤55 |
| rs73075659 | 12 | 20373541 | A | G | 0.654266 | 0.332079 | 0.103217 | 0.0013 | Age ≤55 |
| rs150857355 | 12 | 49209340 | G | C | 0.978167 | -1.09409 | 0.342207 | 0.0014 | Age ≤55 |
| rs7967954 | 12 | 50673484 | G | A | 0.420704 | 0.271514 | 0.099198 | 0.0062 | Age ≤55 |
| rs736825 | 12 | 54417576 | C | G | 0.636078 | 0.334913 | 0.10219 | 0.001 | Age ≤55 |
| rs4143175 | 12 | 67782397 | T | C | 0.240668 | 0.222706 | 0.115543 | 0.054 | Age ≤55 |
| rs17249754 | 12 | 90060586 | G | A | 0.828249 | 0.710608 | 0.130092 | 4.70E-08 | Age ≤55 |
| rs7310615 | 12 | 1.12E+08 | C | G | 0.479486 | 0.605458 | 0.098679 | 8.50E-10 | Age ≤55 |
| rs35443 | 12 | 1.16E+08 | G | C | 0.622041 | 0.438145 | 0.100546 | 1.30E-05 | Age ≤55 |
| rs6490021 | 12 | 1.16E+08 | A | C | 0.384401 | -0.24819 | 0.100616 | 0.014 | Age ≤55 |
| rs9508495 | 13 | 30146201 | C | T | 0.236602 | 0.207256 | 0.115166 | 0.072 | Age ≤55 |
| rs4415898 | 13 | 41895177 | T | A | 0.978202 | 0.065214 | 0.378418 | 0.86 | Age ≤55 |
| rs4415898 | 13 | 41895177 | T | G | 0.166847 | -0.06069 | 0.131433 | 0.64 | Age ≤55 |
| rs8904 | 14 | 35871217 | G | A | 0.635884 | -0.3256 | 0.101709 | 0.0014 | Age ≤55 |
| rs28365937 | 14 | 53418233 | C | A | 0.695176 | -0.34972 | 0.106735 | 0.0011 | Age ≤55 |
| rs12591513 | 15 | 75102714 | G | A | 0.331153 | 0.597794 | 0.104164 | 9.50E-09 | Age ≤55 |
| rs2627316 | 15 | 81042816 | A | G | 0.531224 | -0.20635 | 0.098422 | 0.036 | Age ≤55 |
| rs4932373 | 15 | 91429287 | A | C | 0.673407 | -0.64049 | 0.104791 | 9.80E-10 | Age ≤55 |
| rs10500326 | 16 | 4918326 | G | T | 0.764944 | 0.179437 | 0.115666 | 0.12 | Age ≤55 |
| rs4293393 | 16 | 20364588 | A | G | 0.817069 | 0.278778 | 0.126822 | 0.028 | Age ≤55 |
| rs4888408 | 16 | 75432824 | G | A | 0.403395 | -0.49402 | 0.099697 | 7.20E-07 | Age ≤55 |
| rs9806885 | 16 | 81556909 | A | G | 0.889773 | 0.257467 | 0.156115 | 0.099 | Age ≤55 |
| rs908951 | 16 | 89697625 | C | T | 0.57063 | 0.268297 | 0.099586 | 0.0071 | Age ≤55 |
| rs222857 | 17 | 7164563 | C | T | 0.433957 | -0.08744 | 0.098865 | 0.38 | Age ≤55 |
| rs7213273 | 17 | 43155914 | G | A | 0.347167 | 0.493133 | 0.102882 | 1.60E-06 | Age ≤55 |
| rs17608766 | 17 | 45013271 | T | C | 0.853368 | -0.5497 | 0.138362 | 7.10E-05 | Age ≤55 |
| rs7406910 | 17 | 46688256 | T | C | 0.089217 | -0.43832 | 0.171718 | 0.011 | Age ≤55 |
| rs2671661 | 17 | 47528562 | T | C | 0.271273 | -0.2374 | 0.11184 | 0.034 | Age ≤55 |
| rs1000423 | 17 | 59475642 | C | T | 0.265467 | -0.23781 | 0.111391 | 0.033 | Age ≤55 |
| rs77946446 | 17 | 75317932 | C | T | 0.734497 | 0.250473 | 0.111641 | 0.025 | Age ≤55 |
| rs12955644 | 18 | 771047 | C | G | 0.815914 | 0.474484 | 0.127021 | 0.00019 | Age ≤55 |
| rs56407827 | 18 | 42179819 | C | T | 0.730226 | -0.40929 | 0.110993 | 0.00023 | Age ≤55 |
| rs76978865 | 19 | 2175799 | G | A | 0.960305 | -1.01775 | 0.251219 | 5.10E-05 | Age ≤55 |
| rs10427021 | 19 | 7259346 | T | G | 0.889696 | 0.76237 | 0.172633 | 1.00E-05 | Age ≤55 |
| rs79709447 | 19 | 12187621 | T | C | 0.987166 | -0.05861 | 0.445263 | 0.9 | Age ≤55 |
| rs1327235 | 20 | 10969030 | A | G | 0.524378 | -0.47778 | 0.098293 | 1.20E-06 | Age ≤55 |
| rs17812022 | 20 | 19007099 | C | T | 0.905616 | -0.11256 | 0.167716 | 0.5 | Age ≤55 |
| rs6102506 | 20 | 40254735 | C | T | 0.650523 | 0.140212 | 0.103783 | 0.18 | Age ≤55 |
| rs6026744 | 20 | 57742388 | A | T | 0.880547 | -0.39853 | 0.152404 | 0.0089 | Age ≤55 |
| rs1275988 | 2 | 26914364 | C | T | 0.384527 | 0.389543 | 0.100867 | 0.00011 | Age ≤55 |
| rs35441226 | 2 | 43191137 | C | T | 0.817108 | 0.368832 | 0.127498 | 0.0038 | Age ≤55 |
| rs56308618 | 21 | 44757792 | G | A | 0.708939 | -0.43315 | 0.108399 | 6.40E-05 | Age ≤55 |
| rs58117425 | 2 | 1.46E+08 | G | A | 0.767118 | -0.21877 | 0.115895 | 0.059 | Age ≤55 |
| rs73029563 | 2 | 1.65E+08 | C | G | 0.458379 | -0.49338 | 0.098334 | 5.20E-07 | Age ≤55 |
| rs10205122 | 2 | 1.81E+08 | A | G | 0.71799 | 0.103727 | 0.109316 | 0.34 | Age ≤55 |
| rs7592578 | 2 | 1.91E+08 | T | G | 0.185283 | -0.29265 | 0.126483 | 0.021 | Age ≤55 |
| rs3845811 | 2 | 2.09E+08 | C | G | 0.566257 | -0.21522 | 0.099079 | 0.03 | Age ≤55 |
| rs2161967 | 2 | 2.19E+08 | T | G | 0.424056 | 0.336741 | 0.099634 | 0.00073 | Age ≤55 |
| rs2594992 | 3 | 11360997 | A | C | 0.597373 | -0.1408 | 0.099487 | 0.16 | Age ≤55 |
| rs34991912 | 3 | 14926351 | T | C | 0.416156 | 0.372765 | 0.099565 | 0.00018 | Age ≤55 |
| rs2643826 | 3 | 27562988 | C | T | 0.550659 | -0.46065 | 0.09871 | 3.10E-06 | Age ≤55 |
| rs6771917 | 3 | 48108442 | T | C | 0.244323 | -0.47504 | 0.114107 | 3.10E-05 | Age ≤55 |
| rs3772219 | 3 | 56771251 | A | C | 0.674705 | 0.375111 | 0.104517 | 0.00033 | Age ≤55 |
| rs73872710 | 3 | 1.41E+08 | A | T | 0.924455 | 0.02842 | 0.185404 | 0.88 | Age ≤55 |
| rs1597467 | 3 | 1.5E+08 | C | A | 0.905624 | -0.30621 | 0.167982 | 0.068 | Age ≤55 |
| rs11715377 | 3 | 1.69E+08 | A | T | 0.906134 | 0.704435 | 0.16871 | 3.00E-05 | Age ≤55 |
| rs1528293 | 3 | 1.69E+08 | A | T | 0.489985 | 0.424766 | 0.097517 | 1.30E-05 | Age ≤55 |
| rs16852391 | 4 | 40604309 | C | G | 0.893366 | 0.009627 | 0.158897 | 0.95 | Age ≤55 |
| rs12509595 | 4 | 81182554 | T | C | 0.706597 | -1.18857 | 0.107859 | 3.10E-28 | Age ≤55 |
| rs17010957 | 4 | 86719165 | T | C | 0.851854 | -0.6496 | 0.139545 | 3.20E-06 | Age ≤55 |
| rs13135092 | 4 | 1.03E+08 | A | G | 0.916262 | 0.293921 | 0.17758 | 0.098 | Age ≤55 |
| rs6820343 | 4 | 1.56E+08 | C | T | 0.466865 | 0.375474 | 0.098568 | 0.00014 | Age ≤55 |
| rs72689147 | 4 | 1.57E+08 | G | T | 0.817263 | 0.467073 | 0.126722 | 0.00023 | Age ≤55 |
| rs10069690 | 5 | 1279790 | C | T | 0.743925 | -0.30672 | 0.111811 | 0.0061 | Age ≤55 |
| rs7733331 | 5 | 32828846 | T | C | 0.401511 | -0.77805 | 0.099869 | 6.70E-15 | Age ≤55 |
| rs2112453 | 5 | 1.12E+08 | G | A | 0.265577 | 0.214068 | 0.110645 | 0.053 | Age ≤55 |
| rs4487481 | 5 | 1.14E+08 | T | C | 0.481266 | 0.186863 | 0.097995 | 0.057 | Age ≤55 |
| rs6890251 | 5 | 1.22E+08 | C | T | 0.848833 | -0.6084 | 0.136826 | 8.70E-06 | Age ≤55 |
| rs7703751 | 5 | 1.23E+08 | A | T | 0.741053 | 0.249058 | 0.111732 | 0.026 | Age ≤55 |
| rs6892983 | 5 | 1.28E+08 | C | A | 0.590824 | -0.22868 | 0.099555 | 0.022 | Age ≤55 |
| rs7737361 | 5 | 1.48E+08 | G | A | 0.804764 | 0.339871 | 0.126168 | 0.0071 | Age ≤55 |
| rs1650581 | 5 | 1.58E+08 | C | G | 0.739321 | -0.30603 | 0.111843 | 0.0062 | Age ≤55 |
| rs12187017 | 5 | 1.58E+08 | G | A | 0.638999 | 0.236289 | 0.10195 | 0.02 | Age ≤55 |
| rs198851 | 6 | 26104632 | T | G | 0.149589 | 0.437516 | 0.137187 | 0.0014 | Age ≤55 |
| rs1265157 | 6 | 31142265 | C | G | 0.648304 | 0.077549 | 0.102657 | 0.45 | Age ≤55 |
| rs2856448 | 6 | 32014575 | A | G | 0.535634 | -0.42427 | 0.098226 | 1.60E-05 | Age ≤55 |
| rs1214761 | 6 | 43354431 | A | G | 0.323561 | 0.280923 | 0.104808 | 0.0074 | Age ≤55 |
| rs211159 | 6 | 97023026 | G | A | 0.658575 | -0.15725 | 0.103501 | 0.13 | Age ≤55 |
| rs4897160 | 6 | 1.26E+08 | G | A | 0.524043 | -0.1539 | 0.09801 | 0.12 | Age ≤55 |
| rs9375459 | 6 | 1.27E+08 | C | T | 0.563472 | -0.49324 | 0.099188 | 6.60E-07 | Age ≤55 |
| rs62434129 | 6 | 1.51E+08 | A | T | 0.928902 | 0.797582 | 0.190592 | 2.90E-05 | Age ≤55 |
| rs2969036 | 7 | 2534901 | T | G | 0.310141 | 0.473903 | 0.105973 | 7.80E-06 | Age ≤55 |
| rs3735533 | 7 | 27245893 | T | C | 0.072747 | -0.56033 | 0.188589 | 0.003 | Age ≤55 |
| rs6961048 | 7 | 27328187 | C | G | 0.897386 | -0.57751 | 0.161491 | 0.00035 | Age ≤55 |
| rs2392929 | 7 | 1.06E+08 | T | G | 0.802054 | -0.60044 | 0.122562 | 9.60E-07 | Age ≤55 |
| rs3807306 | 7 | 1.29E+08 | G | T | 0.507392 | 0.050308 | 0.098095 | 0.61 | Age ≤55 |
| rs3918226 | 7 | 1.51E+08 | C | T | 0.918315 | -0.56644 | 0.182402 | 0.0019 | Age ≤55 |
| rs1991651 | 8 | 10706411 | C | G | 0.381211 | 0.479043 | 0.101067 | 2.10E-06 | Age ≤55 |
| rs1352026 | 8 | 25895899 | C | T | 0.745691 | 0.404501 | 0.112487 | 0.00032 | Age ≤55 |
| rs830444 | 8 | 76954113 | G | T | 0.397783 | -0.36594 | 0.10035 | 0.00027 | Age ≤55 |
| rs35783704 | 8 | 1.06E+08 | G | A | 0.899238 | 0.462001 | 0.164093 | 0.0049 | Age ≤55 |
| rs62521878 | 8 | 1.42E+08 | C | T | 0.703742 | -0.18072 | 0.106727 | 0.09 | Age ≤55 |
| rs72762681 | 9 | 1.13E+08 | A | T | 0.849964 | -0.12247 | 0.137269 | 0.37 | Age ≤55 |
| rs1623474 | 10 | 18471794 | C | T | 0.66865 | -0.43172 | 0.176571 | 0.014 | Age 56-60 |
| rs12258967 | 10 | 18727959 | C | G | 0.698188 | 0.802471 | 0.179772 | 8.10E-06 | Age 56-60 |
| rs57946343 | 10 | 63499951 | T | C | 0.847303 | 1.1179 | 0.229291 | 1.10E-06 | Age 56-60 |
| rs10128354 | 10 | 75470045 | T | C | 0.412175 | 0.433537 | 0.167259 | 0.0095 | Age 56-60 |
| rs10882398 | 10 | 95892788 | T | A | 0.565278 | -0.30623 | 0.167684 | 0.068 | Age 56-60 |
| rs1006545 | 10 | 1.03E+08 | G | T | 0.112173 | -0.94289 | 0.262184 | 0.00032 | Age 56-60 |
| rs11191551 | 10 | 1.05E+08 | A | G | 0.924016 | 1.44972 | 0.312229 | 3.40E-06 | Age 56-60 |
| rs1801253 | 10 | 1.16E+08 | G | C | 0.265628 | -0.92647 | 0.187498 | 7.80E-07 | Age 56-60 |
| rs11592107 | 10 | 1.23E+08 | G | A | 0.691616 | -0.08164 | 0.178533 | 0.65 | Age 56-60 |
| rs592373 | 11 | 1890990 | G | A | 0.367465 | -0.51749 | 0.171187 | 0.0025 | Age 56-60 |
| rs2218305 | 11 | 10247896 | G | C | 0.507334 | -0.54588 | 0.164419 | 9.00E-04 | Age 56-60 |
| rs1966698 | 11 | 16306267 | C | T | 0.20669 | 0.485574 | 0.203005 | 0.017 | Age 56-60 |
| rs416258 | 11 | 16911636 | G | C | 0.69183 | -0.26652 | 0.178554 | 0.14 | Age 56-60 |
| rs7107356 | 11 | 47676170 | A | G | 0.490041 | -0.61608 | 0.165 | 0.00019 | Age 56-60 |
| rs10750766 | 11 | 65473798 | C | A | 0.295628 | -0.47931 | 0.181375 | 0.0082 | Age 56-60 |
| rs139437879 | 11 | 67904333 | G | A | 0.984334 | 0.803043 | 0.679763 | 0.24 | Age 56-60 |
| rs58941166 | 11 | 72083441 | G | A | 0.933662 | -0.38334 | 0.331675 | 0.25 | Age 56-60 |
| rs604723 | 11 | 1.01E+08 | T | C | 0.274845 | -0.55302 | 0.185999 | 0.0029 | Age 56-60 |
| rs2072927 | 1 | 1684800 | G | A | 0.502227 | 0.283023 | 0.164481 | 0.085 | Age 56-60 |
| rs488834 | 1 | 10767902 | C | T | 0.230276 | 0.531581 | 0.195485 | 0.0065 | Age 56-60 |
| rs6669371 | 1 | 11882142 | T | G | 0.836408 | 0.676107 | 0.223137 | 0.0024 | Age 56-60 |
| rs75461554 | 1 | 15810172 | C | T | 0.80436 | 0.481403 | 0.20771 | 0.02 | Age 56-60 |
| rs4908348 | 1 | 28706949 | T | G | 0.697671 | 0.054911 | 0.180069 | 0.76 | Age 56-60 |
| rs1408945 | 1 | 42364877 | G | T | 0.577517 | -0.08002 | 0.167096 | 0.63 | Age 56-60 |
| rs12132894 | 1 | 89138474 | A | T | 0.586489 | 0.400547 | 0.168242 | 0.017 | Age 56-60 |
| rs10776752 | 1 | 1.13E+08 | G | T | 0.92526 | -1.13384 | 0.313097 | 0.00029 | Age 56-60 |
| rs10914117 | 1 | 1.81E+08 | T | C | 0.594174 | 0.213228 | 0.168648 | 0.21 | Age 56-60 |
| rs68085857 | 1 | 2.18E+08 | C | T | 0.769119 | -0.00939 | 0.194898 | 0.96 | Age 56-60 |
| rs1745417 | 1 | 2.28E+08 | C | T | 0.485647 | -0.45705 | 0.165191 | 0.0057 | Age 56-60 |
| rs699 | 1 | 2.31E+08 | A | G | 0.595247 | -0.26017 | 0.16817 | 0.12 | Age 56-60 |
| rs3213751 | 12 | 432666 | G | C | 0.740431 | -0.06151 | 0.188672 | 0.74 | Age 56-60 |
| rs11055034 | 12 | 12890626 | C | A | 0.721843 | 0.212638 | 0.184188 | 0.25 | Age 56-60 |
| rs1010064 | 12 | 20000315 | A | C | 0.815729 | 0.547883 | 0.213075 | 0.01 | Age 56-60 |
| rs73075659 | 12 | 20373541 | A | G | 0.650991 | 0.155583 | 0.174308 | 0.37 | Age 56-60 |
| rs150857355 | 12 | 49209340 | G | C | 0.978751 | -1.396 | 0.581446 | 0.016 | Age 56-60 |
| rs7967954 | 12 | 50673484 | G | A | 0.421597 | 0.443286 | 0.16657 | 0.0078 | Age 56-60 |
| rs736825 | 12 | 54417576 | C | G | 0.639873 | 0.298084 | 0.17253 | 0.084 | Age 56-60 |
| rs4143175 | 12 | 67782397 | T | C | 0.240731 | 0.343093 | 0.193733 | 0.077 | Age 56-60 |
| rs17249754 | 12 | 90060586 | G | A | 0.827797 | 0.648585 | 0.218462 | 0.003 | Age 56-60 |
| rs7310615 | 12 | 1.12E+08 | C | G | 0.482896 | 0.381605 | 0.165893 | 0.021 | Age 56-60 |
| rs35443 | 12 | 1.16E+08 | G | C | 0.618035 | 0.57891 | 0.169854 | 0.00065 | Age 56-60 |
| rs6490021 | 12 | 1.16E+08 | A | C | 0.380536 | -0.46895 | 0.170096 | 0.0058 | Age 56-60 |
| rs9508495 | 13 | 30146201 | C | T | 0.238033 | 0.59564 | 0.194716 | 0.0022 | Age 56-60 |
| rs4415898 | 13 | 41895177 | T | A | 0.978516 | -0.84377 | 0.640659 | 0.19 | Age 56-60 |
| rs4415898 | 13 | 41895177 | T | G | 0.170731 | -0.38577 | 0.219573 | 0.079 | Age 56-60 |
| rs8904 | 14 | 35871217 | G | A | 0.638913 | -0.78789 | 0.171532 | 4.40E-06 | Age 56-60 |
| rs28365937 | 14 | 53418233 | C | A | 0.696304 | -0.07222 | 0.18041 | 0.69 | Age 56-60 |
| rs12591513 | 15 | 75102714 | G | A | 0.33325 | 0.466547 | 0.174027 | 0.0073 | Age 56-60 |
| rs2627316 | 15 | 81042816 | A | G | 0.526823 | -0.48678 | 0.16641 | 0.0034 | Age 56-60 |
| rs4932373 | 15 | 91429287 | A | C | 0.672038 | -1.07425 | 0.174699 | 7.80E-10 | Age 56-60 |
| rs10500326 | 16 | 4918326 | G | T | 0.759452 | 0.29709 | 0.193466 | 0.12 | Age 56-60 |
| rs4293393 | 16 | 20364588 | A | G | 0.816275 | 0.40658 | 0.212074 | 0.055 | Age 56-60 |
| rs4888408 | 16 | 75432824 | G | A | 0.404602 | -0.39923 | 0.16837 | 0.018 | Age 56-60 |
| rs9806885 | 16 | 81556909 | A | G | 0.887091 | 0.145229 | 0.261454 | 0.58 | Age 56-60 |
| rs908951 | 16 | 89697625 | C | T | 0.57123 | 0.461698 | 0.167824 | 0.0059 | Age 56-60 |
| rs222857 | 17 | 7164563 | C | T | 0.43062 | -0.20288 | 0.166313 | 0.22 | Age 56-60 |
| rs7213273 | 17 | 43155914 | G | A | 0.347578 | 0.666177 | 0.173283 | 0.00012 | Age 56-60 |
| rs17608766 | 17 | 45013271 | T | C | 0.857093 | -0.62846 | 0.235776 | 0.0077 | Age 56-60 |
| rs7406910 | 17 | 46688256 | T | C | 0.086166 | -0.08444 | 0.292736 | 0.77 | Age 56-60 |
| rs2671661 | 17 | 47528562 | T | C | 0.272814 | -0.36185 | 0.187854 | 0.054 | Age 56-60 |
| rs1000423 | 17 | 59475642 | C | T | 0.267633 | -0.60001 | 0.186066 | 0.0013 | Age 56-60 |
| rs77946446 | 17 | 75317932 | C | T | 0.735457 | 0.207754 | 0.188323 | 0.27 | Age 56-60 |
| rs12955644 | 18 | 771047 | C | G | 0.815528 | 0.443612 | 0.215018 | 0.039 | Age 56-60 |
| rs56407827 | 18 | 42179819 | C | T | 0.733042 | -0.33533 | 0.186689 | 0.072 | Age 56-60 |
| rs76978865 | 19 | 2175799 | G | A | 0.959238 | -0.79499 | 0.418818 | 0.058 | Age 56-60 |
| rs10427021 | 19 | 7259346 | T | G | 0.890716 | 1.35838 | 0.292514 | 3.40E-06 | Age 56-60 |
| rs79709447 | 19 | 12187621 | T | C | 0.988097 | 0.283714 | 0.777172 | 0.72 | Age 56-60 |
| rs1327235 | 20 | 10969030 | A | G | 0.526355 | -0.37096 | 0.165376 | 0.025 | Age 56-60 |
| rs17812022 | 20 | 19007099 | C | T | 0.906383 | 0.179515 | 0.284915 | 0.53 | Age 56-60 |
| rs6102506 | 20 | 40254735 | C | T | 0.651539 | -0.22722 | 0.174589 | 0.19 | Age 56-60 |
| rs6026744 | 20 | 57742388 | A | T | 0.881122 | -1.20475 | 0.256859 | 2.70E-06 | Age 56-60 |
| rs1275988 | 2 | 26914364 | C | T | 0.383528 | 0.943558 | 0.170202 | 3.00E-08 | Age 56-60 |
| rs35441226 | 2 | 43191137 | C | T | 0.817776 | 0.272842 | 0.213859 | 0.2 | Age 56-60 |
| rs56308618 | 21 | 44757792 | G | A | 0.709159 | -0.38996 | 0.182403 | 0.033 | Age 56-60 |
| rs58117425 | 2 | 1.46E+08 | G | A | 0.760793 | -0.46572 | 0.194308 | 0.017 | Age 56-60 |
| rs73029563 | 2 | 1.65E+08 | C | G | 0.455172 | -0.27029 | 0.166167 | 0.1 | Age 56-60 |
| rs10205122 | 2 | 1.81E+08 | A | G | 0.716488 | 0.511817 | 0.183473 | 0.0053 | Age 56-60 |
| rs7592578 | 2 | 1.91E+08 | T | G | 0.185318 | -0.5879 | 0.213238 | 0.0058 | Age 56-60 |
| rs3845811 | 2 | 2.09E+08 | C | G | 0.565261 | -0.3154 | 0.165342 | 0.056 | Age 56-60 |
| rs2161967 | 2 | 2.19E+08 | T | G | 0.424929 | 0.448488 | 0.167161 | 0.0073 | Age 56-60 |
| rs2594992 | 3 | 11360997 | A | C | 0.59656 | -0.27057 | 0.168396 | 0.11 | Age 56-60 |
| rs34991912 | 3 | 14926351 | T | C | 0.415514 | 0.400354 | 0.167655 | 0.017 | Age 56-60 |
| rs2643826 | 3 | 27562988 | C | T | 0.552259 | -0.68771 | 0.166894 | 3.80E-05 | Age 56-60 |
| rs6771917 | 3 | 48108442 | T | C | 0.24354 | -0.31983 | 0.192608 | 0.097 | Age 56-60 |
| rs3772219 | 3 | 56771251 | A | C | 0.674884 | 0.42928 | 0.176404 | 0.015 | Age 56-60 |
| rs73872710 | 3 | 1.41E+08 | A | T | 0.923407 | 0.08992 | 0.312805 | 0.77 | Age 56-60 |
| rs1597467 | 3 | 1.5E+08 | C | A | 0.906383 | -0.68258 | 0.280653 | 0.015 | Age 56-60 |
| rs11715377 | 3 | 1.69E+08 | A | T | 0.904729 | 0.272907 | 0.281052 | 0.33 | Age 56-60 |
| rs1528293 | 3 | 1.69E+08 | A | T | 0.488932 | 0.425966 | 0.165597 | 0.01 | Age 56-60 |
| rs16852391 | 4 | 40604309 | C | G | 0.895601 | -0.00867 | 0.268847 | 0.97 | Age 56-60 |
| rs12509595 | 4 | 81182554 | T | C | 0.708466 | -1.00414 | 0.181557 | 3.20E-08 | Age 56-60 |
| rs17010957 | 4 | 86719165 | T | C | 0.85147 | -0.09845 | 0.234446 | 0.67 | Age 56-60 |
| rs13135092 | 4 | 1.03E+08 | A | G | 0.916829 | 1.09788 | 0.300148 | 0.00025 | Age 56-60 |
| rs6820343 | 4 | 1.56E+08 | C | T | 0.463394 | 0.394941 | 0.164943 | 0.017 | Age 56-60 |
| rs72689147 | 4 | 1.57E+08 | G | T | 0.818646 | 0.31275 | 0.214309 | 0.14 | Age 56-60 |
| rs10069690 | 5 | 1279790 | C | T | 0.74078 | -0.46117 | 0.187988 | 0.014 | Age 56-60 |
| rs7733331 | 5 | 32828846 | T | C | 0.402121 | -0.94394 | 0.168435 | 2.10E-08 | Age 56-60 |
| rs2112453 | 5 | 1.12E+08 | G | A | 0.265737 | 0.270664 | 0.186943 | 0.15 | Age 56-60 |
| rs4487481 | 5 | 1.14E+08 | T | C | 0.483604 | 0.229859 | 0.164865 | 0.16 | Age 56-60 |
| rs6890251 | 5 | 1.22E+08 | C | T | 0.849878 | -0.34627 | 0.231432 | 0.13 | Age 56-60 |
| rs7703751 | 5 | 1.23E+08 | A | T | 0.740623 | 0.536149 | 0.187507 | 0.0042 | Age 56-60 |
| rs6892983 | 5 | 1.28E+08 | C | A | 0.591613 | -0.41573 | 0.167889 | 0.013 | Age 56-60 |
| rs7737361 | 5 | 1.48E+08 | G | A | 0.80745 | 0.550612 | 0.213219 | 0.0098 | Age 56-60 |
| rs1650581 | 5 | 1.58E+08 | C | G | 0.736859 | -0.52492 | 0.18861 | 0.0054 | Age 56-60 |
| rs12187017 | 5 | 1.58E+08 | G | A | 0.638445 | 0.399381 | 0.171818 | 0.02 | Age 56-60 |
| rs198851 | 6 | 26104632 | T | G | 0.153366 | 0.159784 | 0.229961 | 0.49 | Age 56-60 |
| rs1265157 | 6 | 31142265 | C | G | 0.651282 | 0.108893 | 0.172532 | 0.53 | Age 56-60 |
| rs2856448 | 6 | 32014575 | A | G | 0.532597 | -0.23168 | 0.166734 | 0.16 | Age 56-60 |
| rs1214761 | 6 | 43354431 | A | G | 0.322909 | 0.295418 | 0.177913 | 0.097 | Age 56-60 |
| rs211159 | 6 | 97023026 | G | A | 0.657023 | -0.47125 | 0.174891 | 0.007 | Age 56-60 |
| rs4897160 | 6 | 1.26E+08 | G | A | 0.522526 | -0.21714 | 0.164357 | 0.19 | Age 56-60 |
| rs9375459 | 6 | 1.27E+08 | C | T | 0.562512 | -0.66419 | 0.166713 | 6.80E-05 | Age 56-60 |
| rs62434129 | 6 | 1.51E+08 | A | T | 0.927864 | 1.06168 | 0.320779 | 0.00093 | Age 56-60 |
| rs2969036 | 7 | 2534901 | T | G | 0.309895 | 0.086199 | 0.178854 | 0.63 | Age 56-60 |
| rs3735533 | 7 | 27245893 | T | C | 0.076198 | -1.17881 | 0.30976 | 0.00014 | Age 56-60 |
| rs6961048 | 7 | 27328187 | C | G | 0.900267 | -0.33766 | 0.274828 | 0.22 | Age 56-60 |
| rs2392929 | 7 | 1.06E+08 | T | G | 0.802515 | -0.91211 | 0.207168 | 1.10E-05 | Age 56-60 |
| rs3807306 | 7 | 1.29E+08 | G | T | 0.506273 | 0.074607 | 0.164461 | 0.65 | Age 56-60 |
| rs3918226 | 7 | 1.51E+08 | C | T | 0.918311 | -1.0722 | 0.306684 | 0.00047 | Age 56-60 |
| rs1991651 | 8 | 10706411 | C | G | 0.381336 | 0.433005 | 0.171118 | 0.011 | Age 56-60 |
| rs1352026 | 8 | 25895899 | C | T | 0.74784 | 0.343854 | 0.189664 | 0.07 | Age 56-60 |
| rs830444 | 8 | 76954113 | G | T | 0.39952 | -0.50431 | 0.168596 | 0.0028 | Age 56-60 |
| rs35783704 | 8 | 1.06E+08 | G | A | 0.899722 | 0.599062 | 0.277272 | 0.031 | Age 56-60 |
| rs62521878 | 8 | 1.42E+08 | C | T | 0.704881 | -0.33546 | 0.181363 | 0.064 | Age 56-60 |
| rs72762681 | 9 | 1.13E+08 | A | T | 0.84703 | -0.07588 | 0.229297 | 0.74 | Age 56-60 |
| rs1623474 | 10 | 18471794 | C | T | 0.67026 | -0.51701 | 0.165694 | 0.0018 | Age 61-65 |
| rs12258967 | 10 | 18727959 | C | G | 0.699736 | 0.73349 | 0.170812 | 1.80E-05 | Age 61-65 |
| rs57946343 | 10 | 63499951 | T | C | 0.847495 | 1.16121 | 0.217622 | 9.50E-08 | Age 61-65 |
| rs10128354 | 10 | 75470045 | T | C | 0.416419 | 0.362502 | 0.158512 | 0.022 | Age 61-65 |
| rs10882398 | 10 | 95892788 | T | A | 0.56359 | -0.6053 | 0.158274 | 0.00013 | Age 61-65 |
| rs1006545 | 10 | 1.03E+08 | G | T | 0.112763 | -1.01216 | 0.24684 | 4.10E-05 | Age 61-65 |
| rs11191551 | 10 | 1.05E+08 | A | G | 0.924442 | 1.55808 | 0.294935 | 1.30E-07 | Age 61-65 |
| rs1801253 | 10 | 1.16E+08 | G | C | 0.262397 | -0.79329 | 0.179523 | 9.90E-06 | Age 61-65 |
| rs11592107 | 10 | 1.23E+08 | G | A | 0.687226 | -0.33722 | 0.168562 | 0.045 | Age 61-65 |
| rs592373 | 11 | 1890990 | G | A | 0.369606 | -0.81919 | 0.162076 | 4.30E-07 | Age 61-65 |
| rs2218305 | 11 | 10247896 | G | C | 0.506494 | -0.42708 | 0.156752 | 0.0064 | Age 61-65 |
| rs1966698 | 11 | 16306267 | C | T | 0.203841 | 0.787238 | 0.194261 | 5.10E-05 | Age 61-65 |
| rs416258 | 11 | 16911636 | G | C | 0.697336 | -0.24012 | 0.170781 | 0.16 | Age 61-65 |
| rs7107356 | 11 | 47676170 | A | G | 0.496348 | -0.45406 | 0.156523 | 0.0037 | Age 61-65 |
| rs10750766 | 11 | 65473798 | C | A | 0.291637 | -0.56288 | 0.172585 | 0.0011 | Age 61-65 |
| rs139437879 | 11 | 67904333 | G | A | 0.983842 | 0.361335 | 0.63082 | 0.57 | Age 61-65 |
| rs58941166 | 11 | 72083441 | G | A | 0.933188 | -0.14143 | 0.313826 | 0.65 | Age 61-65 |
| rs604723 | 11 | 1.01E+08 | T | C | 0.276328 | -0.62364 | 0.17601 | 4.00E-04 | Age 61-65 |
| rs2072927 | 1 | 1684800 | G | A | 0.501055 | 0.596568 | 0.156355 | 0.00014 | Age 61-65 |
| rs488834 | 1 | 10767902 | C | T | 0.232402 | 0.431994 | 0.184753 | 0.019 | Age 61-65 |
| rs6669371 | 1 | 11882142 | T | G | 0.834861 | 0.898367 | 0.211405 | 2.10E-05 | Age 61-65 |
| rs75461554 | 1 | 15810172 | C | T | 0.804953 | 0.318594 | 0.196457 | 0.1 | Age 61-65 |
| rs4908348 | 1 | 28706949 | T | G | 0.694987 | 0.471049 | 0.169344 | 0.0054 | Age 61-65 |
| rs1408945 | 1 | 42364877 | G | T | 0.573157 | 0.208237 | 0.158286 | 0.19 | Age 61-65 |
| rs12132894 | 1 | 89138474 | A | T | 0.579054 | 0.300424 | 0.158173 | 0.058 | Age 61-65 |
| rs10776752 | 1 | 1.13E+08 | G | T | 0.927242 | -1.02783 | 0.301288 | 0.00065 | Age 61-65 |
| rs10914117 | 1 | 1.81E+08 | T | C | 0.593218 | 0.348688 | 0.159088 | 0.028 | Age 61-65 |
| rs68085857 | 1 | 2.18E+08 | C | T | 0.771603 | -0.37903 | 0.185802 | 0.041 | Age 61-65 |
| rs1745417 | 1 | 2.28E+08 | C | T | 0.485707 | -0.37721 | 0.156457 | 0.016 | Age 61-65 |
| rs699 | 1 | 2.31E+08 | A | G | 0.601984 | -0.25397 | 0.159253 | 0.11 | Age 61-65 |
| rs3213751 | 12 | 432666 | G | C | 0.740361 | 0.054193 | 0.178989 | 0.76 | Age 61-65 |
| rs11055034 | 12 | 12890626 | C | A | 0.716443 | 0.426153 | 0.17447 | 0.015 | Age 61-65 |
| rs1010064 | 12 | 20000315 | A | C | 0.8137 | 0.686084 | 0.200904 | 0.00064 | Age 61-65 |
| rs73075659 | 12 | 20373541 | A | G | 0.652348 | 0.452976 | 0.164743 | 0.006 | Age 61-65 |
| rs150857355 | 12 | 49209340 | G | C | 0.977805 | -0.73924 | 0.53813 | 0.17 | Age 61-65 |
| rs7967954 | 12 | 50673484 | G | A | 0.42337 | 0.244174 | 0.158564 | 0.12 | Age 61-65 |
| rs736825 | 12 | 54417576 | C | G | 0.636981 | 0.373297 | 0.163344 | 0.022 | Age 61-65 |
| rs4143175 | 12 | 67782397 | T | C | 0.242844 | 0.289132 | 0.182526 | 0.11 | Age 61-65 |
| rs17249754 | 12 | 90060586 | G | A | 0.826764 | 0.832804 | 0.207379 | 5.90E-05 | Age 61-65 |
| rs7310615 | 12 | 1.12E+08 | C | G | 0.484454 | 0.390801 | 0.157258 | 0.013 | Age 61-65 |
| rs35443 | 12 | 1.16E+08 | G | C | 0.614522 | 0.548931 | 0.160818 | 0.00064 | Age 61-65 |
| rs6490021 | 12 | 1.16E+08 | A | C | 0.381243 | -0.58321 | 0.161722 | 0.00031 | Age 61-65 |
| rs9508495 | 13 | 30146201 | C | T | 0.236653 | 0.080336 | 0.183238 | 0.66 | Age 61-65 |
| rs4415898 | 13 | 41895177 | T | A | 0.977481 | -0.64743 | 0.593699 | 0.28 | Age 61-65 |
| rs4415898 | 13 | 41895177 | T | G | 0.166261 | -0.10522 | 0.21108 | 0.62 | Age 61-65 |
| rs8904 | 14 | 35871217 | G | A | 0.636491 | -0.54714 | 0.16229 | 0.00075 | Age 61-65 |
| rs28365937 | 14 | 53418233 | C | A | 0.695131 | -0.23606 | 0.170292 | 0.17 | Age 61-65 |
| rs12591513 | 15 | 75102714 | G | A | 0.329235 | 0.274295 | 0.166757 | 0.1 | Age 61-65 |
| rs2627316 | 15 | 81042816 | A | G | 0.53101 | -0.53216 | 0.157039 | 7.00E-04 | Age 61-65 |
| rs4932373 | 15 | 91429287 | A | C | 0.676141 | -0.74102 | 0.166478 | 8.50E-06 | Age 61-65 |
| rs10500326 | 16 | 4918326 | G | T | 0.764453 | 0.450785 | 0.184809 | 0.015 | Age 61-65 |
| rs4293393 | 16 | 20364588 | A | G | 0.81767 | 0.198894 | 0.201911 | 0.32 | Age 61-65 |
| rs4888408 | 16 | 75432824 | G | A | 0.402504 | -0.22247 | 0.158728 | 0.16 | Age 61-65 |
| rs9806885 | 16 | 81556909 | A | G | 0.888047 | 0.464456 | 0.247966 | 0.061 | Age 61-65 |
| rs908951 | 16 | 89697625 | C | T | 0.570635 | 0.080381 | 0.159438 | 0.61 | Age 61-65 |
| rs222857 | 17 | 7164563 | C | T | 0.425687 | -0.49824 | 0.158639 | 0.0017 | Age 61-65 |
| rs7213273 | 17 | 43155914 | G | A | 0.346453 | 0.434914 | 0.165245 | 0.0085 | Age 61-65 |
| rs17608766 | 17 | 45013271 | T | C | 0.854001 | -0.80181 | 0.220691 | 0.00028 | Age 61-65 |
| rs7406910 | 17 | 46688256 | T | C | 0.088842 | -0.3467 | 0.273645 | 0.21 | Age 61-65 |
| rs2671661 | 17 | 47528562 | T | C | 0.270627 | -0.25131 | 0.177739 | 0.16 | Age 61-65 |
| rs1000423 | 17 | 59475642 | C | T | 0.265826 | -0.71543 | 0.17775 | 5.70E-05 | Age 61-65 |
| rs77946446 | 17 | 75317932 | C | T | 0.733996 | 0.446745 | 0.178075 | 0.012 | Age 61-65 |
| rs12955644 | 18 | 771047 | C | G | 0.817666 | 0.001083 | 0.204752 | 1 | Age 61-65 |
| rs56407827 | 18 | 42179819 | C | T | 0.734681 | -0.15918 | 0.177594 | 0.37 | Age 61-65 |
| rs76978865 | 19 | 2175799 | G | A | 0.959179 | -1.05392 | 0.395197 | 0.0077 | Age 61-65 |
| rs10427021 | 19 | 7259346 | T | G | 0.889565 | 0.796925 | 0.274684 | 0.0037 | Age 61-65 |
| rs79709447 | 19 | 12187621 | T | C | 0.986851 | 0.391213 | 0.694461 | 0.57 | Age 61-65 |
| rs1327235 | 20 | 10969030 | A | G | 0.522329 | -0.37209 | 0.157015 | 0.018 | Age 61-65 |
| rs17812022 | 20 | 19007099 | C | T | 0.904365 | 0.535459 | 0.266922 | 0.045 | Age 61-65 |
| rs6102506 | 20 | 40254735 | C | T | 0.648978 | 0.408503 | 0.165945 | 0.014 | Age 61-65 |
| rs6026744 | 20 | 57742388 | A | T | 0.881725 | -1.02016 | 0.242538 | 2.60E-05 | Age 61-65 |
| rs1275988 | 2 | 26914364 | C | T | 0.383928 | 0.635444 | 0.160101 | 7.20E-05 | Age 61-65 |
| rs35441226 | 2 | 43191137 | C | T | 0.818221 | 0.173098 | 0.203233 | 0.39 | Age 61-65 |
| rs56308618 | 21 | 44757792 | G | A | 0.70631 | -0.16638 | 0.172566 | 0.33 | Age 61-65 |
| rs58117425 | 2 | 1.46E+08 | G | A | 0.762045 | -0.34615 | 0.183962 | 0.06 | Age 61-65 |
| rs73029563 | 2 | 1.65E+08 | C | G | 0.456433 | -0.54704 | 0.156582 | 0.00048 | Age 61-65 |
| rs10205122 | 2 | 1.81E+08 | A | G | 0.71716 | 0.150112 | 0.175402 | 0.39 | Age 61-65 |
| rs7592578 | 2 | 1.91E+08 | T | G | 0.184056 | 0.058564 | 0.200502 | 0.77 | Age 61-65 |
| rs3845811 | 2 | 2.09E+08 | C | G | 0.565438 | -0.28707 | 0.157583 | 0.068 | Age 61-65 |
| rs2161967 | 2 | 2.19E+08 | T | G | 0.425225 | 0.466112 | 0.158586 | 0.0033 | Age 61-65 |
| rs2594992 | 3 | 11360997 | A | C | 0.596402 | -0.29813 | 0.159298 | 0.061 | Age 61-65 |
| rs34991912 | 3 | 14926351 | T | C | 0.414638 | 0.124745 | 0.159229 | 0.43 | Age 61-65 |
| rs2643826 | 3 | 27562988 | C | T | 0.550342 | -0.72882 | 0.157503 | 3.70E-06 | Age 61-65 |
| rs6771917 | 3 | 48108442 | T | C | 0.243829 | -0.10673 | 0.183458 | 0.56 | Age 61-65 |
| rs3772219 | 3 | 56771251 | A | C | 0.674217 | 0.312183 | 0.166028 | 0.06 | Age 61-65 |
| rs73872710 | 3 | 1.41E+08 | A | T | 0.924052 | -0.62017 | 0.295697 | 0.036 | Age 61-65 |
| rs1597467 | 3 | 1.5E+08 | C | A | 0.908991 | -0.36304 | 0.272882 | 0.18 | Age 61-65 |
| rs11715377 | 3 | 1.69E+08 | A | T | 0.905184 | 0.364793 | 0.265411 | 0.17 | Age 61-65 |
| rs1528293 | 3 | 1.69E+08 | A | T | 0.489379 | 0.257385 | 0.156393 | 0.1 | Age 61-65 |
| rs16852391 | 4 | 40604309 | C | G | 0.89789 | -0.34752 | 0.257855 | 0.18 | Age 61-65 |
| rs12509595 | 4 | 81182554 | T | C | 0.706798 | -0.67181 | 0.171985 | 9.40E-05 | Age 61-65 |
| rs17010957 | 4 | 86719165 | T | C | 0.851549 | -0.3411 | 0.221938 | 0.12 | Age 61-65 |
| rs13135092 | 4 | 1.03E+08 | A | G | 0.914793 | 0.370317 | 0.280686 | 0.19 | Age 61-65 |
| rs6820343 | 4 | 1.56E+08 | C | T | 0.468075 | 0.358062 | 0.156264 | 0.022 | Age 61-65 |
| rs72689147 | 4 | 1.57E+08 | G | T | 0.816062 | 0.196197 | 0.201377 | 0.33 | Age 61-65 |
| rs10069690 | 5 | 1279790 | C | T | 0.742386 | -0.05736 | 0.178334 | 0.75 | Age 61-65 |
| rs7733331 | 5 | 32828846 | T | C | 0.399865 | -1.01697 | 0.160007 | 2.10E-10 | Age 61-65 |
| rs2112453 | 5 | 1.12E+08 | G | A | 0.263871 | 0.280533 | 0.177726 | 0.11 | Age 61-65 |
| rs4487481 | 5 | 1.14E+08 | T | C | 0.48219 | 0.280474 | 0.156792 | 0.074 | Age 61-65 |
| rs6890251 | 5 | 1.22E+08 | C | T | 0.847152 | -0.38736 | 0.217708 | 0.075 | Age 61-65 |
| rs7703751 | 5 | 1.23E+08 | A | T | 0.740664 | 0.611447 | 0.179381 | 0.00065 | Age 61-65 |
| rs6892983 | 5 | 1.28E+08 | C | A | 0.589441 | -0.14478 | 0.158985 | 0.36 | Age 61-65 |
| rs7737361 | 5 | 1.48E+08 | G | A | 0.807232 | 0.472259 | 0.201363 | 0.019 | Age 61-65 |
| rs1650581 | 5 | 1.58E+08 | C | G | 0.738799 | -0.23256 | 0.178994 | 0.19 | Age 61-65 |
| rs12187017 | 5 | 1.58E+08 | G | A | 0.639617 | 0.470728 | 0.163363 | 0.004 | Age 61-65 |
| rs198851 | 6 | 26104632 | T | G | 0.152106 | 0.680541 | 0.217375 | 0.0017 | Age 61-65 |
| rs1265157 | 6 | 31142265 | C | G | 0.653282 | 0.279385 | 0.164421 | 0.089 | Age 61-65 |
| rs2856448 | 6 | 32014575 | A | G | 0.530215 | -0.44341 | 0.156631 | 0.0046 | Age 61-65 |
| rs1214761 | 6 | 43354431 | A | G | 0.320044 | 0.568361 | 0.167446 | 0.00069 | Age 61-65 |
| rs211159 | 6 | 97023026 | G | A | 0.659752 | 0.031917 | 0.164775 | 0.85 | Age 61-65 |
| rs4897160 | 6 | 1.26E+08 | G | A | 0.522345 | -0.03408 | 0.15634 | 0.83 | Age 61-65 |
| rs9375459 | 6 | 1.27E+08 | C | T | 0.562924 | -0.79362 | 0.157636 | 4.80E-07 | Age 61-65 |
| rs62434129 | 6 | 1.51E+08 | A | T | 0.929368 | 0.680463 | 0.305821 | 0.026 | Age 61-65 |
| rs2969036 | 7 | 2534901 | T | G | 0.312913 | 0.428554 | 0.16941 | 0.011 | Age 61-65 |
| rs3735533 | 7 | 27245893 | T | C | 0.071773 | -1.72109 | 0.30241 | 1.30E-08 | Age 61-65 |
| rs6961048 | 7 | 27328187 | C | G | 0.900114 | -0.62434 | 0.25979 | 0.016 | Age 61-65 |
| rs2392929 | 7 | 1.06E+08 | T | G | 0.804297 | -1.24003 | 0.196801 | 3.00E-10 | Age 61-65 |
| rs3807306 | 7 | 1.29E+08 | G | T | 0.506844 | 0.188383 | 0.15647 | 0.23 | Age 61-65 |
| rs3918226 | 7 | 1.51E+08 | C | T | 0.919429 | -0.66984 | 0.291622 | 0.022 | Age 61-65 |
| rs1991651 | 8 | 10706411 | C | G | 0.377755 | 0.291468 | 0.161212 | 0.071 | Age 61-65 |
| rs1352026 | 8 | 25895899 | C | T | 0.746306 | 0.356754 | 0.179989 | 0.047 | Age 61-65 |
| rs830444 | 8 | 76954113 | G | T | 0.396584 | -0.11483 | 0.159764 | 0.47 | Age 61-65 |
| rs35783704 | 8 | 1.06E+08 | G | A | 0.899221 | 1.09538 | 0.26245 | 3.00E-05 | Age 61-65 |
| rs62521878 | 8 | 1.42E+08 | C | T | 0.706412 | -0.21704 | 0.171673 | 0.21 | Age 61-65 |
| rs72762681 | 9 | 1.13E+08 | A | T | 0.85091 | -0.28056 | 0.220616 | 0.2 | Age 61-65 |
| rs1623474 | 10 | 18471794 | C | T | 0.670268 | -0.47892 | 0.225091 | 0.033 | Age >65 |
| rs12258967 | 10 | 18727959 | C | G | 0.700126 | 0.235218 | 0.230228 | 0.31 | Age >65 |
| rs57946343 | 10 | 63499951 | T | C | 0.848432 | 1.28257 | 0.293178 | 1.20E-05 | Age >65 |
| rs10128354 | 10 | 75470045 | T | C | 0.412385 | 0.438373 | 0.214915 | 0.041 | Age >65 |
| rs10882398 | 10 | 95892788 | T | A | 0.564355 | -0.4109 | 0.214024 | 0.055 | Age >65 |
| rs1006545 | 10 | 1.03E+08 | G | T | 0.111559 | 0.041389 | 0.337001 | 0.9 | Age >65 |
| rs11191551 | 10 | 1.05E+08 | A | G | 0.923453 | 1.67674 | 0.396302 | 2.30E-05 | Age >65 |
| rs1801253 | 10 | 1.16E+08 | G | C | 0.258676 | -0.21365 | 0.242996 | 0.38 | Age >65 |
| rs11592107 | 10 | 1.23E+08 | G | A | 0.685209 | -0.38402 | 0.226588 | 0.09 | Age >65 |
| rs592373 | 11 | 1890990 | G | A | 0.368887 | -0.9214 | 0.219166 | 2.60E-05 | Age >65 |
| rs2218305 | 11 | 10247896 | G | C | 0.506601 | -0.30858 | 0.212048 | 0.15 | Age >65 |
| rs1966698 | 11 | 16306267 | C | T | 0.207566 | 0.715609 | 0.259612 | 0.0058 | Age >65 |
| rs416258 | 11 | 16911636 | G | C | 0.698188 | -0.46085 | 0.22799 | 0.043 | Age >65 |
| rs7107356 | 11 | 47676170 | A | G | 0.501856 | -0.98546 | 0.211554 | 3.20E-06 | Age >65 |
| rs10750766 | 11 | 65473798 | C | A | 0.292234 | 0.107846 | 0.232241 | 0.64 | Age >65 |
| rs139437879 | 11 | 67904333 | G | A | 0.984055 | 1.32099 | 0.857959 | 0.12 | Age >65 |
| rs58941166 | 11 | 72083441 | G | A | 0.93359 | -0.5626 | 0.428927 | 0.19 | Age >65 |
| rs604723 | 11 | 1.01E+08 | T | C | 0.279517 | -0.76248 | 0.237908 | 0.0014 | Age >65 |
| rs2072927 | 1 | 1684800 | G | A | 0.503724 | 0.170255 | 0.211482 | 0.42 | Age >65 |
| rs488834 | 1 | 10767902 | C | T | 0.233611 | 0.34178 | 0.250265 | 0.17 | Age >65 |
| rs6669371 | 1 | 11882142 | T | G | 0.838288 | 1.29106 | 0.285824 | 6.30E-06 | Age >65 |
| rs75461554 | 1 | 15810172 | C | T | 0.801639 | 0.223785 | 0.265469 | 0.4 | Age >65 |
| rs4908348 | 1 | 28706949 | T | G | 0.697543 | 0.099364 | 0.229825 | 0.67 | Age >65 |
| rs1408945 | 1 | 42364877 | G | T | 0.569302 | 0.224946 | 0.212927 | 0.29 | Age >65 |
| rs12132894 | 1 | 89138474 | A | T | 0.57677 | 0.277059 | 0.215813 | 0.2 | Age >65 |
| rs10776752 | 1 | 1.13E+08 | G | T | 0.924431 | -1.38419 | 0.399412 | 0.00053 | Age >65 |
| rs10914117 | 1 | 1.81E+08 | T | C | 0.595513 | -0.02509 | 0.214774 | 0.91 | Age >65 |
| rs68085857 | 1 | 2.18E+08 | C | T | 0.768894 | -0.36084 | 0.251307 | 0.15 | Age >65 |
| rs1745417 | 1 | 2.28E+08 | C | T | 0.487412 | -0.39161 | 0.211574 | 0.064 | Age >65 |
| rs699 | 1 | 2.31E+08 | A | G | 0.601111 | -0.64295 | 0.216612 | 0.003 | Age >65 |
| rs3213751 | 12 | 432666 | G | C | 0.739665 | 0.046905 | 0.239749 | 0.84 | Age >65 |
| rs11055034 | 12 | 12890626 | C | A | 0.719917 | 0.133711 | 0.237113 | 0.57 | Age >65 |
| rs1010064 | 12 | 20000315 | A | C | 0.81476 | 0.646644 | 0.269835 | 0.017 | Age >65 |
| rs73075659 | 12 | 20373541 | A | G | 0.654925 | 0.241755 | 0.222404 | 0.28 | Age >65 |
| rs150857355 | 12 | 49209340 | G | C | 0.97729 | -1.43004 | 0.7204 | 0.047 | Age >65 |
| rs7967954 | 12 | 50673484 | G | A | 0.421356 | 0.316373 | 0.213063 | 0.14 | Age >65 |
| rs736825 | 12 | 54417576 | C | G | 0.640825 | 0.284226 | 0.222921 | 0.2 | Age >65 |
| rs4143175 | 12 | 67782397 | T | C | 0.239201 | 0.278798 | 0.249238 | 0.26 | Age >65 |
| rs17249754 | 12 | 90060586 | G | A | 0.828801 | 0.827688 | 0.280763 | 0.0032 | Age >65 |
| rs7310615 | 12 | 1.12E+08 | C | G | 0.479545 | 0.53756 | 0.211835 | 0.011 | Age >65 |
| rs35443 | 12 | 1.16E+08 | G | C | 0.618018 | 0.765636 | 0.217092 | 0.00042 | Age >65 |
| rs6490021 | 12 | 1.16E+08 | A | C | 0.385312 | -0.19399 | 0.217512 | 0.37 | Age >65 |
| rs9508495 | 13 | 30146201 | C | T | 0.238713 | 0.480416 | 0.247197 | 0.052 | Age >65 |
| rs4415898 | 13 | 41895177 | T | A | 0.97808 | -0.01738 | 0.816447 | 0.98 | Age >65 |
| rs4415898 | 13 | 41895177 | T | G | 0.172875 | -0.46934 | 0.27997 | 0.094 | Age >65 |
| rs8904 | 14 | 35871217 | G | A | 0.636954 | -0.04588 | 0.22001 | 0.83 | Age >65 |
| rs28365937 | 14 | 53418233 | C | A | 0.700266 | -0.08024 | 0.231449 | 0.73 | Age >65 |
| rs12591513 | 15 | 75102714 | G | A | 0.332509 | 0.044265 | 0.225008 | 0.84 | Age >65 |
| rs2627316 | 15 | 81042816 | A | G | 0.531891 | -0.7378 | 0.212607 | 0.00052 | Age >65 |
| rs4932373 | 15 | 91429287 | A | C | 0.682128 | -0.51215 | 0.227949 | 0.025 | Age >65 |
| rs10500326 | 16 | 4918326 | G | T | 0.759623 | 0.259797 | 0.249078 | 0.3 | Age >65 |
| rs4293393 | 16 | 20364588 | A | G | 0.816486 | 0.625379 | 0.274749 | 0.023 | Age >65 |
| rs4888408 | 16 | 75432824 | G | A | 0.407409 | -0.25471 | 0.214864 | 0.24 | Age >65 |
| rs9806885 | 16 | 81556909 | A | G | 0.888147 | 0.163261 | 0.334607 | 0.63 | Age >65 |
| rs908951 | 16 | 89697625 | C | T | 0.572603 | 0.07078 | 0.215534 | 0.74 | Age >65 |
| rs222857 | 17 | 7164563 | C | T | 0.429361 | -0.43447 | 0.213802 | 0.042 | Age >65 |
| rs7213273 | 17 | 43155914 | G | A | 0.342963 | 0.553656 | 0.22219 | 0.013 | Age >65 |
| rs17608766 | 17 | 45013271 | T | C | 0.854478 | -0.86624 | 0.299174 | 0.0038 | Age >65 |
| rs7406910 | 17 | 46688256 | T | C | 0.088561 | -0.70633 | 0.371487 | 0.057 | Age >65 |
| rs2671661 | 17 | 47528562 | T | C | 0.270868 | -0.28753 | 0.239942 | 0.23 | Age >65 |
| rs1000423 | 17 | 59475642 | C | T | 0.267996 | -0.85118 | 0.238811 | 0.00036 | Age >65 |
| rs77946446 | 17 | 75317932 | C | T | 0.733126 | 0.235755 | 0.240312 | 0.33 | Age >65 |
| rs12955644 | 18 | 771047 | C | G | 0.813461 | 0.636557 | 0.274022 | 0.02 | Age >65 |
| rs56407827 | 18 | 42179819 | C | T | 0.732837 | -0.09875 | 0.24192 | 0.68 | Age >65 |
| rs76978865 | 19 | 2175799 | G | A | 0.959727 | -0.26585 | 0.53526 | 0.62 | Age >65 |
| rs10427021 | 19 | 7259346 | T | G | 0.88749 | 1.44147 | 0.366619 | 8.40E-05 | Age >65 |
| rs79709447 | 19 | 12187621 | T | C | 0.988197 | -0.74478 | 0.993907 | 0.45 | Age >65 |
| rs1327235 | 20 | 10969030 | A | G | 0.520935 | -0.38946 | 0.212563 | 0.067 | Age >65 |
| rs17812022 | 20 | 19007099 | C | T | 0.904259 | 0.636741 | 0.359556 | 0.077 | Age >65 |
| rs6102506 | 20 | 40254735 | C | T | 0.653028 | 0.822138 | 0.223277 | 0.00023 | Age >65 |
| rs6026744 | 20 | 57742388 | A | T | 0.882268 | -0.74721 | 0.329436 | 0.023 | Age >65 |
| rs1275988 | 2 | 26914364 | C | T | 0.384326 | 0.579072 | 0.218528 | 0.0081 | Age >65 |
| rs35441226 | 2 | 43191137 | C | T | 0.817646 | 0.300023 | 0.275719 | 0.28 | Age >65 |
| rs56308618 | 21 | 44757792 | G | A | 0.710984 | -0.12355 | 0.233733 | 0.6 | Age >65 |
| rs58117425 | 2 | 1.46E+08 | G | A | 0.764401 | -0.29547 | 0.250361 | 0.24 | Age >65 |
| rs73029563 | 2 | 1.65E+08 | C | G | 0.458215 | -0.41376 | 0.213395 | 0.053 | Age >65 |
| rs10205122 | 2 | 1.81E+08 | A | G | 0.715566 | 0.502647 | 0.235349 | 0.033 | Age >65 |
| rs7592578 | 2 | 1.91E+08 | T | G | 0.187298 | -0.34123 | 0.273418 | 0.21 | Age >65 |
| rs3845811 | 2 | 2.09E+08 | C | G | 0.561857 | -0.02111 | 0.212642 | 0.92 | Age >65 |
| rs2161967 | 2 | 2.19E+08 | T | G | 0.423263 | 0.130108 | 0.214721 | 0.54 | Age >65 |
| rs2594992 | 3 | 11360997 | A | C | 0.600875 | -0.07853 | 0.215194 | 0.72 | Age >65 |
| rs34991912 | 3 | 14926351 | T | C | 0.414709 | 0.359376 | 0.213307 | 0.092 | Age >65 |
| rs2643826 | 3 | 27562988 | C | T | 0.546892 | -0.67171 | 0.212991 | 0.0016 | Age >65 |
| rs6771917 | 3 | 48108442 | T | C | 0.241534 | -0.48341 | 0.248547 | 0.052 | Age >65 |
| rs3772219 | 3 | 56771251 | A | C | 0.677409 | 0.225823 | 0.225217 | 0.32 | Age >65 |
| rs73872710 | 3 | 1.41E+08 | A | T | 0.925727 | -0.31096 | 0.402371 | 0.44 | Age >65 |
| rs1597467 | 3 | 1.5E+08 | C | A | 0.906595 | 0.803472 | 0.364404 | 0.027 | Age >65 |
| rs11715377 | 3 | 1.69E+08 | A | T | 0.906154 | 0.536965 | 0.36219 | 0.14 | Age >65 |
| rs1528293 | 3 | 1.69E+08 | A | T | 0.487678 | 0.057773 | 0.210903 | 0.78 | Age >65 |
| rs16852391 | 4 | 40604309 | C | G | 0.895197 | 0.058111 | 0.344431 | 0.87 | Age >65 |
| rs12509595 | 4 | 81182554 | T | C | 0.710031 | -0.85475 | 0.232052 | 0.00023 | Age >65 |
| rs17010957 | 4 | 86719165 | T | C | 0.850965 | -0.30758 | 0.300716 | 0.31 | Age >65 |
| rs13135092 | 4 | 1.03E+08 | A | G | 0.917786 | 0.491914 | 0.387325 | 0.2 | Age >65 |
| rs6820343 | 4 | 1.56E+08 | C | T | 0.465224 | 0.312598 | 0.211466 | 0.14 | Age >65 |
| rs72689147 | 4 | 1.57E+08 | G | T | 0.80966 | 0.350643 | 0.269639 | 0.19 | Age >65 |
| rs10069690 | 5 | 1279790 | C | T | 0.740738 | -0.09144 | 0.239426 | 0.7 | Age >65 |
| rs7733331 | 5 | 32828846 | T | C | 0.402057 | -0.67708 | 0.216272 | 0.0017 | Age >65 |
| rs2112453 | 5 | 1.12E+08 | G | A | 0.264645 | 0.111045 | 0.240686 | 0.64 | Age >65 |
| rs4487481 | 5 | 1.14E+08 | T | C | 0.478844 | 0.588958 | 0.21124 | 0.0053 | Age >65 |
| rs6890251 | 5 | 1.22E+08 | C | T | 0.851228 | -0.43756 | 0.296474 | 0.14 | Age >65 |
| rs7703751 | 5 | 1.23E+08 | A | T | 0.74255 | 0.051138 | 0.242239 | 0.83 | Age >65 |
| rs6892983 | 5 | 1.28E+08 | C | A | 0.58922 | -0.58619 | 0.214605 | 0.0063 | Age >65 |
| rs7737361 | 5 | 1.48E+08 | G | A | 0.803884 | 0.011481 | 0.271034 | 0.97 | Age >65 |
| rs1650581 | 5 | 1.58E+08 | C | G | 0.741699 | -0.46853 | 0.243003 | 0.054 | Age >65 |
| rs12187017 | 5 | 1.58E+08 | G | A | 0.636354 | 0.719629 | 0.220248 | 0.0011 | Age >65 |
| rs198851 | 6 | 26104632 | T | G | 0.150225 | 0.394279 | 0.296848 | 0.18 | Age >65 |
| rs1265157 | 6 | 31142265 | C | G | 0.650614 | -0.47026 | 0.221683 | 0.034 | Age >65 |
| rs2856448 | 6 | 32014575 | A | G | 0.534882 | -0.19677 | 0.211898 | 0.35 | Age >65 |
| rs1214761 | 6 | 43354431 | A | G | 0.3195 | 0.536442 | 0.226691 | 0.018 | Age >65 |
| rs211159 | 6 | 97023026 | G | A | 0.656462 | -0.2389 | 0.223601 | 0.29 | Age >65 |
| rs4897160 | 6 | 1.26E+08 | G | A | 0.52738 | -0.2561 | 0.211131 | 0.23 | Age >65 |
| rs9375459 | 6 | 1.27E+08 | C | T | 0.567495 | -0.68449 | 0.212803 | 0.0013 | Age >65 |
| rs62434129 | 6 | 1.51E+08 | A | T | 0.929936 | 1.17495 | 0.413729 | 0.0045 | Age >65 |
| rs2969036 | 7 | 2534901 | T | G | 0.3128 | 0.338615 | 0.22773 | 0.14 | Age >65 |
| rs3735533 | 7 | 27245893 | T | C | 0.07394 | -1.55623 | 0.402957 | 0.00011 | Age >65 |
| rs6961048 | 7 | 27328187 | C | G | 0.899227 | -0.45503 | 0.350105 | 0.19 | Age >65 |
| rs2392929 | 7 | 1.06E+08 | T | G | 0.803595 | -1.0901 | 0.26749 | 4.60E-05 | Age >65 |
| rs3807306 | 7 | 1.29E+08 | G | T | 0.5049 | 0.168368 | 0.212525 | 0.43 | Age >65 |
| rs3918226 | 7 | 1.51E+08 | C | T | 0.92006 | -1.22822 | 0.393828 | 0.0018 | Age >65 |
| rs1991651 | 8 | 10706411 | C | G | 0.381678 | 0.074096 | 0.217734 | 0.73 | Age >65 |
| rs1352026 | 8 | 25895899 | C | T | 0.747155 | 0.143724 | 0.242977 | 0.55 | Age >65 |
| rs830444 | 8 | 76954113 | G | T | 0.395612 | -0.53982 | 0.2164 | 0.013 | Age >65 |
| rs35783704 | 8 | 1.06E+08 | G | A | 0.8999 | 0.912598 | 0.356658 | 0.011 | Age >65 |
| rs62521878 | 8 | 1.42E+08 | C | T | 0.703848 | 0.380115 | 0.232205 | 0.1 | Age >65 |
| rs72762681 | 9 | 1.13E+08 | A | T | 0.849037 | -0.67885 | 0.295429 | 0.022 | Age >65 |

**Supplemental Table 2**. Genetic instruments used for LDL-C for analyses of total CHD

| SNP | CHR | BP | ALLELE1 | ALLELE0 | A1FREQ | BETA | SE | P | phenotype |
| --- | --- | --- | --- | --- | --- | --- | --- | --- | --- |
| rs1129555 | 10 | 1.14E+08 | A | G | 0.275965 | 0.024064 | 0.005212 | 3.90E-06 | Age ≤55 |
| rs10128711 | 11 | 18632984 | T | C | 0.260873 | -0.02324 | 0.005292 | 1.10E-05 | Age ≤55 |
| rs149803 | 11 | 61539020 | C | G | 0.74408 | -0.0168 | 0.00542 | 0.0019 | Age ≤55 |
| rs499790 | 11 | 1.17E+08 | C | T | 0.897048 | -0.03587 | 0.00778 | 4.00E-06 | Age ≤55 |
| rs61905078 | 11 | 1.17E+08 | A | C | 0.926432 | -0.07181 | 0.008901 | 7.20E-16 | Age ≤55 |
| rs11220437 | 11 | 1.26E+08 | T | C | 0.885994 | -0.0285 | 0.007326 | 1.00E-04 | Age ≤55 |
| rs3093584 | 1 | 25668714 | T | C | 0.557348 | 0.021704 | 0.004633 | 2.80E-06 | Age ≤55 |
| rs12753981 | 1 | 27001893 | G | A | 0.915917 | -0.02442 | 0.008314 | 0.0033 | Age ≤55 |
| rs2495504 | 1 | 55485796 | C | T | 0.279206 | 0.027868 | 0.005148 | 6.20E-08 | Age ≤55 |
| rs2479408 | 1 | 55504188 | C | G | 0.788328 | 0.028854 | 0.005751 | 5.20E-07 | Age ≤55 |
| rs2495477 | 1 | 55518467 | A | G | 0.607571 | 0.041614 | 0.004822 | 6.20E-18 | Age ≤55 |
| rs626787 | 1 | 62901243 | C | G | 0.647505 | 0.050135 | 0.004849 | 4.60E-25 | Age ≤55 |
| rs1386585 | 1 | 92715207 | C | T | 0.193212 | -0.00969 | 0.005843 | 0.097 | Age ≤55 |
| rs1337247 | 1 | 1.1E+08 | C | A | 0.854437 | 0.050552 | 0.006532 | 1.00E-14 | Age ≤55 |
| rs4268379 | 1 | 1.1E+08 | C | T | 0.49658 | -0.03981 | 0.00461 | 5.80E-18 | Age ≤55 |
| rs267733 | 1 | 1.51E+08 | A | G | 0.839415 | 0.024299 | 0.00629 | 0.00011 | Age ≤55 |
| rs2642438 | 1 | 2.21E+08 | A | G | 0.296443 | -0.02962 | 0.005041 | 4.20E-09 | Age ≤55 |
| rs487738 | 1 | 2.35E+08 | A | G | 0.329031 | -0.03826 | 0.005182 | 1.50E-13 | Age ≤55 |
| rs3184504 | 12 | 1.12E+08 | T | C | 0.480964 | -0.01647 | 0.004653 | 4.00E-04 | Age ≤55 |
| rs2649999 | 12 | 1.21E+08 | T | C | 0.332107 | 0.027985 | 0.005016 | 2.40E-08 | Age ≤55 |
| rs206070 | 13 | 32896846 | G | A | 0.82013 | 0.002917 | 0.006076 | 0.63 | Age ≤55 |
| rs6573778 | 14 | 24872209 | T | C | 0.481042 | 0.018698 | 0.004672 | 6.30E-05 | Age ≤55 |
| rs9989419 | 16 | 56985139 | A | G | 0.394698 | 0.021309 | 0.004742 | 7.00E-06 | Age ≤55 |
| rs9932707 | 16 | 71862133 | A | C | 0.541564 | -0.02812 | 0.004685 | 1.90E-09 | Age ≤55 |
| rs4788597 | 16 | 72043039 | C | T | 0.638186 | 0.03114 | 0.004859 | 1.50E-10 | Age ≤55 |
| rs11650232 | 17 | 7088923 | A | G | 0.556048 | -0.00824 | 0.004659 | 0.077 | Age ≤55 |
| rs7225700 | 17 | 45391804 | T | C | 0.352439 | -0.02181 | 0.00485 | 6.90E-06 | Age ≤55 |
| rs1801689 | 17 | 64210580 | A | C | 0.969808 | -0.09226 | 0.013517 | 8.80E-12 | Age ≤55 |
| rs2886232 | 17 | 67150176 | T | C | 0.12207 | 0.037454 | 0.007109 | 1.40E-07 | Age ≤55 |
| rs17677316 | 19 | 10680241 | G | A | 0.752296 | -0.01236 | 0.005332 | 0.02 | Age ≤55 |
| rs1529711 | 19 | 11023434 | C | T | 0.838572 | -0.02715 | 0.006244 | 1.40E-05 | Age ≤55 |
| rs17657025 | 19 | 44675801 | T | C | 0.92085 | 0.044481 | 0.008523 | 1.80E-07 | Age ≤55 |
| rs1004165 | 19 | 45232205 | G | A | 0.561739 | -0.03392 | 0.004663 | 3.50E-13 | Age ≤55 |
| rs11668536 | 19 | 45328476 | C | T | 0.752992 | 0.020175 | 0.005351 | 0.00016 | Age ≤55 |
| rs6857 | 19 | 45392254 | C | T | 0.829655 | -0.18785 | 0.006148 | 4.90E-205 | Age ≤55 |
| rs364585 | 20 | 12962718 | A | G | 0.392457 | -0.00871 | 0.004768 | 0.068 | Age ≤55 |
| rs2328223 | 20 | 17845921 | A | C | 0.813993 | -0.01812 | 0.005973 | 0.0024 | Age ≤55 |
| rs7264396 | 20 | 34154741 | C | T | 0.790192 | 0.021747 | 0.005722 | 0.00014 | Age ≤55 |
| rs6016373 | 20 | 39154095 | A | G | 0.592107 | 0.032986 | 0.004816 | 7.40E-12 | Age ≤55 |
| rs12624863 | 20 | 39782899 | A | G | 0.792027 | -0.0295 | 0.00573 | 2.60E-07 | Age ≤55 |
| rs1800961 | 20 | 43042364 | C | T | 0.969443 | 0.071757 | 0.013499 | 1.10E-07 | Age ≤55 |
| rs13027716 | 2 | 20887226 | T | G | 0.969769 | 0.073542 | 0.013786 | 9.60E-08 | Age ≤55 |
| rs11124924 | 2 | 21206275 | G | C | 0.94 | 0.072196 | 0.009715 | 1.10E-13 | Age ≤55 |
| rs12720842 | 2 | 21257927 | T | C | 0.962884 | -0.07631 | 0.01221 | 4.10E-10 | Age ≤55 |
| rs6756743 | 2 | 21301892 | C | T | 0.952928 | -0.03201 | 0.010951 | 0.0035 | Age ≤55 |
| rs10495907 | 2 | 43998726 | G | A | 0.868103 | -0.01568 | 0.006832 | 0.022 | Age ≤55 |
| rs3923912 | 2 | 44057570 | A | C | 0.937914 | 0.119502 | 0.00962 | 2.00E-35 | Age ≤55 |
| rs4148217 | 2 | 44099433 | C | A | 0.816565 | 0.035269 | 0.005971 | 3.50E-09 | Age ≤55 |
| rs11125936 | 2 | 62871225 | T | C | 0.907479 | 0.036535 | 0.007939 | 4.20E-06 | Age ≤55 |
| rs17508045 | 2 | 1.19E+08 | T | C | 0.91238 | 0.047571 | 0.008186 | 6.20E-09 | Age ≤55 |
| rs16831243 | 2 | 1.36E+08 | C | T | 0.898 | -0.0107 | 0.007699 | 0.16 | Age ≤55 |
| rs10195252 | 2 | 1.66E+08 | T | C | 0.59432 | 0.020947 | 0.004698 | 8.30E-06 | Age ≤55 |
| rs1250229 | 2 | 2.16E+08 | T | C | 0.260144 | -0.01596 | 0.00529 | 0.0025 | Age ≤55 |
| rs11563251 | 2 | 2.35E+08 | C | T | 0.889225 | -0.0252 | 0.007335 | 0.00059 | Age ≤55 |
| rs4823057 | 22 | 30241061 | A | G | 0.976935 | -0.05623 | 0.015621 | 0.00032 | Age ≤55 |
| rs4253772 | 22 | 46627603 | C | T | 0.88992 | -0.0177 | 0.007437 | 0.017 | Age ≤55 |
| rs7616006 | 3 | 12267648 | A | G | 0.578592 | 0.026956 | 0.004701 | 9.80E-09 | Age ≤55 |
| rs7640978 | 3 | 32533010 | C | T | 0.90992 | 0.032805 | 0.008098 | 5.10E-05 | Age ≤55 |
| rs17404153 | 3 | 1.32E+08 | G | T | 0.875465 | 0.025585 | 0.007029 | 0.00027 | Age ≤55 |
| rs6818397 | 4 | 3434885 | T | G | 0.385261 | 0.021309 | 0.004821 | 9.90E-06 | Age ≤55 |
| rs4072296 | 5 | 74242002 | T | C | 0.457586 | -0.02505 | 0.004806 | 1.90E-07 | Age ≤55 |
| rs16872670 | 5 | 74929312 | G | A | 0.952806 | -0.02285 | 0.01099 | 0.038 | Age ≤55 |
| rs4530754 | 5 | 1.23E+08 | G | A | 0.455421 | -0.01572 | 0.004651 | 0.00072 | Age ≤55 |
| rs4704810 | 5 | 1.56E+08 | G | A | 0.627687 | -0.01818 | 0.004864 | 0.00019 | Age ≤55 |
| rs2294266 | 6 | 16107867 | A | C | 0.51108 | 0.032636 | 0.004669 | 2.70E-12 | Age ≤55 |
| rs1408272 | 6 | 25842951 | T | G | 0.922153 | 0.055923 | 0.008648 | 1.00E-10 | Age ≤55 |
| rs1049281 | 6 | 31236567 | T | C | 0.360189 | -0.01685 | 0.004838 | 5.00E-04 | Age ≤55 |
| rs3798236 | 6 | 1.16E+08 | T | C | 0.630485 | 0.015946 | 0.004842 | 0.00099 | Age ≤55 |
| rs6917747 | 6 | 1.6E+08 | G | A | 0.863325 | -0.03779 | 0.006786 | 2.60E-08 | Age ≤55 |
| rs9347386 | 6 | 1.61E+08 | A | G | 0.62334 | 0.028288 | 0.004815 | 4.20E-09 | Age ≤55 |
| rs6415084 | 6 | 1.61E+08 | T | C | 0.479357 | 0.025637 | 0.004662 | 3.80E-08 | Age ≤55 |
| rs2390536 | 7 | 21485397 | G | A | 0.63617 | -0.02342 | 0.004835 | 1.30E-06 | Age ≤55 |
| rs4722551 | 7 | 25991826 | T | C | 0.841691 | -0.03745 | 0.006355 | 3.80E-09 | Age ≤55 |
| rs7798185 | 7 | 44570717 | C | A | 0.829484 | -0.04235 | 0.006178 | 7.10E-12 | Age ≤55 |
| rs713286 | 8 | 9171735 | T | C | 0.920193 | 0.015817 | 0.00868 | 0.068 | Age ≤55 |
| rs10102164 | 8 | 55421614 | G | A | 0.788015 | -0.03151 | 0.005673 | 2.80E-08 | Age ≤55 |
| rs2737229 | 8 | 1.17E+08 | A | C | 0.701915 | 0.029474 | 0.005069 | 6.10E-09 | Age ≤55 |
| rs17320616 | 8 | 1.26E+08 | A | G | 0.796535 | -0.03656 | 0.005787 | 2.60E-10 | Age ≤55 |
| rs7014582 | 8 | 1.45E+08 | A | G | 0.567125 | -0.01908 | 0.004696 | 4.80E-05 | Age ≤55 |
| rs3780181 | 9 | 2640759 | A | G | 0.930901 | 0.041956 | 0.009213 | 5.30E-06 | Age ≤55 |
| rs12686004 | 9 | 1.08E+08 | G | A | 0.884733 | 0.034931 | 0.007297 | 1.70E-06 | Age ≤55 |
| rs7849542 | 9 | 1.36E+08 | G | A | 0.700344 | -0.01802 | 0.005079 | 0.00039 | Age ≤55 |
| rs7030248 | 9 | 1.36E+08 | G | A | 0.656126 | 0.016603 | 0.004919 | 0.00074 | Age ≤55 |
| rs1129555 | 10 | 1.14E+08 | A | G | 0.27562 | -0.0035 | 0.007965 | 0.66 | Age 56-60 |
| rs10128711 | 11 | 18632984 | T | C | 0.259375 | -0.02481 | 0.008116 | 0.0022 | Age 56-60 |
| rs149803 | 11 | 61539020 | C | G | 0.743375 | -0.03239 | 0.008339 | 1.00E-04 | Age 56-60 |
| rs499790 | 11 | 1.17E+08 | C | T | 0.894883 | -0.04026 | 0.011751 | 0.00061 | Age 56-60 |
| rs61905078 | 11 | 1.17E+08 | A | C | 0.927387 | -0.06824 | 0.013694 | 6.20E-07 | Age 56-60 |
| rs11220437 | 11 | 1.26E+08 | T | C | 0.882322 | -0.03606 | 0.010991 | 0.001 | Age 56-60 |
| rs3093584 | 1 | 25668714 | T | C | 0.559415 | 0.026753 | 0.007147 | 0.00018 | Age 56-60 |
| rs12753981 | 1 | 27001893 | G | A | 0.915958 | -0.00421 | 0.012787 | 0.74 | Age 56-60 |
| rs2495504 | 1 | 55485796 | C | T | 0.280429 | 0.044087 | 0.007875 | 2.20E-08 | Age 56-60 |
| rs2479408 | 1 | 55504188 | C | G | 0.789527 | 0.033921 | 0.008871 | 0.00013 | Age 56-60 |
| rs2495477 | 1 | 55518467 | A | G | 0.608763 | 0.033455 | 0.007375 | 5.70E-06 | Age 56-60 |
| rs626787 | 1 | 62901243 | C | G | 0.649038 | 0.058237 | 0.007415 | 4.00E-15 | Age 56-60 |
| rs1386585 | 1 | 92715207 | C | T | 0.195635 | -0.02282 | 0.008931 | 0.011 | Age 56-60 |
| rs1337247 | 1 | 1.1E+08 | C | A | 0.85498 | 0.056199 | 0.010071 | 2.40E-08 | Age 56-60 |
| rs4268379 | 1 | 1.1E+08 | C | T | 0.497852 | -0.04666 | 0.007123 | 5.70E-11 | Age 56-60 |
| rs267733 | 1 | 1.51E+08 | A | G | 0.838062 | 0.0098 | 0.009606 | 0.31 | Age 56-60 |
| rs2642438 | 1 | 2.21E+08 | A | G | 0.296932 | -0.04155 | 0.00774 | 8.00E-08 | Age 56-60 |
| rs487738 | 1 | 2.35E+08 | A | G | 0.327503 | -0.0254 | 0.007947 | 0.0014 | Age 56-60 |
| rs3184504 | 12 | 1.12E+08 | T | C | 0.484365 | -0.0198 | 0.007084 | 0.0052 | Age 56-60 |
| rs2649999 | 12 | 1.21E+08 | T | C | 0.328788 | 0.034509 | 0.007701 | 7.40E-06 | Age 56-60 |
| rs206070 | 13 | 32896846 | G | A | 0.821675 | -0.01456 | 0.009298 | 0.12 | Age 56-60 |
| rs6573778 | 14 | 24872209 | T | C | 0.483162 | 0.023242 | 0.00717 | 0.0012 | Age 56-60 |
| rs9989419 | 16 | 56985139 | A | G | 0.395817 | 0.023275 | 0.007223 | 0.0013 | Age 56-60 |
| rs9932707 | 16 | 71862133 | A | C | 0.543565 | -0.04146 | 0.00712 | 5.80E-09 | Age 56-60 |
| rs4788597 | 16 | 72043039 | C | T | 0.634787 | 0.030287 | 0.007417 | 4.40E-05 | Age 56-60 |
| rs11650232 | 17 | 7088923 | A | G | 0.553044 | -0.01085 | 0.007172 | 0.13 | Age 56-60 |
| rs7225700 | 17 | 45391804 | T | C | 0.355028 | -0.03063 | 0.007401 | 3.50E-05 | Age 56-60 |
| rs1801689 | 17 | 64210580 | A | C | 0.969665 | -0.06759 | 0.02076 | 0.0011 | Age 56-60 |
| rs2886232 | 17 | 67150176 | T | C | 0.124656 | 0.043224 | 0.010737 | 5.70E-05 | Age 56-60 |
| rs17677316 | 19 | 10680241 | G | A | 0.75396 | -0.03133 | 0.008163 | 0.00012 | Age 56-60 |
| rs1529711 | 19 | 11023434 | C | T | 0.84127 | -0.0147 | 0.009683 | 0.13 | Age 56-60 |
| rs17657025 | 19 | 44675801 | T | C | 0.920195 | 0.048201 | 0.013078 | 0.00023 | Age 56-60 |
| rs1004165 | 19 | 45232205 | G | A | 0.561897 | -0.04121 | 0.007128 | 7.40E-09 | Age 56-60 |
| rs11668536 | 19 | 45328476 | C | T | 0.752069 | 0.016945 | 0.008229 | 0.039 | Age 56-60 |
| rs6857 | 19 | 45392254 | C | T | 0.829355 | -0.2104 | 0.009434 | 3.50E-110 | Age 56-60 |
| rs364585 | 20 | 12962718 | A | G | 0.393233 | -0.01835 | 0.007317 | 0.012 | Age 56-60 |
| rs2328223 | 20 | 17845921 | A | C | 0.812713 | -0.01349 | 0.009097 | 0.14 | Age 56-60 |
| rs7264396 | 20 | 34154741 | C | T | 0.789996 | 0.024115 | 0.008744 | 0.0058 | Age 56-60 |
| rs6016373 | 20 | 39154095 | A | G | 0.597261 | 0.024469 | 0.007436 | 0.001 | Age 56-60 |
| rs12624863 | 20 | 39782899 | A | G | 0.789542 | -0.02481 | 0.008701 | 0.0044 | Age 56-60 |
| rs1800961 | 20 | 43042364 | C | T | 0.968364 | 0.059171 | 0.020228 | 0.0034 | Age 56-60 |
| rs13027716 | 2 | 20887226 | T | G | 0.970266 | 0.042052 | 0.021335 | 0.049 | Age 56-60 |
| rs11124924 | 2 | 21206275 | G | C | 0.938029 | 0.078322 | 0.014748 | 1.10E-07 | Age 56-60 |
| rs12720842 | 2 | 21257927 | T | C | 0.964716 | -0.06295 | 0.019243 | 0.0011 | Age 56-60 |
| rs6756743 | 2 | 21301892 | C | T | 0.952916 | -0.05748 | 0.016836 | 0.00064 | Age 56-60 |
| rs10495907 | 2 | 43998726 | G | A | 0.869004 | -0.03344 | 0.010516 | 0.0015 | Age 56-60 |
| rs3923912 | 2 | 44057570 | A | C | 0.938554 | 0.106682 | 0.014877 | 7.50E-13 | Age 56-60 |
| rs4148217 | 2 | 44099433 | C | A | 0.817094 | 0.035013 | 0.009141 | 0.00013 | Age 56-60 |
| rs11125936 | 2 | 62871225 | T | C | 0.90401 | 0.026763 | 0.011975 | 0.025 | Age 56-60 |
| rs17508045 | 2 | 1.19E+08 | T | C | 0.91285 | 0.046884 | 0.012527 | 0.00018 | Age 56-60 |
| rs16831243 | 2 | 1.36E+08 | C | T | 0.900158 | -0.02085 | 0.011886 | 0.079 | Age 56-60 |
| rs10195252 | 2 | 1.66E+08 | T | C | 0.593882 | 0.003269 | 0.007224 | 0.65 | Age 56-60 |
| rs1250229 | 2 | 2.16E+08 | T | C | 0.260933 | -0.01296 | 0.008071 | 0.11 | Age 56-60 |
| rs11563251 | 2 | 2.35E+08 | C | T | 0.888907 | -0.02058 | 0.011248 | 0.067 | Age 56-60 |
| rs4823057 | 22 | 30241061 | A | G | 0.976879 | 0.02294 | 0.023814 | 0.34 | Age 56-60 |
| rs4253772 | 22 | 46627603 | C | T | 0.889457 | -0.00405 | 0.011257 | 0.72 | Age 56-60 |
| rs7616006 | 3 | 12267648 | A | G | 0.57829 | 0.015517 | 0.007188 | 0.031 | Age 56-60 |
| rs7640978 | 3 | 32533010 | C | T | 0.91092 | 0.03266 | 0.012461 | 0.0088 | Age 56-60 |
| rs17404153 | 3 | 1.32E+08 | G | T | 0.876045 | 0.005783 | 0.010811 | 0.59 | Age 56-60 |
| rs6818397 | 4 | 3434885 | T | G | 0.386914 | 0.015579 | 0.007345 | 0.034 | Age 56-60 |
| rs4072296 | 5 | 74242002 | T | C | 0.458183 | -0.02495 | 0.007358 | 7.00E-04 | Age 56-60 |
| rs16872670 | 5 | 74929312 | G | A | 0.952478 | -0.04963 | 0.016598 | 0.0028 | Age 56-60 |
| rs4530754 | 5 | 1.23E+08 | G | A | 0.454525 | -0.02845 | 0.007086 | 6.00E-05 | Age 56-60 |
| rs4704810 | 5 | 1.56E+08 | G | A | 0.627751 | -0.00786 | 0.007407 | 0.29 | Age 56-60 |
| rs2294266 | 6 | 16107867 | A | C | 0.508267 | 0.01294 | 0.00714 | 0.07 | Age 56-60 |
| rs1408272 | 6 | 25842951 | T | G | 0.923421 | 0.062857 | 0.013396 | 2.70E-06 | Age 56-60 |
| rs1049281 | 6 | 31236567 | T | C | 0.35575 | -0.01847 | 0.007437 | 0.013 | Age 56-60 |
| rs3798236 | 6 | 1.16E+08 | T | C | 0.636079 | 0.021559 | 0.007391 | 0.0035 | Age 56-60 |
| rs6917747 | 6 | 1.6E+08 | G | A | 0.864071 | -0.04598 | 0.010342 | 8.80E-06 | Age 56-60 |
| rs9347386 | 6 | 1.61E+08 | A | G | 0.620374 | 0.031886 | 0.007336 | 1.40E-05 | Age 56-60 |
| rs6415084 | 6 | 1.61E+08 | T | C | 0.482092 | 0.014179 | 0.00714 | 0.047 | Age 56-60 |
| rs2390536 | 7 | 21485397 | G | A | 0.636938 | -0.01849 | 0.007354 | 0.012 | Age 56-60 |
| rs4722551 | 7 | 25991826 | T | C | 0.843469 | -0.03093 | 0.009771 | 0.0015 | Age 56-60 |
| rs7798185 | 7 | 44570717 | C | A | 0.829099 | -0.03466 | 0.009438 | 0.00024 | Age 56-60 |
| rs713286 | 8 | 9171735 | T | C | 0.922466 | 0.043337 | 0.013515 | 0.0013 | Age 56-60 |
| rs10102164 | 8 | 55421614 | G | A | 0.789233 | -0.01774 | 0.008758 | 0.043 | Age 56-60 |
| rs2737229 | 8 | 1.17E+08 | A | C | 0.698504 | 0.014045 | 0.00774 | 0.07 | Age 56-60 |
| rs17320616 | 8 | 1.26E+08 | A | G | 0.797943 | -0.02927 | 0.008878 | 0.00098 | Age 56-60 |
| rs7014582 | 8 | 1.45E+08 | A | G | 0.564141 | -0.00596 | 0.007191 | 0.41 | Age 56-60 |
| rs3780181 | 9 | 2640759 | A | G | 0.931586 | 0.051692 | 0.014281 | 0.00029 | Age 56-60 |
| rs12686004 | 9 | 1.08E+08 | G | A | 0.886634 | 0.033769 | 0.011274 | 0.0027 | Age 56-60 |
| rs7849542 | 9 | 1.36E+08 | G | A | 0.700579 | -0.017 | 0.007801 | 0.029 | Age 56-60 |
| rs7030248 | 9 | 1.36E+08 | G | A | 0.657589 | 0.026259 | 0.007508 | 0.00047 | Age 56-60 |
| rs1129555 | 10 | 1.14E+08 | A | G | 0.274766 | 0.023281 | 0.007378 | 0.0016 | Age 61-65 |
| rs10128711 | 11 | 18632984 | T | C | 0.261951 | -0.02476 | 0.007449 | 0.00089 | Age 61-65 |
| rs149803 | 11 | 61539020 | C | G | 0.742539 | -0.0218 | 0.007642 | 0.0043 | Age 61-65 |
| rs499790 | 11 | 1.17E+08 | C | T | 0.897778 | -0.04666 | 0.010941 | 2.00E-05 | Age 61-65 |
| rs61905078 | 11 | 1.17E+08 | A | C | 0.927217 | -0.07581 | 0.012653 | 2.10E-09 | Age 61-65 |
| rs11220437 | 11 | 1.26E+08 | T | C | 0.885254 | -0.03212 | 0.010217 | 0.0017 | Age 61-65 |
| rs3093584 | 1 | 25668714 | T | C | 0.562201 | 0.020689 | 0.006564 | 0.0016 | Age 61-65 |
| rs12753981 | 1 | 27001893 | G | A | 0.915783 | -0.01219 | 0.01177 | 0.3 | Age 61-65 |
| rs2495504 | 1 | 55485796 | C | T | 0.281642 | 0.031443 | 0.007256 | 1.50E-05 | Age 61-65 |
| rs2479408 | 1 | 55504188 | C | G | 0.787173 | 0.042576 | 0.008131 | 1.60E-07 | Age 61-65 |
| rs2495477 | 1 | 55518467 | A | G | 0.607139 | 0.036 | 0.006861 | 1.50E-07 | Age 61-65 |
| rs626787 | 1 | 62901243 | C | G | 0.646712 | 0.048359 | 0.006873 | 2.00E-12 | Age 61-65 |
| rs1386585 | 1 | 92715207 | C | T | 0.19795 | -0.00949 | 0.008192 | 0.25 | Age 61-65 |
| rs1337247 | 1 | 1.1E+08 | C | A | 0.852933 | 0.027487 | 0.009202 | 0.0028 | Age 61-65 |
| rs4268379 | 1 | 1.1E+08 | C | T | 0.500198 | -0.03634 | 0.00656 | 3.00E-08 | Age 61-65 |
| rs267733 | 1 | 1.51E+08 | A | G | 0.840039 | 0.008423 | 0.008936 | 0.35 | Age 61-65 |
| rs2642438 | 1 | 2.21E+08 | A | G | 0.297568 | -0.0245 | 0.007157 | 0.00062 | Age 61-65 |
| rs487738 | 1 | 2.35E+08 | A | G | 0.325942 | -0.02496 | 0.007391 | 0.00073 | Age 61-65 |
| rs3184504 | 12 | 1.12E+08 | T | C | 0.484821 | -0.01997 | 0.006539 | 0.0023 | Age 61-65 |
| rs2649999 | 12 | 1.21E+08 | T | C | 0.327118 | 0.024101 | 0.007094 | 0.00068 | Age 61-65 |
| rs206070 | 13 | 32896846 | G | A | 0.821946 | 0.01235 | 0.008629 | 0.15 | Age 61-65 |
| rs6573778 | 14 | 24872209 | T | C | 0.481864 | 0.020855 | 0.006572 | 0.0015 | Age 61-65 |
| rs9989419 | 16 | 56985139 | A | G | 0.398945 | 0.02315 | 0.006705 | 0.00055 | Age 61-65 |
| rs9932707 | 16 | 71862133 | A | C | 0.538912 | -0.01681 | 0.006556 | 0.01 | Age 61-65 |
| rs4788597 | 16 | 72043039 | C | T | 0.637378 | 0.025918 | 0.00682 | 0.00014 | Age 61-65 |
| rs11650232 | 17 | 7088923 | A | G | 0.554291 | -0.01563 | 0.006585 | 0.018 | Age 61-65 |
| rs7225700 | 17 | 45391804 | T | C | 0.357794 | -0.01647 | 0.00682 | 0.016 | Age 61-65 |
| rs1801689 | 17 | 64210580 | A | C | 0.968492 | -0.07049 | 0.018792 | 0.00018 | Age 61-65 |
| rs2886232 | 17 | 67150176 | T | C | 0.123057 | 0.026316 | 0.01001 | 0.0086 | Age 61-65 |
| rs17677316 | 19 | 10680241 | G | A | 0.754426 | -0.01476 | 0.007561 | 0.051 | Age 61-65 |
| rs1529711 | 19 | 11023434 | C | T | 0.840698 | -0.02296 | 0.008902 | 0.0099 | Age 61-65 |
| rs17657025 | 19 | 44675801 | T | C | 0.91762 | 0.046485 | 0.011852 | 8.80E-05 | Age 61-65 |
| rs1004165 | 19 | 45232205 | G | A | 0.563092 | -0.03715 | 0.006578 | 1.60E-08 | Age 61-65 |
| rs11668536 | 19 | 45328476 | C | T | 0.755166 | 0.028623 | 0.00757 | 0.00016 | Age 61-65 |
| rs6857 | 19 | 45392254 | C | T | 0.833988 | -0.17683 | 0.008759 | 1.20E-90 | Age 61-65 |
| rs364585 | 20 | 12962718 | A | G | 0.391831 | -0.00828 | 0.006691 | 0.22 | Age 61-65 |
| rs2328223 | 20 | 17845921 | A | C | 0.816258 | -0.03513 | 0.008463 | 3.30E-05 | Age 61-65 |
| rs7264396 | 20 | 34154741 | C | T | 0.788269 | 0.027994 | 0.008038 | 5.00E-04 | Age 61-65 |
| rs6016373 | 20 | 39154095 | A | G | 0.592721 | 0.023408 | 0.006777 | 0.00055 | Age 61-65 |
| rs12624863 | 20 | 39782899 | A | G | 0.789151 | -0.0156 | 0.007996 | 0.051 | Age 61-65 |
| rs1800961 | 20 | 43042364 | C | T | 0.9684 | 0.053494 | 0.01869 | 0.0042 | Age 61-65 |
| rs13027716 | 2 | 20887226 | T | G | 0.969574 | 0.059738 | 0.019365 | 0.002 | Age 61-65 |
| rs11124924 | 2 | 21206275 | G | C | 0.937763 | 0.054246 | 0.013503 | 5.90E-05 | Age 61-65 |
| rs12720842 | 2 | 21257927 | T | C | 0.963448 | -0.04998 | 0.017383 | 0.004 | Age 61-65 |
| rs6756743 | 2 | 21301892 | C | T | 0.952823 | -0.0464 | 0.01551 | 0.0028 | Age 61-65 |
| rs10495907 | 2 | 43998726 | G | A | 0.866957 | -0.02998 | 0.009619 | 0.0018 | Age 61-65 |
| rs3923912 | 2 | 44057570 | A | C | 0.93829 | 0.106483 | 0.013577 | 4.40E-15 | Age 61-65 |
| rs4148217 | 2 | 44099433 | C | A | 0.81911 | 0.040338 | 0.008463 | 1.90E-06 | Age 61-65 |
| rs11125936 | 2 | 62871225 | T | C | 0.908175 | 0.031558 | 0.011293 | 0.0052 | Age 61-65 |
| rs17508045 | 2 | 1.19E+08 | T | C | 0.912476 | 0.015945 | 0.011541 | 0.17 | Age 61-65 |
| rs16831243 | 2 | 1.36E+08 | C | T | 0.899298 | -0.00596 | 0.0109 | 0.58 | Age 61-65 |
| rs10195252 | 2 | 1.66E+08 | T | C | 0.590522 | 0.010674 | 0.006646 | 0.11 | Age 61-65 |
| rs1250229 | 2 | 2.16E+08 | T | C | 0.261446 | -0.00805 | 0.007466 | 0.28 | Age 61-65 |
| rs11563251 | 2 | 2.35E+08 | C | T | 0.888949 | -0.02209 | 0.010369 | 0.033 | Age 61-65 |
| rs4823057 | 22 | 30241061 | A | G | 0.977555 | -0.00289 | 0.022182 | 0.9 | Age 61-65 |
| rs4253772 | 22 | 46627603 | C | T | 0.892506 | -0.01925 | 0.010594 | 0.069 | Age 61-65 |
| rs7616006 | 3 | 12267648 | A | G | 0.57737 | 0.005189 | 0.006638 | 0.43 | Age 61-65 |
| rs7640978 | 3 | 32533010 | C | T | 0.910705 | 0.062097 | 0.011454 | 5.90E-08 | Age 61-65 |
| rs17404153 | 3 | 1.32E+08 | G | T | 0.875596 | 0.021762 | 0.009975 | 0.029 | Age 61-65 |
| rs6818397 | 4 | 3434885 | T | G | 0.386783 | 0.027017 | 0.006781 | 6.80E-05 | Age 61-65 |
| rs4072296 | 5 | 74242002 | T | C | 0.457206 | -0.02075 | 0.006764 | 0.0022 | Age 61-65 |
| rs16872670 | 5 | 74929312 | G | A | 0.952512 | -0.02724 | 0.015388 | 0.077 | Age 61-65 |
| rs4530754 | 5 | 1.23E+08 | G | A | 0.452884 | -0.01699 | 0.006563 | 0.0096 | Age 61-65 |
| rs4704810 | 5 | 1.56E+08 | G | A | 0.626798 | -0.01278 | 0.006822 | 0.061 | Age 61-65 |
| rs2294266 | 6 | 16107867 | A | C | 0.50951 | 0.016031 | 0.006543 | 0.014 | Age 61-65 |
| rs1408272 | 6 | 25842951 | T | G | 0.921622 | 0.066515 | 0.012153 | 4.40E-08 | Age 61-65 |
| rs1049281 | 6 | 31236567 | T | C | 0.354405 | -0.02244 | 0.006882 | 0.0011 | Age 61-65 |
| rs3798236 | 6 | 1.16E+08 | T | C | 0.631738 | 0.018157 | 0.006817 | 0.0077 | Age 61-65 |
| rs6917747 | 6 | 1.6E+08 | G | A | 0.861856 | -0.03652 | 0.009481 | 0.00012 | Age 61-65 |
| rs9347386 | 6 | 1.61E+08 | A | G | 0.620402 | 0.021296 | 0.006804 | 0.0017 | Age 61-65 |
| rs6415084 | 6 | 1.61E+08 | T | C | 0.481034 | 0.021505 | 0.006554 | 0.001 | Age 61-65 |
| rs2390536 | 7 | 21485397 | G | A | 0.636809 | -0.02783 | 0.006832 | 4.60E-05 | Age 61-65 |
| rs4722551 | 7 | 25991826 | T | C | 0.842568 | -0.01981 | 0.00895 | 0.027 | Age 61-65 |
| rs7798185 | 7 | 44570717 | C | A | 0.826347 | -0.03738 | 0.00866 | 1.60E-05 | Age 61-65 |
| rs713286 | 8 | 9171735 | T | C | 0.92011 | 0.014154 | 0.012264 | 0.25 | Age 61-65 |
| rs10102164 | 8 | 55421614 | G | A | 0.787602 | -0.01754 | 0.007998 | 0.028 | Age 61-65 |
| rs2737229 | 8 | 1.17E+08 | A | C | 0.695548 | 0.020228 | 0.00711 | 0.0044 | Age 61-65 |
| rs17320616 | 8 | 1.26E+08 | A | G | 0.797111 | -0.03084 | 0.008212 | 0.00017 | Age 61-65 |
| rs7014582 | 8 | 1.45E+08 | A | G | 0.563449 | -0.01005 | 0.006608 | 0.13 | Age 61-65 |
| rs3780181 | 9 | 2640759 | A | G | 0.932375 | 0.019537 | 0.013082 | 0.14 | Age 61-65 |
| rs12686004 | 9 | 1.08E+08 | G | A | 0.883889 | 0.03092 | 0.010215 | 0.0025 | Age 61-65 |
| rs7849542 | 9 | 1.36E+08 | G | A | 0.696218 | -0.00412 | 0.007125 | 0.56 | Age 61-65 |
| rs7030248 | 9 | 1.36E+08 | G | A | 0.655663 | 0.013286 | 0.006897 | 0.054 | Age 61-65 |
| rs1129555 | 10 | 1.14E+08 | A | G | 0.277463 | 0.019058 | 0.009576 | 0.047 | Age >65 |
| rs10128711 | 11 | 18632984 | T | C | 0.261882 | -0.02175 | 0.009706 | 0.025 | Age >65 |
| rs149803 | 11 | 61539020 | C | G | 0.743546 | -0.00521 | 0.009938 | 0.6 | Age >65 |
| rs499790 | 11 | 1.17E+08 | C | T | 0.894154 | -0.03758 | 0.014177 | 0.008 | Age >65 |
| rs61905078 | 11 | 1.17E+08 | A | C | 0.926067 | -0.0811 | 0.016441 | 8.10E-07 | Age >65 |
| rs11220437 | 11 | 1.26E+08 | T | C | 0.884908 | 0.01466 | 0.013425 | 0.27 | Age >65 |
| rs3093584 | 1 | 25668714 | T | C | 0.560428 | 0.027449 | 0.008572 | 0.0014 | Age >65 |
| rs12753981 | 1 | 27001893 | G | A | 0.91565 | -0.03003 | 0.015297 | 0.05 | Age >65 |
| rs2495504 | 1 | 55485796 | C | T | 0.272391 | 0.028068 | 0.009593 | 0.0034 | Age >65 |
| rs2479408 | 1 | 55504188 | C | G | 0.792088 | 0.053877 | 0.010748 | 5.40E-07 | Age >65 |
| rs2495477 | 1 | 55518467 | A | G | 0.606679 | 0.036747 | 0.008889 | 3.60E-05 | Age >65 |
| rs626787 | 1 | 62901243 | C | G | 0.647859 | 0.030717 | 0.008965 | 0.00061 | Age >65 |
| rs1386585 | 1 | 92715207 | C | T | 0.195085 | -0.02022 | 0.010779 | 0.061 | Age >65 |
| rs1337247 | 1 | 1.1E+08 | C | A | 0.851684 | 0.023189 | 0.012028 | 0.054 | Age >65 |
| rs4268379 | 1 | 1.1E+08 | C | T | 0.497395 | -0.03196 | 0.008614 | 0.00021 | Age >65 |
| rs267733 | 1 | 1.51E+08 | A | G | 0.83716 | 0.002907 | 0.011609 | 0.8 | Age >65 |
| rs2642438 | 1 | 2.21E+08 | A | G | 0.300718 | -0.0054 | 0.009271 | 0.56 | Age >65 |
| rs487738 | 1 | 2.35E+08 | A | G | 0.32731 | -0.03091 | 0.009683 | 0.0014 | Age >65 |
| rs3184504 | 12 | 1.12E+08 | T | C | 0.480087 | -0.02768 | 0.008504 | 0.0011 | Age >65 |
| rs2649999 | 12 | 1.21E+08 | T | C | 0.333103 | 0.023044 | 0.009127 | 0.012 | Age >65 |
| rs206070 | 13 | 32896846 | G | A | 0.816937 | -0.02503 | 0.011062 | 0.024 | Age >65 |
| rs6573778 | 14 | 24872209 | T | C | 0.478658 | 0.021327 | 0.00864 | 0.014 | Age >65 |
| rs9989419 | 16 | 56985139 | A | G | 0.39242 | 0.010811 | 0.008784 | 0.22 | Age >65 |
| rs9932707 | 16 | 71862133 | A | C | 0.539677 | -0.02259 | 0.008575 | 0.0084 | Age >65 |
| rs4788597 | 16 | 72043039 | C | T | 0.63782 | 0.02966 | 0.008971 | 0.00095 | Age >65 |
| rs11650232 | 17 | 7088923 | A | G | 0.552658 | -0.01434 | 0.008591 | 0.095 | Age >65 |
| rs7225700 | 17 | 45391804 | T | C | 0.36013 | -0.02216 | 0.008998 | 0.014 | Age >65 |
| rs1801689 | 17 | 64210580 | A | C | 0.970457 | -0.01625 | 0.025335 | 0.52 | Age >65 |
| rs2886232 | 17 | 67150176 | T | C | 0.121948 | 0.031422 | 0.013095 | 0.016 | Age >65 |
| rs17677316 | 19 | 10680241 | G | A | 0.748205 | -0.02948 | 0.009795 | 0.0026 | Age >65 |
| rs1529711 | 19 | 11023434 | C | T | 0.84489 | -0.02067 | 0.011777 | 0.079 | Age >65 |
| rs17657025 | 19 | 44675801 | T | C | 0.918554 | 0.027603 | 0.015703 | 0.079 | Age >65 |
| rs1004165 | 19 | 45232205 | G | A | 0.55854 | -0.04468 | 0.008556 | 1.80E-07 | Age >65 |
| rs11668536 | 19 | 45328476 | C | T | 0.7517 | 0.008402 | 0.009946 | 0.4 | Age >65 |
| rs6857 | 19 | 45392254 | C | T | 0.83124 | -0.18072 | 0.011383 | 9.20E-57 | Age >65 |
| rs364585 | 20 | 12962718 | A | G | 0.388177 | -0.02245 | 0.00881 | 0.011 | Age >65 |
| rs2328223 | 20 | 17845921 | A | C | 0.815139 | -0.02062 | 0.011064 | 0.062 | Age >65 |
| rs7264396 | 20 | 34154741 | C | T | 0.790828 | 0.019181 | 0.010545 | 0.069 | Age >65 |
| rs6016373 | 20 | 39154095 | A | G | 0.595022 | 0.016769 | 0.00889 | 0.059 | Age >65 |
| rs12624863 | 20 | 39782899 | A | G | 0.791591 | -0.01301 | 0.010585 | 0.22 | Age >65 |
| rs1800961 | 20 | 43042364 | C | T | 0.968895 | 0.102443 | 0.024575 | 3.10E-05 | Age >65 |
| rs13027716 | 2 | 20887226 | T | G | 0.968691 | 0.028816 | 0.025007 | 0.25 | Age >65 |
| rs11124924 | 2 | 21206275 | G | C | 0.940535 | 0.058251 | 0.018168 | 0.0013 | Age >65 |
| rs12720842 | 2 | 21257927 | T | C | 0.962778 | -0.07599 | 0.022579 | 0.00076 | Age >65 |
| rs6756743 | 2 | 21301892 | C | T | 0.952915 | -0.06206 | 0.020384 | 0.0023 | Age >65 |
| rs10495907 | 2 | 43998726 | G | A | 0.86728 | -0.04172 | 0.012599 | 0.00093 | Age >65 |
| rs3923912 | 2 | 44057570 | A | C | 0.940226 | 0.117365 | 0.018062 | 8.20E-11 | Age >65 |
| rs4148217 | 2 | 44099433 | C | A | 0.817846 | 0.03306 | 0.011091 | 0.0029 | Age >65 |
| rs11125936 | 2 | 62871225 | T | C | 0.908532 | 0.024824 | 0.014854 | 0.095 | Age >65 |
| rs17508045 | 2 | 1.19E+08 | T | C | 0.912598 | 0.010828 | 0.015025 | 0.47 | Age >65 |
| rs16831243 | 2 | 1.36E+08 | C | T | 0.90152 | -0.01682 | 0.014387 | 0.24 | Age >65 |
| rs10195252 | 2 | 1.66E+08 | T | C | 0.594409 | 0.011416 | 0.008747 | 0.19 | Age >65 |
| rs1250229 | 2 | 2.16E+08 | T | C | 0.258603 | -0.01601 | 0.009858 | 0.1 | Age >65 |
| rs11563251 | 2 | 2.35E+08 | C | T | 0.888438 | -0.02727 | 0.013599 | 0.045 | Age >65 |
| rs4823057 | 22 | 30241061 | A | G | 0.977359 | 0.002549 | 0.028953 | 0.93 | Age >65 |
| rs4253772 | 22 | 46627603 | C | T | 0.889114 | -0.02537 | 0.01368 | 0.064 | Age >65 |
| rs7616006 | 3 | 12267648 | A | G | 0.580551 | 0.021351 | 0.008641 | 0.013 | Age >65 |
| rs7640978 | 3 | 32533010 | C | T | 0.912125 | 0.040279 | 0.015182 | 0.008 | Age >65 |
| rs17404153 | 3 | 1.32E+08 | G | T | 0.875635 | 0.017922 | 0.013061 | 0.17 | Age >65 |
| rs6818397 | 4 | 3434885 | T | G | 0.380479 | 0.0158 | 0.008898 | 0.076 | Age >65 |
| rs4072296 | 5 | 74242002 | T | C | 0.463624 | -0.03157 | 0.00889 | 0.00038 | Age >65 |
| rs16872670 | 5 | 74929312 | G | A | 0.951844 | -0.06334 | 0.019851 | 0.0014 | Age >65 |
| rs4530754 | 5 | 1.23E+08 | G | A | 0.458301 | -0.0022 | 0.008575 | 0.8 | Age >65 |
| rs4704810 | 5 | 1.56E+08 | G | A | 0.632251 | -0.01162 | 0.008942 | 0.19 | Age >65 |
| rs2294266 | 6 | 16107867 | A | C | 0.510208 | 0.019743 | 0.008646 | 0.022 | Age >65 |
| rs1408272 | 6 | 25842951 | T | G | 0.920792 | 0.063204 | 0.015865 | 6.80E-05 | Age >65 |
| rs1049281 | 6 | 31236567 | T | C | 0.358608 | -0.01376 | 0.008915 | 0.12 | Age >65 |
| rs3798236 | 6 | 1.16E+08 | T | C | 0.627705 | -0.0051 | 0.008872 | 0.57 | Age >65 |
| rs6917747 | 6 | 1.6E+08 | G | A | 0.862877 | -0.05053 | 0.012517 | 5.40E-05 | Age >65 |
| rs9347386 | 6 | 1.61E+08 | A | G | 0.620891 | 0.032784 | 0.008842 | 0.00021 | Age >65 |
| rs6415084 | 6 | 1.61E+08 | T | C | 0.477458 | 0.017978 | 0.008533 | 0.035 | Age >65 |
| rs2390536 | 7 | 21485397 | G | A | 0.636433 | -0.0388 | 0.008895 | 1.30E-05 | Age >65 |
| rs4722551 | 7 | 25991826 | T | C | 0.842704 | -0.02345 | 0.011736 | 0.046 | Age >65 |
| rs7798185 | 7 | 44570717 | C | A | 0.825889 | -0.02266 | 0.011312 | 0.045 | Age >65 |
| rs713286 | 8 | 9171735 | T | C | 0.919391 | -0.00249 | 0.015904 | 0.88 | Age >65 |
| rs10102164 | 8 | 55421614 | G | A | 0.784554 | -0.02834 | 0.010408 | 0.0065 | Age >65 |
| rs2737229 | 8 | 1.17E+08 | A | C | 0.694362 | 0.02695 | 0.00935 | 0.0039 | Age >65 |
| rs17320616 | 8 | 1.26E+08 | A | G | 0.795955 | -0.02271 | 0.010732 | 0.034 | Age >65 |
| rs7014582 | 8 | 1.45E+08 | A | G | 0.561942 | -0.0169 | 0.008657 | 0.051 | Age >65 |
| rs3780181 | 9 | 2640759 | A | G | 0.934373 | 0.032024 | 0.017397 | 0.066 | Age >65 |
| rs12686004 | 9 | 1.08E+08 | G | A | 0.883076 | 0.021573 | 0.013329 | 0.11 | Age >65 |
| rs7849542 | 9 | 1.36E+08 | G | A | 0.69953 | -0.02082 | 0.009413 | 0.027 | Age >65 |
| rs7030248 | 9 | 1.36E+08 | G | A | 0.651512 | 0.000504 | 0.009006 | 0.96 | Age >65 |

**Supplemental Table 3.** Genetic instruments used for SBP for analyses of incident CHD

| SNP | CHR | BP | ALLELE1 | ALLELE0 | A1FREQ | BETA | SE | P | phenotype |
| --- | --- | --- | --- | --- | --- | --- | --- | --- | --- |
| rs1623474 | 10 | 18471794 | C | T | 0.669691 | -0.28072 | 0.104556 | 0.0073 | Age ≤55 |
| rs12258967 | 10 | 18727959 | C | G | 0.700794 | 0.730215 | 0.107298 | 1.00E-11 | Age ≤55 |
| rs57946343 | 10 | 63499951 | T | C | 0.848709 | 0.627585 | 0.136873 | 4.50E-06 | Age ≤55 |
| rs10128354 | 10 | 75470045 | T | C | 0.412354 | 0.156039 | 0.099316 | 0.12 | Age ≤55 |
| rs10882398 | 10 | 95892788 | T | A | 0.564517 | -0.52183 | 0.099195 | 1.40E-07 | Age ≤55 |
| rs1006545 | 10 | 1.03E+08 | G | T | 0.111871 | -0.59941 | 0.15551 | 0.00012 | Age ≤55 |
| rs11191551 | 10 | 1.05E+08 | A | G | 0.922905 | 1.03825 | 0.183479 | 1.50E-08 | Age ≤55 |
| rs1801253 | 10 | 1.16E+08 | G | C | 0.265507 | -0.50277 | 0.111424 | 6.40E-06 | Age ≤55 |
| rs11592107 | 10 | 1.23E+08 | G | A | 0.689801 | -0.48891 | 0.106199 | 4.20E-06 | Age ≤55 |
| rs592373 | 11 | 1890990 | G | A | 0.371118 | -0.5277 | 0.101541 | 2.00E-07 | Age ≤55 |
| rs2218305 | 11 | 10247896 | G | C | 0.505986 | -0.18565 | 0.098225 | 0.059 | Age ≤55 |
| rs1966698 | 11 | 16306267 | C | T | 0.206833 | 0.4294 | 0.121096 | 0.00039 | Age ≤55 |
| rs416258 | 11 | 16911636 | G | C | 0.695198 | -0.28635 | 0.10631 | 0.0071 | Age ≤55 |
| rs7107356 | 11 | 47676170 | A | G | 0.491667 | -0.32825 | 0.098062 | 0.00082 | Age ≤55 |
| rs10750766 | 11 | 65473798 | C | A | 0.28985 | -0.28516 | 0.108026 | 0.0083 | Age ≤55 |
| rs139437879 | 11 | 67904333 | G | A | 0.984524 | 0.027015 | 0.404875 | 0.95 | Age ≤55 |
| rs58941166 | 11 | 72083441 | G | A | 0.933818 | -0.21406 | 0.19728 | 0.28 | Age ≤55 |
| rs604723 | 11 | 1.01E+08 | T | C | 0.273457 | -0.84583 | 0.110395 | 1.80E-14 | Age ≤55 |
| rs2072927 | 1 | 1684800 | G | A | 0.499677 | 0.40927 | 0.097896 | 2.90E-05 | Age ≤55 |
| rs488834 | 1 | 10767902 | C | T | 0.232922 | 0.225894 | 0.115364 | 0.05 | Age ≤55 |
| rs6669371 | 1 | 11882142 | T | G | 0.836431 | 0.864351 | 0.132434 | 6.70E-11 | Age ≤55 |
| rs75461554 | 1 | 15810172 | C | T | 0.802543 | 0.158556 | 0.122896 | 0.2 | Age ≤55 |
| rs4908348 | 1 | 28706949 | T | G | 0.693389 | 0.307335 | 0.106447 | 0.0039 | Age ≤55 |
| rs1408945 | 1 | 42364877 | G | T | 0.571901 | 0.292467 | 0.098851 | 0.0031 | Age ≤55 |
| rs12132894 | 1 | 89138474 | A | T | 0.585443 | 0.076856 | 0.099878 | 0.44 | Age ≤55 |
| rs10776752 | 1 | 1.13E+08 | G | T | 0.926731 | -0.65878 | 0.188042 | 0.00046 | Age ≤55 |
| rs10914117 | 1 | 1.81E+08 | T | C | 0.59298 | 0.140409 | 0.099594 | 0.16 | Age ≤55 |
| rs68085857 | 1 | 2.18E+08 | C | T | 0.76692 | -0.09292 | 0.11537 | 0.42 | Age ≤55 |
| rs1745417 | 1 | 2.28E+08 | C | T | 0.487814 | -0.35155 | 0.098019 | 0.00034 | Age ≤55 |
| rs699 | 1 | 2.31E+08 | A | G | 0.59553 | -0.31239 | 0.099777 | 0.0017 | Age ≤55 |
| rs3213751 | 12 | 432666 | G | C | 0.74092 | -0.27914 | 0.111598 | 0.012 | Age ≤55 |
| rs11055034 | 12 | 12890626 | C | A | 0.717939 | 0.231099 | 0.109075 | 0.034 | Age ≤55 |
| rs1010064 | 12 | 20000315 | A | C | 0.813921 | 0.235355 | 0.125825 | 0.061 | Age ≤55 |
| rs73075659 | 12 | 20373541 | A | G | 0.654266 | 0.332079 | 0.103217 | 0.0013 | Age ≤55 |
| rs150857355 | 12 | 49209340 | G | C | 0.978167 | -1.09409 | 0.342207 | 0.0014 | Age ≤55 |
| rs7967954 | 12 | 50673484 | G | A | 0.420704 | 0.271514 | 0.099198 | 0.0062 | Age ≤55 |
| rs736825 | 12 | 54417576 | C | G | 0.636078 | 0.334913 | 0.10219 | 0.001 | Age ≤55 |
| rs4143175 | 12 | 67782397 | T | C | 0.240668 | 0.222706 | 0.115543 | 0.054 | Age ≤55 |
| rs17249754 | 12 | 90060586 | G | A | 0.828249 | 0.710608 | 0.130092 | 4.70E-08 | Age ≤55 |
| rs7310615 | 12 | 1.12E+08 | C | G | 0.479486 | 0.605458 | 0.098679 | 8.50E-10 | Age ≤55 |
| rs35443 | 12 | 1.16E+08 | G | C | 0.622041 | 0.438145 | 0.100546 | 1.30E-05 | Age ≤55 |
| rs6490021 | 12 | 1.16E+08 | A | C | 0.384401 | -0.24819 | 0.100616 | 0.014 | Age ≤55 |
| rs9508495 | 13 | 30146201 | C | T | 0.236602 | 0.207256 | 0.115166 | 0.072 | Age ≤55 |
| rs4415898 | 13 | 41895177 | T | A | 0.978202 | 0.065214 | 0.378418 | 0.86 | Age ≤55 |
| rs4415898 | 13 | 41895177 | T | G | 0.166847 | -0.06069 | 0.131433 | 0.64 | Age ≤55 |
| rs8904 | 14 | 35871217 | G | A | 0.635884 | -0.3256 | 0.101709 | 0.0014 | Age ≤55 |
| rs28365937 | 14 | 53418233 | C | A | 0.695176 | -0.34972 | 0.106735 | 0.0011 | Age ≤55 |
| rs12591513 | 15 | 75102714 | G | A | 0.331153 | 0.597794 | 0.104164 | 9.50E-09 | Age ≤55 |
| rs2627316 | 15 | 81042816 | A | G | 0.531224 | -0.20635 | 0.098422 | 0.036 | Age ≤55 |
| rs4932373 | 15 | 91429287 | A | C | 0.673407 | -0.64049 | 0.104791 | 9.80E-10 | Age ≤55 |
| rs10500326 | 16 | 4918326 | G | T | 0.764944 | 0.179437 | 0.115666 | 0.12 | Age ≤55 |
| rs4293393 | 16 | 20364588 | A | G | 0.817069 | 0.278778 | 0.126822 | 0.028 | Age ≤55 |
| rs4888408 | 16 | 75432824 | G | A | 0.403395 | -0.49402 | 0.099697 | 7.20E-07 | Age ≤55 |
| rs9806885 | 16 | 81556909 | A | G | 0.889773 | 0.257467 | 0.156115 | 0.099 | Age ≤55 |
| rs908951 | 16 | 89697625 | C | T | 0.57063 | 0.268297 | 0.099586 | 0.0071 | Age ≤55 |
| rs222857 | 17 | 7164563 | C | T | 0.433957 | -0.08744 | 0.098865 | 0.38 | Age ≤55 |
| rs7213273 | 17 | 43155914 | G | A | 0.347167 | 0.493133 | 0.102882 | 1.60E-06 | Age ≤55 |
| rs17608766 | 17 | 45013271 | T | C | 0.853368 | -0.5497 | 0.138362 | 7.10E-05 | Age ≤55 |
| rs7406910 | 17 | 46688256 | T | C | 0.089217 | -0.43832 | 0.171718 | 0.011 | Age ≤55 |
| rs2671661 | 17 | 47528562 | T | C | 0.271273 | -0.2374 | 0.11184 | 0.034 | Age ≤55 |
| rs1000423 | 17 | 59475642 | C | T | 0.265467 | -0.23781 | 0.111391 | 0.033 | Age ≤55 |
| rs77946446 | 17 | 75317932 | C | T | 0.734497 | 0.250473 | 0.111641 | 0.025 | Age ≤55 |
| rs12955644 | 18 | 771047 | C | G | 0.815914 | 0.474484 | 0.127021 | 0.00019 | Age ≤55 |
| rs56407827 | 18 | 42179819 | C | T | 0.730226 | -0.40929 | 0.110993 | 0.00023 | Age ≤55 |
| rs76978865 | 19 | 2175799 | G | A | 0.960305 | -1.01775 | 0.251219 | 5.10E-05 | Age ≤55 |
| rs10427021 | 19 | 7259346 | T | G | 0.889696 | 0.76237 | 0.172633 | 1.00E-05 | Age ≤55 |
| rs79709447 | 19 | 12187621 | T | C | 0.987166 | -0.05861 | 0.445263 | 0.9 | Age ≤55 |
| rs1327235 | 20 | 10969030 | A | G | 0.524378 | -0.47778 | 0.098293 | 1.20E-06 | Age ≤55 |
| rs17812022 | 20 | 19007099 | C | T | 0.905616 | -0.11256 | 0.167716 | 0.5 | Age ≤55 |
| rs6102506 | 20 | 40254735 | C | T | 0.650523 | 0.140212 | 0.103783 | 0.18 | Age ≤55 |
| rs6026744 | 20 | 57742388 | A | T | 0.880547 | -0.39853 | 0.152404 | 0.0089 | Age ≤55 |
| rs1275988 | 2 | 26914364 | C | T | 0.384527 | 0.389543 | 0.100867 | 0.00011 | Age ≤55 |
| rs35441226 | 2 | 43191137 | C | T | 0.817108 | 0.368832 | 0.127498 | 0.0038 | Age ≤55 |
| rs56308618 | 21 | 44757792 | G | A | 0.708939 | -0.43315 | 0.108399 | 6.40E-05 | Age ≤55 |
| rs58117425 | 2 | 1.46E+08 | G | A | 0.767118 | -0.21877 | 0.115895 | 0.059 | Age ≤55 |
| rs73029563 | 2 | 1.65E+08 | C | G | 0.458379 | -0.49338 | 0.098334 | 5.20E-07 | Age ≤55 |
| rs10205122 | 2 | 1.81E+08 | A | G | 0.71799 | 0.103727 | 0.109316 | 0.34 | Age ≤55 |
| rs7592578 | 2 | 1.91E+08 | T | G | 0.185283 | -0.29265 | 0.126483 | 0.021 | Age ≤55 |
| rs3845811 | 2 | 2.09E+08 | C | G | 0.566257 | -0.21522 | 0.099079 | 0.03 | Age ≤55 |
| rs2161967 | 2 | 2.19E+08 | T | G | 0.424056 | 0.336741 | 0.099634 | 0.00073 | Age ≤55 |
| rs2594992 | 3 | 11360997 | A | C | 0.597373 | -0.1408 | 0.099487 | 0.16 | Age ≤55 |
| rs34991912 | 3 | 14926351 | T | C | 0.416156 | 0.372765 | 0.099565 | 0.00018 | Age ≤55 |
| rs2643826 | 3 | 27562988 | C | T | 0.550659 | -0.46065 | 0.09871 | 3.10E-06 | Age ≤55 |
| rs6771917 | 3 | 48108442 | T | C | 0.244323 | -0.47504 | 0.114107 | 3.10E-05 | Age ≤55 |
| rs3772219 | 3 | 56771251 | A | C | 0.674705 | 0.375111 | 0.104517 | 0.00033 | Age ≤55 |
| rs73872710 | 3 | 1.41E+08 | A | T | 0.924455 | 0.02842 | 0.185404 | 0.88 | Age ≤55 |
| rs1597467 | 3 | 1.5E+08 | C | A | 0.905624 | -0.30621 | 0.167982 | 0.068 | Age ≤55 |
| rs11715377 | 3 | 1.69E+08 | A | T | 0.906134 | 0.704435 | 0.16871 | 3.00E-05 | Age ≤55 |
| rs1528293 | 3 | 1.69E+08 | A | T | 0.489985 | 0.424766 | 0.097517 | 1.30E-05 | Age ≤55 |
| rs16852391 | 4 | 40604309 | C | G | 0.893366 | 0.009627 | 0.158897 | 0.95 | Age ≤55 |
| rs12509595 | 4 | 81182554 | T | C | 0.706597 | -1.18857 | 0.107859 | 3.10E-28 | Age ≤55 |
| rs17010957 | 4 | 86719165 | T | C | 0.851854 | -0.6496 | 0.139545 | 3.20E-06 | Age ≤55 |
| rs13135092 | 4 | 1.03E+08 | A | G | 0.916262 | 0.293921 | 0.17758 | 0.098 | Age ≤55 |
| rs6820343 | 4 | 1.56E+08 | C | T | 0.466865 | 0.375474 | 0.098568 | 0.00014 | Age ≤55 |
| rs72689147 | 4 | 1.57E+08 | G | T | 0.817263 | 0.467073 | 0.126722 | 0.00023 | Age ≤55 |
| rs10069690 | 5 | 1279790 | C | T | 0.743925 | -0.30672 | 0.111811 | 0.0061 | Age ≤55 |
| rs7733331 | 5 | 32828846 | T | C | 0.401511 | -0.77805 | 0.099869 | 6.70E-15 | Age ≤55 |
| rs2112453 | 5 | 1.12E+08 | G | A | 0.265577 | 0.214068 | 0.110645 | 0.053 | Age ≤55 |
| rs4487481 | 5 | 1.14E+08 | T | C | 0.481266 | 0.186863 | 0.097995 | 0.057 | Age ≤55 |
| rs6890251 | 5 | 1.22E+08 | C | T | 0.848833 | -0.6084 | 0.136826 | 8.70E-06 | Age ≤55 |
| rs7703751 | 5 | 1.23E+08 | A | T | 0.741053 | 0.249058 | 0.111732 | 0.026 | Age ≤55 |
| rs6892983 | 5 | 1.28E+08 | C | A | 0.590824 | -0.22868 | 0.099555 | 0.022 | Age ≤55 |
| rs7737361 | 5 | 1.48E+08 | G | A | 0.804764 | 0.339871 | 0.126168 | 0.0071 | Age ≤55 |
| rs1650581 | 5 | 1.58E+08 | C | G | 0.739321 | -0.30603 | 0.111843 | 0.0062 | Age ≤55 |
| rs12187017 | 5 | 1.58E+08 | G | A | 0.638999 | 0.236289 | 0.10195 | 0.02 | Age ≤55 |
| rs198851 | 6 | 26104632 | T | G | 0.149589 | 0.437516 | 0.137187 | 0.0014 | Age ≤55 |
| rs1265157 | 6 | 31142265 | C | G | 0.648304 | 0.077549 | 0.102657 | 0.45 | Age ≤55 |
| rs2856448 | 6 | 32014575 | A | G | 0.535634 | -0.42427 | 0.098226 | 1.60E-05 | Age ≤55 |
| rs1214761 | 6 | 43354431 | A | G | 0.323561 | 0.280923 | 0.104808 | 0.0074 | Age ≤55 |
| rs211159 | 6 | 97023026 | G | A | 0.658575 | -0.15725 | 0.103501 | 0.13 | Age ≤55 |
| rs4897160 | 6 | 1.26E+08 | G | A | 0.524043 | -0.1539 | 0.09801 | 0.12 | Age ≤55 |
| rs9375459 | 6 | 1.27E+08 | C | T | 0.563472 | -0.49324 | 0.099188 | 6.60E-07 | Age ≤55 |
| rs62434129 | 6 | 1.51E+08 | A | T | 0.928902 | 0.797582 | 0.190592 | 2.90E-05 | Age ≤55 |
| rs2969036 | 7 | 2534901 | T | G | 0.310141 | 0.473903 | 0.105973 | 7.80E-06 | Age ≤55 |
| rs3735533 | 7 | 27245893 | T | C | 0.072747 | -0.56033 | 0.188589 | 0.003 | Age ≤55 |
| rs6961048 | 7 | 27328187 | C | G | 0.897386 | -0.57751 | 0.161491 | 0.00035 | Age ≤55 |
| rs2392929 | 7 | 1.06E+08 | T | G | 0.802054 | -0.60044 | 0.122562 | 9.60E-07 | Age ≤55 |
| rs3807306 | 7 | 1.29E+08 | G | T | 0.507392 | 0.050308 | 0.098095 | 0.61 | Age ≤55 |
| rs3918226 | 7 | 1.51E+08 | C | T | 0.918315 | -0.56644 | 0.182402 | 0.0019 | Age ≤55 |
| rs1991651 | 8 | 10706411 | C | G | 0.381211 | 0.479043 | 0.101067 | 2.10E-06 | Age ≤55 |
| rs1352026 | 8 | 25895899 | C | T | 0.745691 | 0.404501 | 0.112487 | 0.00032 | Age ≤55 |
| rs830444 | 8 | 76954113 | G | T | 0.397783 | -0.36594 | 0.10035 | 0.00027 | Age ≤55 |
| rs35783704 | 8 | 1.06E+08 | G | A | 0.899238 | 0.462001 | 0.164093 | 0.0049 | Age ≤55 |
| rs62521878 | 8 | 1.42E+08 | C | T | 0.703742 | -0.18072 | 0.106727 | 0.09 | Age ≤55 |
| rs72762681 | 9 | 1.13E+08 | A | T | 0.849964 | -0.12247 | 0.137269 | 0.37 | Age ≤55 |
| rs1623474 | 10 | 18471794 | C | T | 0.66865 | -0.43172 | 0.176571 | 0.014 | Age 56-60 |
| rs12258967 | 10 | 18727959 | C | G | 0.698188 | 0.802471 | 0.179772 | 8.10E-06 | Age 56-60 |
| rs57946343 | 10 | 63499951 | T | C | 0.847303 | 1.1179 | 0.229291 | 1.10E-06 | Age 56-60 |
| rs10128354 | 10 | 75470045 | T | C | 0.412175 | 0.433537 | 0.167259 | 0.0095 | Age 56-60 |
| rs10882398 | 10 | 95892788 | T | A | 0.565278 | -0.30623 | 0.167684 | 0.068 | Age 56-60 |
| rs1006545 | 10 | 1.03E+08 | G | T | 0.112173 | -0.94289 | 0.262184 | 0.00032 | Age 56-60 |
| rs11191551 | 10 | 1.05E+08 | A | G | 0.924016 | 1.44972 | 0.312229 | 3.40E-06 | Age 56-60 |
| rs1801253 | 10 | 1.16E+08 | G | C | 0.265628 | -0.92647 | 0.187498 | 7.80E-07 | Age 56-60 |
| rs11592107 | 10 | 1.23E+08 | G | A | 0.691616 | -0.08164 | 0.178533 | 0.65 | Age 56-60 |
| rs592373 | 11 | 1890990 | G | A | 0.367465 | -0.51749 | 0.171187 | 0.0025 | Age 56-60 |
| rs2218305 | 11 | 10247896 | G | C | 0.507334 | -0.54588 | 0.164419 | 9.00E-04 | Age 56-60 |
| rs1966698 | 11 | 16306267 | C | T | 0.20669 | 0.485574 | 0.203005 | 0.017 | Age 56-60 |
| rs416258 | 11 | 16911636 | G | C | 0.69183 | -0.26652 | 0.178554 | 0.14 | Age 56-60 |
| rs7107356 | 11 | 47676170 | A | G | 0.490041 | -0.61608 | 0.165 | 0.00019 | Age 56-60 |
| rs10750766 | 11 | 65473798 | C | A | 0.295628 | -0.47931 | 0.181375 | 0.0082 | Age 56-60 |
| rs139437879 | 11 | 67904333 | G | A | 0.984334 | 0.803043 | 0.679763 | 0.24 | Age 56-60 |
| rs58941166 | 11 | 72083441 | G | A | 0.933662 | -0.38334 | 0.331675 | 0.25 | Age 56-60 |
| rs604723 | 11 | 1.01E+08 | T | C | 0.274845 | -0.55302 | 0.185999 | 0.0029 | Age 56-60 |
| rs2072927 | 1 | 1684800 | G | A | 0.502227 | 0.283023 | 0.164481 | 0.085 | Age 56-60 |
| rs488834 | 1 | 10767902 | C | T | 0.230276 | 0.531581 | 0.195485 | 0.0065 | Age 56-60 |
| rs6669371 | 1 | 11882142 | T | G | 0.836408 | 0.676107 | 0.223137 | 0.0024 | Age 56-60 |
| rs75461554 | 1 | 15810172 | C | T | 0.80436 | 0.481403 | 0.20771 | 0.02 | Age 56-60 |
| rs4908348 | 1 | 28706949 | T | G | 0.697671 | 0.054911 | 0.180069 | 0.76 | Age 56-60 |
| rs1408945 | 1 | 42364877 | G | T | 0.577517 | -0.08002 | 0.167096 | 0.63 | Age 56-60 |
| rs12132894 | 1 | 89138474 | A | T | 0.586489 | 0.400547 | 0.168242 | 0.017 | Age 56-60 |
| rs10776752 | 1 | 1.13E+08 | G | T | 0.92526 | -1.13384 | 0.313097 | 0.00029 | Age 56-60 |
| rs10914117 | 1 | 1.81E+08 | T | C | 0.594174 | 0.213228 | 0.168648 | 0.21 | Age 56-60 |
| rs68085857 | 1 | 2.18E+08 | C | T | 0.769119 | -0.00939 | 0.194898 | 0.96 | Age 56-60 |
| rs1745417 | 1 | 2.28E+08 | C | T | 0.485647 | -0.45705 | 0.165191 | 0.0057 | Age 56-60 |
| rs699 | 1 | 2.31E+08 | A | G | 0.595247 | -0.26017 | 0.16817 | 0.12 | Age 56-60 |
| rs3213751 | 12 | 432666 | G | C | 0.740431 | -0.06151 | 0.188672 | 0.74 | Age 56-60 |
| rs11055034 | 12 | 12890626 | C | A | 0.721843 | 0.212638 | 0.184188 | 0.25 | Age 56-60 |
| rs1010064 | 12 | 20000315 | A | C | 0.815729 | 0.547883 | 0.213075 | 0.01 | Age 56-60 |
| rs73075659 | 12 | 20373541 | A | G | 0.650991 | 0.155583 | 0.174308 | 0.37 | Age 56-60 |
| rs150857355 | 12 | 49209340 | G | C | 0.978751 | -1.396 | 0.581446 | 0.016 | Age 56-60 |
| rs7967954 | 12 | 50673484 | G | A | 0.421597 | 0.443286 | 0.16657 | 0.0078 | Age 56-60 |
| rs736825 | 12 | 54417576 | C | G | 0.639873 | 0.298084 | 0.17253 | 0.084 | Age 56-60 |
| rs4143175 | 12 | 67782397 | T | C | 0.240731 | 0.343093 | 0.193733 | 0.077 | Age 56-60 |
| rs17249754 | 12 | 90060586 | G | A | 0.827797 | 0.648585 | 0.218462 | 0.003 | Age 56-60 |
| rs7310615 | 12 | 1.12E+08 | C | G | 0.482896 | 0.381605 | 0.165893 | 0.021 | Age 56-60 |
| rs35443 | 12 | 1.16E+08 | G | C | 0.618035 | 0.57891 | 0.169854 | 0.00065 | Age 56-60 |
| rs6490021 | 12 | 1.16E+08 | A | C | 0.380536 | -0.46895 | 0.170096 | 0.0058 | Age 56-60 |
| rs9508495 | 13 | 30146201 | C | T | 0.238033 | 0.59564 | 0.194716 | 0.0022 | Age 56-60 |
| rs4415898 | 13 | 41895177 | T | A | 0.978516 | -0.84377 | 0.640659 | 0.19 | Age 56-60 |
| rs4415898 | 13 | 41895177 | T | G | 0.170731 | -0.38577 | 0.219573 | 0.079 | Age 56-60 |
| rs8904 | 14 | 35871217 | G | A | 0.638913 | -0.78789 | 0.171532 | 4.40E-06 | Age 56-60 |
| rs28365937 | 14 | 53418233 | C | A | 0.696304 | -0.07222 | 0.18041 | 0.69 | Age 56-60 |
| rs12591513 | 15 | 75102714 | G | A | 0.33325 | 0.466547 | 0.174027 | 0.0073 | Age 56-60 |
| rs2627316 | 15 | 81042816 | A | G | 0.526823 | -0.48678 | 0.16641 | 0.0034 | Age 56-60 |
| rs4932373 | 15 | 91429287 | A | C | 0.672038 | -1.07425 | 0.174699 | 7.80E-10 | Age 56-60 |
| rs10500326 | 16 | 4918326 | G | T | 0.759452 | 0.29709 | 0.193466 | 0.12 | Age 56-60 |
| rs4293393 | 16 | 20364588 | A | G | 0.816275 | 0.40658 | 0.212074 | 0.055 | Age 56-60 |
| rs4888408 | 16 | 75432824 | G | A | 0.404602 | -0.39923 | 0.16837 | 0.018 | Age 56-60 |
| rs9806885 | 16 | 81556909 | A | G | 0.887091 | 0.145229 | 0.261454 | 0.58 | Age 56-60 |
| rs908951 | 16 | 89697625 | C | T | 0.57123 | 0.461698 | 0.167824 | 0.0059 | Age 56-60 |
| rs222857 | 17 | 7164563 | C | T | 0.43062 | -0.20288 | 0.166313 | 0.22 | Age 56-60 |
| rs7213273 | 17 | 43155914 | G | A | 0.347578 | 0.666177 | 0.173283 | 0.00012 | Age 56-60 |
| rs17608766 | 17 | 45013271 | T | C | 0.857093 | -0.62846 | 0.235776 | 0.0077 | Age 56-60 |
| rs7406910 | 17 | 46688256 | T | C | 0.086166 | -0.08444 | 0.292736 | 0.77 | Age 56-60 |
| rs2671661 | 17 | 47528562 | T | C | 0.272814 | -0.36185 | 0.187854 | 0.054 | Age 56-60 |
| rs1000423 | 17 | 59475642 | C | T | 0.267633 | -0.60001 | 0.186066 | 0.0013 | Age 56-60 |
| rs77946446 | 17 | 75317932 | C | T | 0.735457 | 0.207754 | 0.188323 | 0.27 | Age 56-60 |
| rs12955644 | 18 | 771047 | C | G | 0.815528 | 0.443612 | 0.215018 | 0.039 | Age 56-60 |
| rs56407827 | 18 | 42179819 | C | T | 0.733042 | -0.33533 | 0.186689 | 0.072 | Age 56-60 |
| rs76978865 | 19 | 2175799 | G | A | 0.959238 | -0.79499 | 0.418818 | 0.058 | Age 56-60 |
| rs10427021 | 19 | 7259346 | T | G | 0.890716 | 1.35838 | 0.292514 | 3.40E-06 | Age 56-60 |
| rs79709447 | 19 | 12187621 | T | C | 0.988097 | 0.283714 | 0.777172 | 0.72 | Age 56-60 |
| rs1327235 | 20 | 10969030 | A | G | 0.526355 | -0.37096 | 0.165376 | 0.025 | Age 56-60 |
| rs17812022 | 20 | 19007099 | C | T | 0.906383 | 0.179515 | 0.284915 | 0.53 | Age 56-60 |
| rs6102506 | 20 | 40254735 | C | T | 0.651539 | -0.22722 | 0.174589 | 0.19 | Age 56-60 |
| rs6026744 | 20 | 57742388 | A | T | 0.881122 | -1.20475 | 0.256859 | 2.70E-06 | Age 56-60 |
| rs1275988 | 2 | 26914364 | C | T | 0.383528 | 0.943558 | 0.170202 | 3.00E-08 | Age 56-60 |
| rs35441226 | 2 | 43191137 | C | T | 0.817776 | 0.272842 | 0.213859 | 0.2 | Age 56-60 |
| rs56308618 | 21 | 44757792 | G | A | 0.709159 | -0.38996 | 0.182403 | 0.033 | Age 56-60 |
| rs58117425 | 2 | 1.46E+08 | G | A | 0.760793 | -0.46572 | 0.194308 | 0.017 | Age 56-60 |
| rs73029563 | 2 | 1.65E+08 | C | G | 0.455172 | -0.27029 | 0.166167 | 0.1 | Age 56-60 |
| rs10205122 | 2 | 1.81E+08 | A | G | 0.716488 | 0.511817 | 0.183473 | 0.0053 | Age 56-60 |
| rs7592578 | 2 | 1.91E+08 | T | G | 0.185318 | -0.5879 | 0.213238 | 0.0058 | Age 56-60 |
| rs3845811 | 2 | 2.09E+08 | C | G | 0.565261 | -0.3154 | 0.165342 | 0.056 | Age 56-60 |
| rs2161967 | 2 | 2.19E+08 | T | G | 0.424929 | 0.448488 | 0.167161 | 0.0073 | Age 56-60 |
| rs2594992 | 3 | 11360997 | A | C | 0.59656 | -0.27057 | 0.168396 | 0.11 | Age 56-60 |
| rs34991912 | 3 | 14926351 | T | C | 0.415514 | 0.400354 | 0.167655 | 0.017 | Age 56-60 |
| rs2643826 | 3 | 27562988 | C | T | 0.552259 | -0.68771 | 0.166894 | 3.80E-05 | Age 56-60 |
| rs6771917 | 3 | 48108442 | T | C | 0.24354 | -0.31983 | 0.192608 | 0.097 | Age 56-60 |
| rs3772219 | 3 | 56771251 | A | C | 0.674884 | 0.42928 | 0.176404 | 0.015 | Age 56-60 |
| rs73872710 | 3 | 1.41E+08 | A | T | 0.923407 | 0.08992 | 0.312805 | 0.77 | Age 56-60 |
| rs1597467 | 3 | 1.5E+08 | C | A | 0.906383 | -0.68258 | 0.280653 | 0.015 | Age 56-60 |
| rs11715377 | 3 | 1.69E+08 | A | T | 0.904729 | 0.272907 | 0.281052 | 0.33 | Age 56-60 |
| rs1528293 | 3 | 1.69E+08 | A | T | 0.488932 | 0.425966 | 0.165597 | 0.01 | Age 56-60 |
| rs16852391 | 4 | 40604309 | C | G | 0.895601 | -0.00867 | 0.268847 | 0.97 | Age 56-60 |
| rs12509595 | 4 | 81182554 | T | C | 0.708466 | -1.00414 | 0.181557 | 3.20E-08 | Age 56-60 |
| rs17010957 | 4 | 86719165 | T | C | 0.85147 | -0.09845 | 0.234446 | 0.67 | Age 56-60 |
| rs13135092 | 4 | 1.03E+08 | A | G | 0.916829 | 1.09788 | 0.300148 | 0.00025 | Age 56-60 |
| rs6820343 | 4 | 1.56E+08 | C | T | 0.463394 | 0.394941 | 0.164943 | 0.017 | Age 56-60 |
| rs72689147 | 4 | 1.57E+08 | G | T | 0.818646 | 0.31275 | 0.214309 | 0.14 | Age 56-60 |
| rs10069690 | 5 | 1279790 | C | T | 0.74078 | -0.46117 | 0.187988 | 0.014 | Age 56-60 |
| rs7733331 | 5 | 32828846 | T | C | 0.402121 | -0.94394 | 0.168435 | 2.10E-08 | Age 56-60 |
| rs2112453 | 5 | 1.12E+08 | G | A | 0.265737 | 0.270664 | 0.186943 | 0.15 | Age 56-60 |
| rs4487481 | 5 | 1.14E+08 | T | C | 0.483604 | 0.229859 | 0.164865 | 0.16 | Age 56-60 |
| rs6890251 | 5 | 1.22E+08 | C | T | 0.849878 | -0.34627 | 0.231432 | 0.13 | Age 56-60 |
| rs7703751 | 5 | 1.23E+08 | A | T | 0.740623 | 0.536149 | 0.187507 | 0.0042 | Age 56-60 |
| rs6892983 | 5 | 1.28E+08 | C | A | 0.591613 | -0.41573 | 0.167889 | 0.013 | Age 56-60 |
| rs7737361 | 5 | 1.48E+08 | G | A | 0.80745 | 0.550612 | 0.213219 | 0.0098 | Age 56-60 |
| rs1650581 | 5 | 1.58E+08 | C | G | 0.736859 | -0.52492 | 0.18861 | 0.0054 | Age 56-60 |
| rs12187017 | 5 | 1.58E+08 | G | A | 0.638445 | 0.399381 | 0.171818 | 0.02 | Age 56-60 |
| rs198851 | 6 | 26104632 | T | G | 0.153366 | 0.159784 | 0.229961 | 0.49 | Age 56-60 |
| rs1265157 | 6 | 31142265 | C | G | 0.651282 | 0.108893 | 0.172532 | 0.53 | Age 56-60 |
| rs2856448 | 6 | 32014575 | A | G | 0.532597 | -0.23168 | 0.166734 | 0.16 | Age 56-60 |
| rs1214761 | 6 | 43354431 | A | G | 0.322909 | 0.295418 | 0.177913 | 0.097 | Age 56-60 |
| rs211159 | 6 | 97023026 | G | A | 0.657023 | -0.47125 | 0.174891 | 0.007 | Age 56-60 |
| rs4897160 | 6 | 1.26E+08 | G | A | 0.522526 | -0.21714 | 0.164357 | 0.19 | Age 56-60 |
| rs9375459 | 6 | 1.27E+08 | C | T | 0.562512 | -0.66419 | 0.166713 | 6.80E-05 | Age 56-60 |
| rs62434129 | 6 | 1.51E+08 | A | T | 0.927864 | 1.06168 | 0.320779 | 0.00093 | Age 56-60 |
| rs2969036 | 7 | 2534901 | T | G | 0.309895 | 0.086199 | 0.178854 | 0.63 | Age 56-60 |
| rs3735533 | 7 | 27245893 | T | C | 0.076198 | -1.17881 | 0.30976 | 0.00014 | Age 56-60 |
| rs6961048 | 7 | 27328187 | C | G | 0.900267 | -0.33766 | 0.274828 | 0.22 | Age 56-60 |
| rs2392929 | 7 | 1.06E+08 | T | G | 0.802515 | -0.91211 | 0.207168 | 1.10E-05 | Age 56-60 |
| rs3807306 | 7 | 1.29E+08 | G | T | 0.506273 | 0.074607 | 0.164461 | 0.65 | Age 56-60 |
| rs3918226 | 7 | 1.51E+08 | C | T | 0.918311 | -1.0722 | 0.306684 | 0.00047 | Age 56-60 |
| rs1991651 | 8 | 10706411 | C | G | 0.381336 | 0.433005 | 0.171118 | 0.011 | Age 56-60 |
| rs1352026 | 8 | 25895899 | C | T | 0.74784 | 0.343854 | 0.189664 | 0.07 | Age 56-60 |
| rs830444 | 8 | 76954113 | G | T | 0.39952 | -0.50431 | 0.168596 | 0.0028 | Age 56-60 |
| rs35783704 | 8 | 1.06E+08 | G | A | 0.899722 | 0.599062 | 0.277272 | 0.031 | Age 56-60 |
| rs62521878 | 8 | 1.42E+08 | C | T | 0.704881 | -0.33546 | 0.181363 | 0.064 | Age 56-60 |
| rs72762681 | 9 | 1.13E+08 | A | T | 0.84703 | -0.07588 | 0.229297 | 0.74 | Age 56-60 |
| rs1623474 | 10 | 18471794 | C | T | 0.67026 | -0.51701 | 0.165694 | 0.0018 | Age 61-65 |
| rs12258967 | 10 | 18727959 | C | G | 0.699736 | 0.73349 | 0.170812 | 1.80E-05 | Age 61-65 |
| rs57946343 | 10 | 63499951 | T | C | 0.847495 | 1.16121 | 0.217622 | 9.50E-08 | Age 61-65 |
| rs10128354 | 10 | 75470045 | T | C | 0.416419 | 0.362502 | 0.158512 | 0.022 | Age 61-65 |
| rs10882398 | 10 | 95892788 | T | A | 0.56359 | -0.6053 | 0.158274 | 0.00013 | Age 61-65 |
| rs1006545 | 10 | 1.03E+08 | G | T | 0.112763 | -1.01216 | 0.24684 | 4.10E-05 | Age 61-65 |
| rs11191551 | 10 | 1.05E+08 | A | G | 0.924442 | 1.55808 | 0.294935 | 1.30E-07 | Age 61-65 |
| rs1801253 | 10 | 1.16E+08 | G | C | 0.262397 | -0.79329 | 0.179523 | 9.90E-06 | Age 61-65 |
| rs11592107 | 10 | 1.23E+08 | G | A | 0.687226 | -0.33722 | 0.168562 | 0.045 | Age 61-65 |
| rs592373 | 11 | 1890990 | G | A | 0.369606 | -0.81919 | 0.162076 | 4.30E-07 | Age 61-65 |
| rs2218305 | 11 | 10247896 | G | C | 0.506494 | -0.42708 | 0.156752 | 0.0064 | Age 61-65 |
| rs1966698 | 11 | 16306267 | C | T | 0.203841 | 0.787238 | 0.194261 | 5.10E-05 | Age 61-65 |
| rs416258 | 11 | 16911636 | G | C | 0.697336 | -0.24012 | 0.170781 | 0.16 | Age 61-65 |
| rs7107356 | 11 | 47676170 | A | G | 0.496348 | -0.45406 | 0.156523 | 0.0037 | Age 61-65 |
| rs10750766 | 11 | 65473798 | C | A | 0.291637 | -0.56288 | 0.172585 | 0.0011 | Age 61-65 |
| rs139437879 | 11 | 67904333 | G | A | 0.983842 | 0.361335 | 0.63082 | 0.57 | Age 61-65 |
| rs58941166 | 11 | 72083441 | G | A | 0.933188 | -0.14143 | 0.313826 | 0.65 | Age 61-65 |
| rs604723 | 11 | 1.01E+08 | T | C | 0.276328 | -0.62364 | 0.17601 | 4.00E-04 | Age 61-65 |
| rs2072927 | 1 | 1684800 | G | A | 0.501055 | 0.596568 | 0.156355 | 0.00014 | Age 61-65 |
| rs488834 | 1 | 10767902 | C | T | 0.232402 | 0.431994 | 0.184753 | 0.019 | Age 61-65 |
| rs6669371 | 1 | 11882142 | T | G | 0.834861 | 0.898367 | 0.211405 | 2.10E-05 | Age 61-65 |
| rs75461554 | 1 | 15810172 | C | T | 0.804953 | 0.318594 | 0.196457 | 0.1 | Age 61-65 |
| rs4908348 | 1 | 28706949 | T | G | 0.694987 | 0.471049 | 0.169344 | 0.0054 | Age 61-65 |
| rs1408945 | 1 | 42364877 | G | T | 0.573157 | 0.208237 | 0.158286 | 0.19 | Age 61-65 |
| rs12132894 | 1 | 89138474 | A | T | 0.579054 | 0.300424 | 0.158173 | 0.058 | Age 61-65 |
| rs10776752 | 1 | 1.13E+08 | G | T | 0.927242 | -1.02783 | 0.301288 | 0.00065 | Age 61-65 |
| rs10914117 | 1 | 1.81E+08 | T | C | 0.593218 | 0.348688 | 0.159088 | 0.028 | Age 61-65 |
| rs68085857 | 1 | 2.18E+08 | C | T | 0.771603 | -0.37903 | 0.185802 | 0.041 | Age 61-65 |
| rs1745417 | 1 | 2.28E+08 | C | T | 0.485707 | -0.37721 | 0.156457 | 0.016 | Age 61-65 |
| rs699 | 1 | 2.31E+08 | A | G | 0.601984 | -0.25397 | 0.159253 | 0.11 | Age 61-65 |
| rs3213751 | 12 | 432666 | G | C | 0.740361 | 0.054193 | 0.178989 | 0.76 | Age 61-65 |
| rs11055034 | 12 | 12890626 | C | A | 0.716443 | 0.426153 | 0.17447 | 0.015 | Age 61-65 |
| rs1010064 | 12 | 20000315 | A | C | 0.8137 | 0.686084 | 0.200904 | 0.00064 | Age 61-65 |
| rs73075659 | 12 | 20373541 | A | G | 0.652348 | 0.452976 | 0.164743 | 0.006 | Age 61-65 |
| rs150857355 | 12 | 49209340 | G | C | 0.977805 | -0.73924 | 0.53813 | 0.17 | Age 61-65 |
| rs7967954 | 12 | 50673484 | G | A | 0.42337 | 0.244174 | 0.158564 | 0.12 | Age 61-65 |
| rs736825 | 12 | 54417576 | C | G | 0.636981 | 0.373297 | 0.163344 | 0.022 | Age 61-65 |
| rs4143175 | 12 | 67782397 | T | C | 0.242844 | 0.289132 | 0.182526 | 0.11 | Age 61-65 |
| rs17249754 | 12 | 90060586 | G | A | 0.826764 | 0.832804 | 0.207379 | 5.90E-05 | Age 61-65 |
| rs7310615 | 12 | 1.12E+08 | C | G | 0.484454 | 0.390801 | 0.157258 | 0.013 | Age 61-65 |
| rs35443 | 12 | 1.16E+08 | G | C | 0.614522 | 0.548931 | 0.160818 | 0.00064 | Age 61-65 |
| rs6490021 | 12 | 1.16E+08 | A | C | 0.381243 | -0.58321 | 0.161722 | 0.00031 | Age 61-65 |
| rs9508495 | 13 | 30146201 | C | T | 0.236653 | 0.080336 | 0.183238 | 0.66 | Age 61-65 |
| rs4415898 | 13 | 41895177 | T | A | 0.977481 | -0.64743 | 0.593699 | 0.28 | Age 61-65 |
| rs4415898 | 13 | 41895177 | T | G | 0.166261 | -0.10522 | 0.21108 | 0.62 | Age 61-65 |
| rs8904 | 14 | 35871217 | G | A | 0.636491 | -0.54714 | 0.16229 | 0.00075 | Age 61-65 |
| rs28365937 | 14 | 53418233 | C | A | 0.695131 | -0.23606 | 0.170292 | 0.17 | Age 61-65 |
| rs12591513 | 15 | 75102714 | G | A | 0.329235 | 0.274295 | 0.166757 | 0.1 | Age 61-65 |
| rs2627316 | 15 | 81042816 | A | G | 0.53101 | -0.53216 | 0.157039 | 7.00E-04 | Age 61-65 |
| rs4932373 | 15 | 91429287 | A | C | 0.676141 | -0.74102 | 0.166478 | 8.50E-06 | Age 61-65 |
| rs10500326 | 16 | 4918326 | G | T | 0.764453 | 0.450785 | 0.184809 | 0.015 | Age 61-65 |
| rs4293393 | 16 | 20364588 | A | G | 0.81767 | 0.198894 | 0.201911 | 0.32 | Age 61-65 |
| rs4888408 | 16 | 75432824 | G | A | 0.402504 | -0.22247 | 0.158728 | 0.16 | Age 61-65 |
| rs9806885 | 16 | 81556909 | A | G | 0.888047 | 0.464456 | 0.247966 | 0.061 | Age 61-65 |
| rs908951 | 16 | 89697625 | C | T | 0.570635 | 0.080381 | 0.159438 | 0.61 | Age 61-65 |
| rs222857 | 17 | 7164563 | C | T | 0.425687 | -0.49824 | 0.158639 | 0.0017 | Age 61-65 |
| rs7213273 | 17 | 43155914 | G | A | 0.346453 | 0.434914 | 0.165245 | 0.0085 | Age 61-65 |
| rs17608766 | 17 | 45013271 | T | C | 0.854001 | -0.80181 | 0.220691 | 0.00028 | Age 61-65 |
| rs7406910 | 17 | 46688256 | T | C | 0.088842 | -0.3467 | 0.273645 | 0.21 | Age 61-65 |
| rs2671661 | 17 | 47528562 | T | C | 0.270627 | -0.25131 | 0.177739 | 0.16 | Age 61-65 |
| rs1000423 | 17 | 59475642 | C | T | 0.265826 | -0.71543 | 0.17775 | 5.70E-05 | Age 61-65 |
| rs77946446 | 17 | 75317932 | C | T | 0.733996 | 0.446745 | 0.178075 | 0.012 | Age 61-65 |
| rs12955644 | 18 | 771047 | C | G | 0.817666 | 0.001083 | 0.204752 | 1 | Age 61-65 |
| rs56407827 | 18 | 42179819 | C | T | 0.734681 | -0.15918 | 0.177594 | 0.37 | Age 61-65 |
| rs76978865 | 19 | 2175799 | G | A | 0.959179 | -1.05392 | 0.395197 | 0.0077 | Age 61-65 |
| rs10427021 | 19 | 7259346 | T | G | 0.889565 | 0.796925 | 0.274684 | 0.0037 | Age 61-65 |
| rs79709447 | 19 | 12187621 | T | C | 0.986851 | 0.391213 | 0.694461 | 0.57 | Age 61-65 |
| rs1327235 | 20 | 10969030 | A | G | 0.522329 | -0.37209 | 0.157015 | 0.018 | Age 61-65 |
| rs17812022 | 20 | 19007099 | C | T | 0.904365 | 0.535459 | 0.266922 | 0.045 | Age 61-65 |
| rs6102506 | 20 | 40254735 | C | T | 0.648978 | 0.408503 | 0.165945 | 0.014 | Age 61-65 |
| rs6026744 | 20 | 57742388 | A | T | 0.881725 | -1.02016 | 0.242538 | 2.60E-05 | Age 61-65 |
| rs1275988 | 2 | 26914364 | C | T | 0.383928 | 0.635444 | 0.160101 | 7.20E-05 | Age 61-65 |
| rs35441226 | 2 | 43191137 | C | T | 0.818221 | 0.173098 | 0.203233 | 0.39 | Age 61-65 |
| rs56308618 | 21 | 44757792 | G | A | 0.70631 | -0.16638 | 0.172566 | 0.33 | Age 61-65 |
| rs58117425 | 2 | 1.46E+08 | G | A | 0.762045 | -0.34615 | 0.183962 | 0.06 | Age 61-65 |
| rs73029563 | 2 | 1.65E+08 | C | G | 0.456433 | -0.54704 | 0.156582 | 0.00048 | Age 61-65 |
| rs10205122 | 2 | 1.81E+08 | A | G | 0.71716 | 0.150112 | 0.175402 | 0.39 | Age 61-65 |
| rs7592578 | 2 | 1.91E+08 | T | G | 0.184056 | 0.058564 | 0.200502 | 0.77 | Age 61-65 |
| rs3845811 | 2 | 2.09E+08 | C | G | 0.565438 | -0.28707 | 0.157583 | 0.068 | Age 61-65 |
| rs2161967 | 2 | 2.19E+08 | T | G | 0.425225 | 0.466112 | 0.158586 | 0.0033 | Age 61-65 |
| rs2594992 | 3 | 11360997 | A | C | 0.596402 | -0.29813 | 0.159298 | 0.061 | Age 61-65 |
| rs34991912 | 3 | 14926351 | T | C | 0.414638 | 0.124745 | 0.159229 | 0.43 | Age 61-65 |
| rs2643826 | 3 | 27562988 | C | T | 0.550342 | -0.72882 | 0.157503 | 3.70E-06 | Age 61-65 |
| rs6771917 | 3 | 48108442 | T | C | 0.243829 | -0.10673 | 0.183458 | 0.56 | Age 61-65 |
| rs3772219 | 3 | 56771251 | A | C | 0.674217 | 0.312183 | 0.166028 | 0.06 | Age 61-65 |
| rs73872710 | 3 | 1.41E+08 | A | T | 0.924052 | -0.62017 | 0.295697 | 0.036 | Age 61-65 |
| rs1597467 | 3 | 1.5E+08 | C | A | 0.908991 | -0.36304 | 0.272882 | 0.18 | Age 61-65 |
| rs11715377 | 3 | 1.69E+08 | A | T | 0.905184 | 0.364793 | 0.265411 | 0.17 | Age 61-65 |
| rs1528293 | 3 | 1.69E+08 | A | T | 0.489379 | 0.257385 | 0.156393 | 0.1 | Age 61-65 |
| rs16852391 | 4 | 40604309 | C | G | 0.89789 | -0.34752 | 0.257855 | 0.18 | Age 61-65 |
| rs12509595 | 4 | 81182554 | T | C | 0.706798 | -0.67181 | 0.171985 | 9.40E-05 | Age 61-65 |
| rs17010957 | 4 | 86719165 | T | C | 0.851549 | -0.3411 | 0.221938 | 0.12 | Age 61-65 |
| rs13135092 | 4 | 1.03E+08 | A | G | 0.914793 | 0.370317 | 0.280686 | 0.19 | Age 61-65 |
| rs6820343 | 4 | 1.56E+08 | C | T | 0.468075 | 0.358062 | 0.156264 | 0.022 | Age 61-65 |
| rs72689147 | 4 | 1.57E+08 | G | T | 0.816062 | 0.196197 | 0.201377 | 0.33 | Age 61-65 |
| rs10069690 | 5 | 1279790 | C | T | 0.742386 | -0.05736 | 0.178334 | 0.75 | Age 61-65 |
| rs7733331 | 5 | 32828846 | T | C | 0.399865 | -1.01697 | 0.160007 | 2.10E-10 | Age 61-65 |
| rs2112453 | 5 | 1.12E+08 | G | A | 0.263871 | 0.280533 | 0.177726 | 0.11 | Age 61-65 |
| rs4487481 | 5 | 1.14E+08 | T | C | 0.48219 | 0.280474 | 0.156792 | 0.074 | Age 61-65 |
| rs6890251 | 5 | 1.22E+08 | C | T | 0.847152 | -0.38736 | 0.217708 | 0.075 | Age 61-65 |
| rs7703751 | 5 | 1.23E+08 | A | T | 0.740664 | 0.611447 | 0.179381 | 0.00065 | Age 61-65 |
| rs6892983 | 5 | 1.28E+08 | C | A | 0.589441 | -0.14478 | 0.158985 | 0.36 | Age 61-65 |
| rs7737361 | 5 | 1.48E+08 | G | A | 0.807232 | 0.472259 | 0.201363 | 0.019 | Age 61-65 |
| rs1650581 | 5 | 1.58E+08 | C | G | 0.738799 | -0.23256 | 0.178994 | 0.19 | Age 61-65 |
| rs12187017 | 5 | 1.58E+08 | G | A | 0.639617 | 0.470728 | 0.163363 | 0.004 | Age 61-65 |
| rs198851 | 6 | 26104632 | T | G | 0.152106 | 0.680541 | 0.217375 | 0.0017 | Age 61-65 |
| rs1265157 | 6 | 31142265 | C | G | 0.653282 | 0.279385 | 0.164421 | 0.089 | Age 61-65 |
| rs2856448 | 6 | 32014575 | A | G | 0.530215 | -0.44341 | 0.156631 | 0.0046 | Age 61-65 |
| rs1214761 | 6 | 43354431 | A | G | 0.320044 | 0.568361 | 0.167446 | 0.00069 | Age 61-65 |
| rs211159 | 6 | 97023026 | G | A | 0.659752 | 0.031917 | 0.164775 | 0.85 | Age 61-65 |
| rs4897160 | 6 | 1.26E+08 | G | A | 0.522345 | -0.03408 | 0.15634 | 0.83 | Age 61-65 |
| rs9375459 | 6 | 1.27E+08 | C | T | 0.562924 | -0.79362 | 0.157636 | 4.80E-07 | Age 61-65 |
| rs62434129 | 6 | 1.51E+08 | A | T | 0.929368 | 0.680463 | 0.305821 | 0.026 | Age 61-65 |
| rs2969036 | 7 | 2534901 | T | G | 0.312913 | 0.428554 | 0.16941 | 0.011 | Age 61-65 |
| rs3735533 | 7 | 27245893 | T | C | 0.071773 | -1.72109 | 0.30241 | 1.30E-08 | Age 61-65 |
| rs6961048 | 7 | 27328187 | C | G | 0.900114 | -0.62434 | 0.25979 | 0.016 | Age 61-65 |
| rs2392929 | 7 | 1.06E+08 | T | G | 0.804297 | -1.24003 | 0.196801 | 3.00E-10 | Age 61-65 |
| rs3807306 | 7 | 1.29E+08 | G | T | 0.506844 | 0.188383 | 0.15647 | 0.23 | Age 61-65 |
| rs3918226 | 7 | 1.51E+08 | C | T | 0.919429 | -0.66984 | 0.291622 | 0.022 | Age 61-65 |
| rs1991651 | 8 | 10706411 | C | G | 0.377755 | 0.291468 | 0.161212 | 0.071 | Age 61-65 |
| rs1352026 | 8 | 25895899 | C | T | 0.746306 | 0.356754 | 0.179989 | 0.047 | Age 61-65 |
| rs830444 | 8 | 76954113 | G | T | 0.396584 | -0.11483 | 0.159764 | 0.47 | Age 61-65 |
| rs35783704 | 8 | 1.06E+08 | G | A | 0.899221 | 1.09538 | 0.26245 | 3.00E-05 | Age 61-65 |
| rs62521878 | 8 | 1.42E+08 | C | T | 0.706412 | -0.21704 | 0.171673 | 0.21 | Age 61-65 |
| rs72762681 | 9 | 1.13E+08 | A | T | 0.85091 | -0.28056 | 0.220616 | 0.2 | Age 61-65 |

**Supplemental Table 4.** Genetic instruments used for LDL-C for analyses of incident CHD

| SNP | CHR | BP | ALLELE1 | ALLELE0 | A1FREQ | BETA | SE | P | phenotype |
| --- | --- | --- | --- | --- | --- | --- | --- | --- | --- |
| rs1129555 | 10 | 1.14E+08 | A | G | 0.275965 | 0.024064 | 0.005212 | 3.90E-06 | Age ≤55 |
| rs10128711 | 11 | 18632984 | T | C | 0.260873 | -0.02324 | 0.005292 | 1.10E-05 | Age ≤55 |
| rs149803 | 11 | 61539020 | C | G | 0.74408 | -0.0168 | 0.00542 | 0.0019 | Age ≤55 |
| rs499790 | 11 | 1.17E+08 | C | T | 0.897048 | -0.03587 | 0.00778 | 4.00E-06 | Age ≤55 |
| rs61905078 | 11 | 1.17E+08 | A | C | 0.926432 | -0.07181 | 0.008901 | 7.20E-16 | Age ≤55 |
| rs11220437 | 11 | 1.26E+08 | T | C | 0.885994 | -0.0285 | 0.007326 | 1.00E-04 | Age ≤55 |
| rs3093584 | 1 | 25668714 | T | C | 0.557348 | 0.021704 | 0.004633 | 2.80E-06 | Age ≤55 |
| rs12753981 | 1 | 27001893 | G | A | 0.915917 | -0.02442 | 0.008314 | 0.0033 | Age ≤55 |
| rs2495504 | 1 | 55485796 | C | T | 0.279206 | 0.027868 | 0.005148 | 6.20E-08 | Age ≤55 |
| rs2479408 | 1 | 55504188 | C | G | 0.788328 | 0.028854 | 0.005751 | 5.20E-07 | Age ≤55 |
| rs2495477 | 1 | 55518467 | A | G | 0.607571 | 0.041614 | 0.004822 | 6.20E-18 | Age ≤55 |
| rs626787 | 1 | 62901243 | C | G | 0.647505 | 0.050135 | 0.004849 | 4.60E-25 | Age ≤55 |
| rs1386585 | 1 | 92715207 | C | T | 0.193212 | -0.00969 | 0.005843 | 0.097 | Age ≤55 |
| rs1337247 | 1 | 1.1E+08 | C | A | 0.854437 | 0.050552 | 0.006532 | 1.00E-14 | Age ≤55 |
| rs4268379 | 1 | 1.1E+08 | C | T | 0.49658 | -0.03981 | 0.00461 | 5.80E-18 | Age ≤55 |
| rs267733 | 1 | 1.51E+08 | A | G | 0.839415 | 0.024299 | 0.00629 | 0.00011 | Age ≤55 |
| rs2642438 | 1 | 2.21E+08 | A | G | 0.296443 | -0.02962 | 0.005041 | 4.20E-09 | Age ≤55 |
| rs487738 | 1 | 2.35E+08 | A | G | 0.329031 | -0.03826 | 0.005182 | 1.50E-13 | Age ≤55 |
| rs3184504 | 12 | 1.12E+08 | T | C | 0.480964 | -0.01647 | 0.004653 | 4.00E-04 | Age ≤55 |
| rs2649999 | 12 | 1.21E+08 | T | C | 0.332107 | 0.027985 | 0.005016 | 2.40E-08 | Age ≤55 |
| rs206070 | 13 | 32896846 | G | A | 0.82013 | 0.002917 | 0.006076 | 0.63 | Age ≤55 |
| rs6573778 | 14 | 24872209 | T | C | 0.481042 | 0.018698 | 0.004672 | 6.30E-05 | Age ≤55 |
| rs9989419 | 16 | 56985139 | A | G | 0.394698 | 0.021309 | 0.004742 | 7.00E-06 | Age ≤55 |
| rs9932707 | 16 | 71862133 | A | C | 0.541564 | -0.02812 | 0.004685 | 1.90E-09 | Age ≤55 |
| rs4788597 | 16 | 72043039 | C | T | 0.638186 | 0.03114 | 0.004859 | 1.50E-10 | Age ≤55 |
| rs11650232 | 17 | 7088923 | A | G | 0.556048 | -0.00824 | 0.004659 | 0.077 | Age ≤55 |
| rs7225700 | 17 | 45391804 | T | C | 0.352439 | -0.02181 | 0.00485 | 6.90E-06 | Age ≤55 |
| rs1801689 | 17 | 64210580 | A | C | 0.969808 | -0.09226 | 0.013517 | 8.80E-12 | Age ≤55 |
| rs2886232 | 17 | 67150176 | T | C | 0.12207 | 0.037454 | 0.007109 | 1.40E-07 | Age ≤55 |
| rs17677316 | 19 | 10680241 | G | A | 0.752296 | -0.01236 | 0.005332 | 0.02 | Age ≤55 |
| rs1529711 | 19 | 11023434 | C | T | 0.838572 | -0.02715 | 0.006244 | 1.40E-05 | Age ≤55 |
| rs17657025 | 19 | 44675801 | T | C | 0.92085 | 0.044481 | 0.008523 | 1.80E-07 | Age ≤55 |
| rs1004165 | 19 | 45232205 | G | A | 0.561739 | -0.03392 | 0.004663 | 3.50E-13 | Age ≤55 |
| rs11668536 | 19 | 45328476 | C | T | 0.752992 | 0.020175 | 0.005351 | 0.00016 | Age ≤55 |
| rs6857 | 19 | 45392254 | C | T | 0.829655 | -0.18785 | 0.006148 | 4.90E-205 | Age ≤55 |
| rs364585 | 20 | 12962718 | A | G | 0.392457 | -0.00871 | 0.004768 | 0.068 | Age ≤55 |
| rs2328223 | 20 | 17845921 | A | C | 0.813993 | -0.01812 | 0.005973 | 0.0024 | Age ≤55 |
| rs7264396 | 20 | 34154741 | C | T | 0.790192 | 0.021747 | 0.005722 | 0.00014 | Age ≤55 |
| rs6016373 | 20 | 39154095 | A | G | 0.592107 | 0.032986 | 0.004816 | 7.40E-12 | Age ≤55 |
| rs12624863 | 20 | 39782899 | A | G | 0.792027 | -0.0295 | 0.00573 | 2.60E-07 | Age ≤55 |
| rs1800961 | 20 | 43042364 | C | T | 0.969443 | 0.071757 | 0.013499 | 1.10E-07 | Age ≤55 |
| rs13027716 | 2 | 20887226 | T | G | 0.969769 | 0.073542 | 0.013786 | 9.60E-08 | Age ≤55 |
| rs11124924 | 2 | 21206275 | G | C | 0.94 | 0.072196 | 0.009715 | 1.10E-13 | Age ≤55 |
| rs12720842 | 2 | 21257927 | T | C | 0.962884 | -0.07631 | 0.01221 | 4.10E-10 | Age ≤55 |
| rs6756743 | 2 | 21301892 | C | T | 0.952928 | -0.03201 | 0.010951 | 0.0035 | Age ≤55 |
| rs10495907 | 2 | 43998726 | G | A | 0.868103 | -0.01568 | 0.006832 | 0.022 | Age ≤55 |
| rs3923912 | 2 | 44057570 | A | C | 0.937914 | 0.119502 | 0.00962 | 2.00E-35 | Age ≤55 |
| rs4148217 | 2 | 44099433 | C | A | 0.816565 | 0.035269 | 0.005971 | 3.50E-09 | Age ≤55 |
| rs11125936 | 2 | 62871225 | T | C | 0.907479 | 0.036535 | 0.007939 | 4.20E-06 | Age ≤55 |
| rs17508045 | 2 | 1.19E+08 | T | C | 0.91238 | 0.047571 | 0.008186 | 6.20E-09 | Age ≤55 |
| rs16831243 | 2 | 1.36E+08 | C | T | 0.898 | -0.0107 | 0.007699 | 0.16 | Age ≤55 |
| rs10195252 | 2 | 1.66E+08 | T | C | 0.59432 | 0.020947 | 0.004698 | 8.30E-06 | Age ≤55 |
| rs1250229 | 2 | 2.16E+08 | T | C | 0.260144 | -0.01596 | 0.00529 | 0.0025 | Age ≤55 |
| rs11563251 | 2 | 2.35E+08 | C | T | 0.889225 | -0.0252 | 0.007335 | 0.00059 | Age ≤55 |
| rs4823057 | 22 | 30241061 | A | G | 0.976935 | -0.05623 | 0.015621 | 0.00032 | Age ≤55 |
| rs4253772 | 22 | 46627603 | C | T | 0.88992 | -0.0177 | 0.007437 | 0.017 | Age ≤55 |
| rs7616006 | 3 | 12267648 | A | G | 0.578592 | 0.026956 | 0.004701 | 9.80E-09 | Age ≤55 |
| rs7640978 | 3 | 32533010 | C | T | 0.90992 | 0.032805 | 0.008098 | 5.10E-05 | Age ≤55 |
| rs17404153 | 3 | 1.32E+08 | G | T | 0.875465 | 0.025585 | 0.007029 | 0.00027 | Age ≤55 |
| rs6818397 | 4 | 3434885 | T | G | 0.385261 | 0.021309 | 0.004821 | 9.90E-06 | Age ≤55 |
| rs4072296 | 5 | 74242002 | T | C | 0.457586 | -0.02505 | 0.004806 | 1.90E-07 | Age ≤55 |
| rs16872670 | 5 | 74929312 | G | A | 0.952806 | -0.02285 | 0.01099 | 0.038 | Age ≤55 |
| rs4530754 | 5 | 1.23E+08 | G | A | 0.455421 | -0.01572 | 0.004651 | 0.00072 | Age ≤55 |
| rs4704810 | 5 | 1.56E+08 | G | A | 0.627687 | -0.01818 | 0.004864 | 0.00019 | Age ≤55 |
| rs2294266 | 6 | 16107867 | A | C | 0.51108 | 0.032636 | 0.004669 | 2.70E-12 | Age ≤55 |
| rs1408272 | 6 | 25842951 | T | G | 0.922153 | 0.055923 | 0.008648 | 1.00E-10 | Age ≤55 |
| rs1049281 | 6 | 31236567 | T | C | 0.360189 | -0.01685 | 0.004838 | 5.00E-04 | Age ≤55 |
| rs3798236 | 6 | 1.16E+08 | T | C | 0.630485 | 0.015946 | 0.004842 | 0.00099 | Age ≤55 |
| rs6917747 | 6 | 1.6E+08 | G | A | 0.863325 | -0.03779 | 0.006786 | 2.60E-08 | Age ≤55 |
| rs9347386 | 6 | 1.61E+08 | A | G | 0.62334 | 0.028288 | 0.004815 | 4.20E-09 | Age ≤55 |
| rs6415084 | 6 | 1.61E+08 | T | C | 0.479357 | 0.025637 | 0.004662 | 3.80E-08 | Age ≤55 |
| rs2390536 | 7 | 21485397 | G | A | 0.63617 | -0.02342 | 0.004835 | 1.30E-06 | Age ≤55 |
| rs4722551 | 7 | 25991826 | T | C | 0.841691 | -0.03745 | 0.006355 | 3.80E-09 | Age ≤55 |
| rs7798185 | 7 | 44570717 | C | A | 0.829484 | -0.04235 | 0.006178 | 7.10E-12 | Age ≤55 |
| rs713286 | 8 | 9171735 | T | C | 0.920193 | 0.015817 | 0.00868 | 0.068 | Age ≤55 |
| rs10102164 | 8 | 55421614 | G | A | 0.788015 | -0.03151 | 0.005673 | 2.80E-08 | Age ≤55 |
| rs2737229 | 8 | 1.17E+08 | A | C | 0.701915 | 0.029474 | 0.005069 | 6.10E-09 | Age ≤55 |
| rs17320616 | 8 | 1.26E+08 | A | G | 0.796535 | -0.03656 | 0.005787 | 2.60E-10 | Age ≤55 |
| rs7014582 | 8 | 1.45E+08 | A | G | 0.567125 | -0.01908 | 0.004696 | 4.80E-05 | Age ≤55 |
| rs3780181 | 9 | 2640759 | A | G | 0.930901 | 0.041956 | 0.009213 | 5.30E-06 | Age ≤55 |
| rs12686004 | 9 | 1.08E+08 | G | A | 0.884733 | 0.034931 | 0.007297 | 1.70E-06 | Age ≤55 |
| rs7849542 | 9 | 1.36E+08 | G | A | 0.700344 | -0.01802 | 0.005079 | 0.00039 | Age ≤55 |
| rs7030248 | 9 | 1.36E+08 | G | A | 0.656126 | 0.016603 | 0.004919 | 0.00074 | Age ≤55 |
| rs1129555 | 10 | 1.14E+08 | A | G | 0.27562 | -0.0035 | 0.007965 | 0.66 | Age 56-60 |
| rs10128711 | 11 | 18632984 | T | C | 0.259375 | -0.02481 | 0.008116 | 0.0022 | Age 56-60 |
| rs149803 | 11 | 61539020 | C | G | 0.743375 | -0.03239 | 0.008339 | 1.00E-04 | Age 56-60 |
| rs499790 | 11 | 1.17E+08 | C | T | 0.894883 | -0.04026 | 0.011751 | 0.00061 | Age 56-60 |
| rs61905078 | 11 | 1.17E+08 | A | C | 0.927387 | -0.06824 | 0.013694 | 6.20E-07 | Age 56-60 |
| rs11220437 | 11 | 1.26E+08 | T | C | 0.882322 | -0.03606 | 0.010991 | 0.001 | Age 56-60 |
| rs3093584 | 1 | 25668714 | T | C | 0.559415 | 0.026753 | 0.007147 | 0.00018 | Age 56-60 |
| rs12753981 | 1 | 27001893 | G | A | 0.915958 | -0.00421 | 0.012787 | 0.74 | Age 56-60 |
| rs2495504 | 1 | 55485796 | C | T | 0.280429 | 0.044087 | 0.007875 | 2.20E-08 | Age 56-60 |
| rs2479408 | 1 | 55504188 | C | G | 0.789527 | 0.033921 | 0.008871 | 0.00013 | Age 56-60 |
| rs2495477 | 1 | 55518467 | A | G | 0.608763 | 0.033455 | 0.007375 | 5.70E-06 | Age 56-60 |
| rs626787 | 1 | 62901243 | C | G | 0.649038 | 0.058237 | 0.007415 | 4.00E-15 | Age 56-60 |
| rs1386585 | 1 | 92715207 | C | T | 0.195635 | -0.02282 | 0.008931 | 0.011 | Age 56-60 |
| rs1337247 | 1 | 1.1E+08 | C | A | 0.85498 | 0.056199 | 0.010071 | 2.40E-08 | Age 56-60 |
| rs4268379 | 1 | 1.1E+08 | C | T | 0.497852 | -0.04666 | 0.007123 | 5.70E-11 | Age 56-60 |
| rs267733 | 1 | 1.51E+08 | A | G | 0.838062 | 0.0098 | 0.009606 | 0.31 | Age 56-60 |
| rs2642438 | 1 | 2.21E+08 | A | G | 0.296932 | -0.04155 | 0.00774 | 8.00E-08 | Age 56-60 |
| rs487738 | 1 | 2.35E+08 | A | G | 0.327503 | -0.0254 | 0.007947 | 0.0014 | Age 56-60 |
| rs3184504 | 12 | 1.12E+08 | T | C | 0.484365 | -0.0198 | 0.007084 | 0.0052 | Age 56-60 |
| rs2649999 | 12 | 1.21E+08 | T | C | 0.328788 | 0.034509 | 0.007701 | 7.40E-06 | Age 56-60 |
| rs206070 | 13 | 32896846 | G | A | 0.821675 | -0.01456 | 0.009298 | 0.12 | Age 56-60 |
| rs6573778 | 14 | 24872209 | T | C | 0.483162 | 0.023242 | 0.00717 | 0.0012 | Age 56-60 |
| rs9989419 | 16 | 56985139 | A | G | 0.395817 | 0.023275 | 0.007223 | 0.0013 | Age 56-60 |
| rs9932707 | 16 | 71862133 | A | C | 0.543565 | -0.04146 | 0.00712 | 5.80E-09 | Age 56-60 |
| rs4788597 | 16 | 72043039 | C | T | 0.634787 | 0.030287 | 0.007417 | 4.40E-05 | Age 56-60 |
| rs11650232 | 17 | 7088923 | A | G | 0.553044 | -0.01085 | 0.007172 | 0.13 | Age 56-60 |
| rs7225700 | 17 | 45391804 | T | C | 0.355028 | -0.03063 | 0.007401 | 3.50E-05 | Age 56-60 |
| rs1801689 | 17 | 64210580 | A | C | 0.969665 | -0.06759 | 0.02076 | 0.0011 | Age 56-60 |
| rs2886232 | 17 | 67150176 | T | C | 0.124656 | 0.043224 | 0.010737 | 5.70E-05 | Age 56-60 |
| rs17677316 | 19 | 10680241 | G | A | 0.75396 | -0.03133 | 0.008163 | 0.00012 | Age 56-60 |
| rs1529711 | 19 | 11023434 | C | T | 0.84127 | -0.0147 | 0.009683 | 0.13 | Age 56-60 |
| rs17657025 | 19 | 44675801 | T | C | 0.920195 | 0.048201 | 0.013078 | 0.00023 | Age 56-60 |
| rs1004165 | 19 | 45232205 | G | A | 0.561897 | -0.04121 | 0.007128 | 7.40E-09 | Age 56-60 |
| rs11668536 | 19 | 45328476 | C | T | 0.752069 | 0.016945 | 0.008229 | 0.039 | Age 56-60 |
| rs6857 | 19 | 45392254 | C | T | 0.829355 | -0.2104 | 0.009434 | 3.50E-110 | Age 56-60 |
| rs364585 | 20 | 12962718 | A | G | 0.393233 | -0.01835 | 0.007317 | 0.012 | Age 56-60 |
| rs2328223 | 20 | 17845921 | A | C | 0.812713 | -0.01349 | 0.009097 | 0.14 | Age 56-60 |
| rs7264396 | 20 | 34154741 | C | T | 0.789996 | 0.024115 | 0.008744 | 0.0058 | Age 56-60 |
| rs6016373 | 20 | 39154095 | A | G | 0.597261 | 0.024469 | 0.007436 | 0.001 | Age 56-60 |
| rs12624863 | 20 | 39782899 | A | G | 0.789542 | -0.02481 | 0.008701 | 0.0044 | Age 56-60 |
| rs1800961 | 20 | 43042364 | C | T | 0.968364 | 0.059171 | 0.020228 | 0.0034 | Age 56-60 |
| rs13027716 | 2 | 20887226 | T | G | 0.970266 | 0.042052 | 0.021335 | 0.049 | Age 56-60 |
| rs11124924 | 2 | 21206275 | G | C | 0.938029 | 0.078322 | 0.014748 | 1.10E-07 | Age 56-60 |
| rs12720842 | 2 | 21257927 | T | C | 0.964716 | -0.06295 | 0.019243 | 0.0011 | Age 56-60 |
| rs6756743 | 2 | 21301892 | C | T | 0.952916 | -0.05748 | 0.016836 | 0.00064 | Age 56-60 |
| rs10495907 | 2 | 43998726 | G | A | 0.869004 | -0.03344 | 0.010516 | 0.0015 | Age 56-60 |
| rs3923912 | 2 | 44057570 | A | C | 0.938554 | 0.106682 | 0.014877 | 7.50E-13 | Age 56-60 |
| rs4148217 | 2 | 44099433 | C | A | 0.817094 | 0.035013 | 0.009141 | 0.00013 | Age 56-60 |
| rs11125936 | 2 | 62871225 | T | C | 0.90401 | 0.026763 | 0.011975 | 0.025 | Age 56-60 |
| rs17508045 | 2 | 1.19E+08 | T | C | 0.91285 | 0.046884 | 0.012527 | 0.00018 | Age 56-60 |
| rs16831243 | 2 | 1.36E+08 | C | T | 0.900158 | -0.02085 | 0.011886 | 0.079 | Age 56-60 |
| rs10195252 | 2 | 1.66E+08 | T | C | 0.593882 | 0.003269 | 0.007224 | 0.65 | Age 56-60 |
| rs1250229 | 2 | 2.16E+08 | T | C | 0.260933 | -0.01296 | 0.008071 | 0.11 | Age 56-60 |
| rs11563251 | 2 | 2.35E+08 | C | T | 0.888907 | -0.02058 | 0.011248 | 0.067 | Age 56-60 |
| rs4823057 | 22 | 30241061 | A | G | 0.976879 | 0.02294 | 0.023814 | 0.34 | Age 56-60 |
| rs4253772 | 22 | 46627603 | C | T | 0.889457 | -0.00405 | 0.011257 | 0.72 | Age 56-60 |
| rs7616006 | 3 | 12267648 | A | G | 0.57829 | 0.015517 | 0.007188 | 0.031 | Age 56-60 |
| rs7640978 | 3 | 32533010 | C | T | 0.91092 | 0.03266 | 0.012461 | 0.0088 | Age 56-60 |
| rs17404153 | 3 | 1.32E+08 | G | T | 0.876045 | 0.005783 | 0.010811 | 0.59 | Age 56-60 |
| rs6818397 | 4 | 3434885 | T | G | 0.386914 | 0.015579 | 0.007345 | 0.034 | Age 56-60 |
| rs4072296 | 5 | 74242002 | T | C | 0.458183 | -0.02495 | 0.007358 | 7.00E-04 | Age 56-60 |
| rs16872670 | 5 | 74929312 | G | A | 0.952478 | -0.04963 | 0.016598 | 0.0028 | Age 56-60 |
| rs4530754 | 5 | 1.23E+08 | G | A | 0.454525 | -0.02845 | 0.007086 | 6.00E-05 | Age 56-60 |
| rs4704810 | 5 | 1.56E+08 | G | A | 0.627751 | -0.00786 | 0.007407 | 0.29 | Age 56-60 |
| rs2294266 | 6 | 16107867 | A | C | 0.508267 | 0.01294 | 0.00714 | 0.07 | Age 56-60 |
| rs1408272 | 6 | 25842951 | T | G | 0.923421 | 0.062857 | 0.013396 | 2.70E-06 | Age 56-60 |
| rs1049281 | 6 | 31236567 | T | C | 0.35575 | -0.01847 | 0.007437 | 0.013 | Age 56-60 |
| rs3798236 | 6 | 1.16E+08 | T | C | 0.636079 | 0.021559 | 0.007391 | 0.0035 | Age 56-60 |
| rs6917747 | 6 | 1.6E+08 | G | A | 0.864071 | -0.04598 | 0.010342 | 8.80E-06 | Age 56-60 |
| rs9347386 | 6 | 1.61E+08 | A | G | 0.620374 | 0.031886 | 0.007336 | 1.40E-05 | Age 56-60 |
| rs6415084 | 6 | 1.61E+08 | T | C | 0.482092 | 0.014179 | 0.00714 | 0.047 | Age 56-60 |
| rs2390536 | 7 | 21485397 | G | A | 0.636938 | -0.01849 | 0.007354 | 0.012 | Age 56-60 |
| rs4722551 | 7 | 25991826 | T | C | 0.843469 | -0.03093 | 0.009771 | 0.0015 | Age 56-60 |
| rs7798185 | 7 | 44570717 | C | A | 0.829099 | -0.03466 | 0.009438 | 0.00024 | Age 56-60 |
| rs713286 | 8 | 9171735 | T | C | 0.922466 | 0.043337 | 0.013515 | 0.0013 | Age 56-60 |
| rs10102164 | 8 | 55421614 | G | A | 0.789233 | -0.01774 | 0.008758 | 0.043 | Age 56-60 |
| rs2737229 | 8 | 1.17E+08 | A | C | 0.698504 | 0.014045 | 0.00774 | 0.07 | Age 56-60 |
| rs17320616 | 8 | 1.26E+08 | A | G | 0.797943 | -0.02927 | 0.008878 | 0.00098 | Age 56-60 |
| rs7014582 | 8 | 1.45E+08 | A | G | 0.564141 | -0.00596 | 0.007191 | 0.41 | Age 56-60 |
| rs3780181 | 9 | 2640759 | A | G | 0.931586 | 0.051692 | 0.014281 | 0.00029 | Age 56-60 |
| rs12686004 | 9 | 1.08E+08 | G | A | 0.886634 | 0.033769 | 0.011274 | 0.0027 | Age 56-60 |
| rs7849542 | 9 | 1.36E+08 | G | A | 0.700579 | -0.017 | 0.007801 | 0.029 | Age 56-60 |
| rs7030248 | 9 | 1.36E+08 | G | A | 0.657589 | 0.026259 | 0.007508 | 0.00047 | Age 56-60 |
| rs1129555 | 10 | 1.14E+08 | A | G | 0.274766 | 0.023281 | 0.007378 | 0.0016 | Age 61-65 |
| rs10128711 | 11 | 18632984 | T | C | 0.261951 | -0.02476 | 0.007449 | 0.00089 | Age 61-65 |
| rs149803 | 11 | 61539020 | C | G | 0.742539 | -0.0218 | 0.007642 | 0.0043 | Age 61-65 |
| rs499790 | 11 | 1.17E+08 | C | T | 0.897778 | -0.04666 | 0.010941 | 2.00E-05 | Age 61-65 |
| rs61905078 | 11 | 1.17E+08 | A | C | 0.927217 | -0.07581 | 0.012653 | 2.10E-09 | Age 61-65 |
| rs11220437 | 11 | 1.26E+08 | T | C | 0.885254 | -0.03212 | 0.010217 | 0.0017 | Age 61-65 |
| rs3093584 | 1 | 25668714 | T | C | 0.562201 | 0.020689 | 0.006564 | 0.0016 | Age 61-65 |
| rs12753981 | 1 | 27001893 | G | A | 0.915783 | -0.01219 | 0.01177 | 0.3 | Age 61-65 |
| rs2495504 | 1 | 55485796 | C | T | 0.281642 | 0.031443 | 0.007256 | 1.50E-05 | Age 61-65 |
| rs2479408 | 1 | 55504188 | C | G | 0.787173 | 0.042576 | 0.008131 | 1.60E-07 | Age 61-65 |
| rs2495477 | 1 | 55518467 | A | G | 0.607139 | 0.036 | 0.006861 | 1.50E-07 | Age 61-65 |
| rs626787 | 1 | 62901243 | C | G | 0.646712 | 0.048359 | 0.006873 | 2.00E-12 | Age 61-65 |
| rs1386585 | 1 | 92715207 | C | T | 0.19795 | -0.00949 | 0.008192 | 0.25 | Age 61-65 |
| rs1337247 | 1 | 1.1E+08 | C | A | 0.852933 | 0.027487 | 0.009202 | 0.0028 | Age 61-65 |
| rs4268379 | 1 | 1.1E+08 | C | T | 0.500198 | -0.03634 | 0.00656 | 3.00E-08 | Age 61-65 |
| rs267733 | 1 | 1.51E+08 | A | G | 0.840039 | 0.008423 | 0.008936 | 0.35 | Age 61-65 |
| rs2642438 | 1 | 2.21E+08 | A | G | 0.297568 | -0.0245 | 0.007157 | 0.00062 | Age 61-65 |
| rs487738 | 1 | 2.35E+08 | A | G | 0.325942 | -0.02496 | 0.007391 | 0.00073 | Age 61-65 |
| rs3184504 | 12 | 1.12E+08 | T | C | 0.484821 | -0.01997 | 0.006539 | 0.0023 | Age 61-65 |
| rs2649999 | 12 | 1.21E+08 | T | C | 0.327118 | 0.024101 | 0.007094 | 0.00068 | Age 61-65 |
| rs206070 | 13 | 32896846 | G | A | 0.821946 | 0.01235 | 0.008629 | 0.15 | Age 61-65 |
| rs6573778 | 14 | 24872209 | T | C | 0.481864 | 0.020855 | 0.006572 | 0.0015 | Age 61-65 |
| rs9989419 | 16 | 56985139 | A | G | 0.398945 | 0.02315 | 0.006705 | 0.00055 | Age 61-65 |
| rs9932707 | 16 | 71862133 | A | C | 0.538912 | -0.01681 | 0.006556 | 0.01 | Age 61-65 |
| rs4788597 | 16 | 72043039 | C | T | 0.637378 | 0.025918 | 0.00682 | 0.00014 | Age 61-65 |
| rs11650232 | 17 | 7088923 | A | G | 0.554291 | -0.01563 | 0.006585 | 0.018 | Age 61-65 |
| rs7225700 | 17 | 45391804 | T | C | 0.357794 | -0.01647 | 0.00682 | 0.016 | Age 61-65 |
| rs1801689 | 17 | 64210580 | A | C | 0.968492 | -0.07049 | 0.018792 | 0.00018 | Age 61-65 |
| rs2886232 | 17 | 67150176 | T | C | 0.123057 | 0.026316 | 0.01001 | 0.0086 | Age 61-65 |
| rs17677316 | 19 | 10680241 | G | A | 0.754426 | -0.01476 | 0.007561 | 0.051 | Age 61-65 |
| rs1529711 | 19 | 11023434 | C | T | 0.840698 | -0.02296 | 0.008902 | 0.0099 | Age 61-65 |
| rs17657025 | 19 | 44675801 | T | C | 0.91762 | 0.046485 | 0.011852 | 8.80E-05 | Age 61-65 |
| rs1004165 | 19 | 45232205 | G | A | 0.563092 | -0.03715 | 0.006578 | 1.60E-08 | Age 61-65 |
| rs11668536 | 19 | 45328476 | C | T | 0.755166 | 0.028623 | 0.00757 | 0.00016 | Age 61-65 |
| rs6857 | 19 | 45392254 | C | T | 0.833988 | -0.17683 | 0.008759 | 1.20E-90 | Age 61-65 |
| rs364585 | 20 | 12962718 | A | G | 0.391831 | -0.00828 | 0.006691 | 0.22 | Age 61-65 |
| rs2328223 | 20 | 17845921 | A | C | 0.816258 | -0.03513 | 0.008463 | 3.30E-05 | Age 61-65 |
| rs7264396 | 20 | 34154741 | C | T | 0.788269 | 0.027994 | 0.008038 | 5.00E-04 | Age 61-65 |
| rs6016373 | 20 | 39154095 | A | G | 0.592721 | 0.023408 | 0.006777 | 0.00055 | Age 61-65 |
| rs12624863 | 20 | 39782899 | A | G | 0.789151 | -0.0156 | 0.007996 | 0.051 | Age 61-65 |
| rs1800961 | 20 | 43042364 | C | T | 0.9684 | 0.053494 | 0.01869 | 0.0042 | Age 61-65 |
| rs13027716 | 2 | 20887226 | T | G | 0.969574 | 0.059738 | 0.019365 | 0.002 | Age 61-65 |
| rs11124924 | 2 | 21206275 | G | C | 0.937763 | 0.054246 | 0.013503 | 5.90E-05 | Age 61-65 |
| rs12720842 | 2 | 21257927 | T | C | 0.963448 | -0.04998 | 0.017383 | 0.004 | Age 61-65 |
| rs6756743 | 2 | 21301892 | C | T | 0.952823 | -0.0464 | 0.01551 | 0.0028 | Age 61-65 |
| rs10495907 | 2 | 43998726 | G | A | 0.866957 | -0.02998 | 0.009619 | 0.0018 | Age 61-65 |
| rs3923912 | 2 | 44057570 | A | C | 0.93829 | 0.106483 | 0.013577 | 4.40E-15 | Age 61-65 |
| rs4148217 | 2 | 44099433 | C | A | 0.81911 | 0.040338 | 0.008463 | 1.90E-06 | Age 61-65 |
| rs11125936 | 2 | 62871225 | T | C | 0.908175 | 0.031558 | 0.011293 | 0.0052 | Age 61-65 |
| rs17508045 | 2 | 1.19E+08 | T | C | 0.912476 | 0.015945 | 0.011541 | 0.17 | Age 61-65 |
| rs16831243 | 2 | 1.36E+08 | C | T | 0.899298 | -0.00596 | 0.0109 | 0.58 | Age 61-65 |
| rs10195252 | 2 | 1.66E+08 | T | C | 0.590522 | 0.010674 | 0.006646 | 0.11 | Age 61-65 |
| rs1250229 | 2 | 2.16E+08 | T | C | 0.261446 | -0.00805 | 0.007466 | 0.28 | Age 61-65 |
| rs11563251 | 2 | 2.35E+08 | C | T | 0.888949 | -0.02209 | 0.010369 | 0.033 | Age 61-65 |
| rs4823057 | 22 | 30241061 | A | G | 0.977555 | -0.00289 | 0.022182 | 0.9 | Age 61-65 |
| rs4253772 | 22 | 46627603 | C | T | 0.892506 | -0.01925 | 0.010594 | 0.069 | Age 61-65 |
| rs7616006 | 3 | 12267648 | A | G | 0.57737 | 0.005189 | 0.006638 | 0.43 | Age 61-65 |
| rs7640978 | 3 | 32533010 | C | T | 0.910705 | 0.062097 | 0.011454 | 5.90E-08 | Age 61-65 |
| rs17404153 | 3 | 1.32E+08 | G | T | 0.875596 | 0.021762 | 0.009975 | 0.029 | Age 61-65 |
| rs6818397 | 4 | 3434885 | T | G | 0.386783 | 0.027017 | 0.006781 | 6.80E-05 | Age 61-65 |
| rs4072296 | 5 | 74242002 | T | C | 0.457206 | -0.02075 | 0.006764 | 0.0022 | Age 61-65 |
| rs16872670 | 5 | 74929312 | G | A | 0.952512 | -0.02724 | 0.015388 | 0.077 | Age 61-65 |
| rs4530754 | 5 | 1.23E+08 | G | A | 0.452884 | -0.01699 | 0.006563 | 0.0096 | Age 61-65 |
| rs4704810 | 5 | 1.56E+08 | G | A | 0.626798 | -0.01278 | 0.006822 | 0.061 | Age 61-65 |
| rs2294266 | 6 | 16107867 | A | C | 0.50951 | 0.016031 | 0.006543 | 0.014 | Age 61-65 |
| rs1408272 | 6 | 25842951 | T | G | 0.921622 | 0.066515 | 0.012153 | 4.40E-08 | Age 61-65 |
| rs1049281 | 6 | 31236567 | T | C | 0.354405 | -0.02244 | 0.006882 | 0.0011 | Age 61-65 |
| rs3798236 | 6 | 1.16E+08 | T | C | 0.631738 | 0.018157 | 0.006817 | 0.0077 | Age 61-65 |
| rs6917747 | 6 | 1.6E+08 | G | A | 0.861856 | -0.03652 | 0.009481 | 0.00012 | Age 61-65 |
| rs9347386 | 6 | 1.61E+08 | A | G | 0.620402 | 0.021296 | 0.006804 | 0.0017 | Age 61-65 |
| rs6415084 | 6 | 1.61E+08 | T | C | 0.481034 | 0.021505 | 0.006554 | 0.001 | Age 61-65 |
| rs2390536 | 7 | 21485397 | G | A | 0.636809 | -0.02783 | 0.006832 | 4.60E-05 | Age 61-65 |
| rs4722551 | 7 | 25991826 | T | C | 0.842568 | -0.01981 | 0.00895 | 0.027 | Age 61-65 |
| rs7798185 | 7 | 44570717 | C | A | 0.826347 | -0.03738 | 0.00866 | 1.60E-05 | Age 61-65 |
| rs713286 | 8 | 9171735 | T | C | 0.92011 | 0.014154 | 0.012264 | 0.25 | Age 61-65 |
| rs10102164 | 8 | 55421614 | G | A | 0.787602 | -0.01754 | 0.007998 | 0.028 | Age 61-65 |
| rs2737229 | 8 | 1.17E+08 | A | C | 0.695548 | 0.020228 | 0.00711 | 0.0044 | Age 61-65 |
| rs17320616 | 8 | 1.26E+08 | A | G | 0.797111 | -0.03084 | 0.008212 | 0.00017 | Age 61-65 |
| rs7014582 | 8 | 1.45E+08 | A | G | 0.563449 | -0.01005 | 0.006608 | 0.13 | Age 61-65 |
| rs3780181 | 9 | 2640759 | A | G | 0.932375 | 0.019537 | 0.013082 | 0.14 | Age 61-65 |
| rs12686004 | 9 | 1.08E+08 | G | A | 0.883889 | 0.03092 | 0.010215 | 0.0025 | Age 61-65 |
| rs7849542 | 9 | 1.36E+08 | G | A | 0.696218 | -0.00412 | 0.007125 | 0.56 | Age 61-65 |
| rs7030248 | 9 | 1.36E+08 | G | A | 0.655663 | 0.013286 | 0.006897 | 0.054 | Age 61-65 |

**Supplemental Table 5.** Multivariable MR with conditional F-statistics between SBP and LDL-C with risk of CHD

| **exposure** | **estimate** | **se** | **p** | **Nsnp*** | **Conditional F-statistics** |
| --- | --- | --- | --- | --- | --- |
| SBP |  |  |  |  |  |
| age <= 55 | 0.028722016 | 0.006322384 | 5.55E-06 | 356 | \| 4.419531 \| \| --- \| |
| age > 55 | 0.001249737 | 0.005873224 | 0.831494 |  | 3.913046 |
| LDL-C |  |  |  |  |  |
| age <= 55 | 0.518901495 | 0.16977 | 0.002239 | 325 | \| 2.958408 \| \| --- \| |
| age > 55 | -0.113074933 | 0.174687 | 0.517437 |  | 2.831518 |

*Genetic instruments were selected as independent genetic variants associated with at least one of the exposures at P<1x10^-5^. SBP: systolic blood pressure. LDL-C: low density lipoprotein cholesterol.

**Supplemental Figure 1.** Effect of genetically predicted SBP and LDL-C at different ages on risk of total CHD in univariable mendelian randomization analyses. Sensitivity analyses for MR Egger, weighted median methods and filtered instruments based on F-statistics >10 and excluding outliers by MRPRESSO. All effect estimates are given per 10 mmHg increase in SBP and 1 mmol/L increase in LDL-C

**Supplemental Figure 2.** Effect of genetically predicted SBP and LDL-C at different ages on risk of incident CHD in univariable Mendelian randomization analyses. Sensitivity analyses for MR Egger, weighted median methods and filtered instruments based on F-statistics >10 and excluding outliers by MRPRESSO. All effect estimates are given per 10 mmHg increase in SBP and 1 mmol/L increase in LDL-C. There were no outliers detected by MRPRESSOfor SBP
